# Identification of the hub susceptibility genes and related common transcription factors in the skeletal muscle of Type 2 Diabetes Mellitus

**DOI:** 10.1186/s12902-022-01195-0

**Published:** 2022-11-11

**Authors:** Jianjuan Ke, Xiaohua Hu, Changhua Wang, Yemin Zhang

**Affiliations:** 1 Department of Anesthesiology, Zhongnan Hospital of Wuhan University, Wuhan, 430071 China; 2 Department of Respiratory Medicine, Renmin Hospital of Lichuan, Lichuan, 445400 China; 3 Department of Pathology & Pathophysiology, Wuhan University Taikang Medical School (School of Basic Medical Sciences), Wuhan, 430071 China; 4 Hubei Provincial Key Laboratory of Developmentally Originated Disease, Wuhan University Taikang Medical School (School of Basic Medical Sciences), Wuhan, 430071 China; 5 Demonstration Center for Experimental Basic Medicine Education of Wuhan University Taikang Medical School (School of Basic Medical Sciences), Wuhan, 430071 China

**Keywords:** T2DM, Susceptibility differentially expressed genes (SDEG), Transcription factors, Bioinformatics, Inherited factors, Noninherited factors

## Abstract

**Background:**

Type 2 diabetes mellitus (T2DM) and its related complications contribute to the high morbidity and mortality in worldwide. Skeletal muscle insulin resistance plays a critical role in the onset of T2DM due to the decreasing in the insulin-stimulated glucose uptake. T2DM is associated not only with the inherited factors but also with the noninherited factors. However, the susceptibility genes related with the two factors and the transcription factors (TF) regulating the susceptibility genes in skeletal muscle, which aggravate the development of T2DM were still ill-defined.

**Methods:**

In the present study, the expression profiles by the array of GSE25462 were retrieved from the GEO database. GEO2R was performed to validate the susceptibility differentially expressed genes (SDEG) in skeletal muscle of T2DM. Gene Ontology (GO) analysis and The Kyoto Encyclopedia of Genes and Genomes (KEGG) pathway analysis were conducted via The Database for Annotation, Visualization, and Integrated Discovery (DAVID). A Protein-Protein Interaction (PPI) network was performed with the STRING.

**Results:**

With the performance of GEO2R, 229 SDEGs in skeletal muscle of T2DM were identified. The biological processes (BP) of SDEGs was enriched in the cellular response to UV-B most significantly. KEGG pathway analysis revealed that the SDEGs were most significantly enriched in glycosaminoglycan degradation. 5 hub susceptibility genes (*GPR84, CALCB, GCG, PTGDR, GNG8*) in the skeletal muscle of T2DM were identified. Eventually, the common transcription factors regulating the hub susceptibility genes were identified by means of the online tool PROMO.

**Conclusions:**

Five hub susceptibility genes (*GPR84, CALCB, GCG, PTGDR, GNG8*) in the skeletal muscle of T2DM and the common transcription factors were identified. The outputs would provide new clues on the novel potential targets and the therapeutic strategies for treating T2DM and its related diseases.

**Supplementary Information:**

The online version contains supplementary material available at 10.1186/s12902-022-01195-0.

## Introduction

Type 2 diabetes mellitus (T2DM) and its related complications, such as skeletal muscle atrophy, diabetic cardiomyopathy and tumors, contribute to the high morbidity and mortality in worldwide [1–3]. Meanwhile, during the pathophysiology of T2DM, a chronic metabolic disordered process, skeletal muscle plays a crucial role, mostly because skeletal muscle is one of the main insulin-sensitive tissues which uptakes glucose by responding to insulin stimulation via glucose transporter 4 (GLUT4). Skeletal muscle insulin resistance, which is defined as less sensitivity of skeletal muscle to normal insulin concentration, aggravates the development of T2DM [4]. Therefore, skeletal muscle is emerging as a promising therapeutic target for T2DM and its related metabolic disorders, such as adipose dysfunction and alcoholic fatty liver disease (NAFLD )[5].

Transcription factors (TFs) are a certain kind of cellular proteins that bind to the specific promoter regions of DNA, which lie upstream of the coding region in a gene to regulate the transcriptional machinery [6]. TF is emerging as an important driver in the pathophysiology of T2DM, and it would be of importance to identify the TF associated with inherited factors.

T2DM is a complex disease due to the interplay between the inherited factors and noninherited factors [7, 8], which makes it to be of great significance to identify the susceptibility differentially expressed genes (SDEG) in skeletal muscle of T2DM. To better figure out the mechanisms of SDEGs, the common TFs regulating the SDEGs are necessary to be identified simultaneously.

The SDEGs and the common TFs will be possibly emerging as the potential targets to treat T2DM. The identification of the SDEGs and TFs related with the interaction between the inheritance and the noninherited factors on the development of T2DM would provide new clues for the potential therapeutic strategies for the treatment of T2DM and its related disorders.

In the present study, to identify the SDEGs of T2DM, the datasets of the GSE25462 were retrieved from Gene Expression Omnibus (GEO, http://www.ncbi.nlm.nih.gov/geo/), an international public repository providing freely high-throughput microarray [9]. With the performance of the web tool GEO2R, both the differentially expressed genes (DEGs) in skeletal muscle between T2DM subjects and the normoglycemic insulin-resistant subjects with parental family history (PFH) of T2DM subjects, and the DEGs between T2DM subjects and the normoglycemic insulin resistant subjects without PFH of T2DM subjects were identified. The overlap of DEGs of the two groups was considered as the SDEGs associating with the interplay of the inheritance and the noninherited factors in skeletal muscle of T2DM. In the present study, the 5 hub susceptibility genes (*GPR84, CALCB, GCG, PTGDR, GNG8*) and the common transcription factors regulating the hub susceptibility genes in skeletal muscle were identified with the performance of the various bioinformatics methods. Our outputs would hold promise for the novel therapeutic strategies for the treatment of T2DM and its related diseases.

## Materials and methods

### Microarray data archives

To identify SDEGs in skeletal muscle of T2DM, the expression profiles by array of GSE25462 were retrieved from GEO database. The GSE25462 collected 10 female skeletal muscle samples from T2DM subjects (T2DM), 25 female skeletal muscle samples from the normoglycemic insulin resistant (IR) subjects with PFH of T2DM (PFH), and 15 female skeletal muscle samples from the normoglycemic IR subjects without PFH of T2DM (nonPFH). The BMI, fasting glucose, fasting insulin in T2DM subjects were higher than the other two groups. The BMI and fasting glucose had no difference between PFH and nonPFH subjects, However, the fasting insulin in PFH subjects were higher than the PFH subjects group (Figure S1), which were described previously in the GEO database GSE25462 (http://www.ncbi.nlm.nih.gov/geo). The expression profiling of the database was based on GPL570 (Affymetrix Human Genome U133 Plus 2.0 Array) platform. The series matrix files and the data table header descriptions of the database were downloaded from the GEO database to screen and to verify the SDEGs associating with the interplay of the inheritance and the noninherited factors in skeletal muscle of T2DM.

### SDEGs Identification

Both the DEGs in skeletal muscle between T2DM subjects and the normoglycemic insulin-resistant subjects with PFH of T2DM (PFH vs T2DM), and the DEGs between T2DM subjects and the normoglycemic insulin-resistant subjects without PFH of T2DM (non-PFH vs T2DM) were identified, respectively, with the performance of the web tool GEO2R (https://www.ncbi.nlm.nih.gov/geo/geo2r/), an online tool designed to compare the different groups of samples [10]. The p<0.05 and |log FC (fold change) | > 1 were used as cut-off criteria and defined as a statistically significant difference. The overlap of DEGs between PFH vs T2DM and non-PFH vs T2DM was considered as the SDEGs associating with the interaction of the inheritance and the noninherited factors in skeletal muscle during the development of T2DM.

### GO and pathway enrichment analyses

GO and KEGG pathway analyses of the SDEGs in skeletal muscle of T2DM were performed via The Database for Annotation, Visualization, and Integrated Discovery (DAVID 6.8, http://david.ncifcrf.gov) [11]. GO is a commonly used bioinformatic tool that provides comprehensive information on gene function of individual genomic products based on defined features. The GO analysis consists of biological processes (BP), and cellular components (CC), molecular functions (MF). KEGG is a significant database resource for understanding high-level biological functions and utilities. Gene count >2 and p < 0.05 were set as the threshold.

### The PPI network Creation and the hub susceptibility gene identification

A PPI network of the SDEGs in skeletal muscle of T2DM was constructed by Search Tool for the Retrieval of Interacting Genes (STRING10.5; https://string-db.org/) with a combined score >0.4 as the cut-off point [12]. The hub susceptibility genes were identified using Cytohubba, a plug-in of Cytoscape software (Cytoscape, 3.7.1) and the significant modules in the PPI network were identified by molecular complex detection (MCODE 1.5.1), another plug-in of Cytoscape software [13, 14]. The parameters of SDEGs clustering and scoring were set as follows: MCODE score ≥4, degree cut-off = 2, node score cut-off = 0.2, max depth = 100, and k-score = 2.

### The common transcription factors of the hub susceptibility genes prediction

Following the promotor sequences of the hub susceptibility genes were obtained from the UCSC Genome Browser database (http://genome.ucsc.edu) [15], the hub susceptibility gene promotors were submitted to the PROMO databases (Transfac 8.3), a freely available online tool, to predict the putative transcription factors binding motifs of each hub susceptibility genes [16]. The maximum matrix dissimilarity rate was set as 0**.** The overlap of the transcription factors of each hub susceptibility gene was identified as the common putative transcription factors of the hub susceptibility genes.

### Statistical analysis

The statistical analyses of DEGs were done with GEO2R (https://www.ncbi.nlm.nih.gov/geo/geo2r/), an online tool designed to compare the different groups of samples. The p<0.05 and |log FC (fold change) | > 1 were used as cut-off criteria and defined a statistically significant difference. Gene count >2 and p < 0.05 were set as the threshold in the GO and KEGG analysis with the performance of the DAVID database.

## Results

### Identification of the SDEGs in skeletal muscle of T2DM

**Fig. 1:**
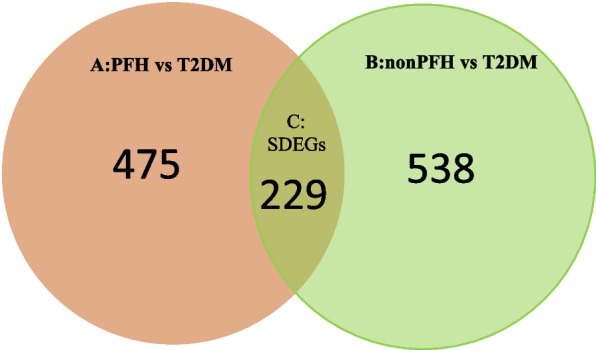
Venn diagram showing 704 DEGs were identified betweenT2DM subjects and the normoglycemic IR subjects with PFH of T2DM, and 767 DEGs were identified between T2DM subjects and the normoglycemic IR subjects without PFH of T2DM. 229 SDEGs were included in the overlap of two groups of the DEGs. **A**: PFH vs T2DM (DEGs: T2D subjects vs the normoglycemic IR subjects with PFH of T2D); **B**: nonPFH vs T2DM DEGs (T2DM subjects vs the normoglycemic IR subjects without PFH of T2DM). DEG: differentially expressed gene; **C**: SDEG: susceptibility differentially expressed gene

To identify the SDEGs in skeletal muscle of T2DM, relevant microarray expression profiles of GSE25462 was retrieved from GEO database. 704 DEGs were identified between T2DM subjects and the normoglycemic IR subjects with PFH of T2DM (PFH vs T2DM) (Table S1), and 767 DEGs were identified between T2DM subjects and the normoglycemic IR subjects without PFH of T2DM (non PFH vs T2DM) (Table S2). 229 SDEGs were included in the overlap of two groups of the DEGs in skeletal muscle of T2DM (Fig. 1, Table 1).

**Table 1:** 229 susceptibility differentially expressed genes (SDEGs) of T2DM in the skeletal muscle

Gene Symbol	ID	Gene Title
EVX1	207914_x_at	even-skipped homeobox 1
C17orf77	1553298_at	chromosome 17 open reading frame
MIR7-3HG	223973_at	MIR7-3 host gene
CDKN1A	202284_s_at	cyclin dependent kinase inhibitor 1A
TET2	1569385_s_at	tet methylcytosine dioxygenase 2
LOC101929926	237685_at	uncharacterized LOC101929926
SLC9A1	1554728_at	solute carrier family 9 member A1
NR4A1	202340_x_at	nuclear receptor subfamily 4 group A
WFDC1	219478_at	WAP four-disulfide core domain 1
NTM	241934_at	neurotrimin
ST18	1570307_s_at	ST18, C2H2C-type zinc finger
LINC00616	1561098_at	long intergenic non-protein coding R
WDR86-AS1	1560147_at	WDR86 antisense RNA 1
TMOD3	220800_s_at	tropomodulin 3
ZNF74	205881_at	zinc finger protein 74
SERPINA1	202833_s_at	serpin family A member 1
LOC100131662	236973_at	uncharacterized LOC100131662
ARSD	232423_at	arylsulfatase D
RAB15	221810_at	RAB15, member RAS oncogene family
ZNF660	1561361_at	zinc finger protein 660
GTF2A1	206521_s_at	general transcription factor IIA subunit
CARD16	1554744_at	caspase recruitment domain family me
CALCB	214636_at	calcitonin related polypeptide beta
FSTL4	1565910_at	follistatin like 4
DOK4	209690_s_at	docking protein 4
LINC00302	216935_at	long intergenic non-protein coding R
TMEM102	230633_at	transmembrane protein 102
VPS13B	1553852_at	vacuolar protein sorting 13 homolog
DICER1-AS1	1557063_at	DICER1 antisense RNA 1
STMN4	221236_s_at	stathmin 4
KCNH5	242502_at	potassium voltage-gated channel subf
TRIM14	203148_s_at	tripartite motif containing 14
PGLYRP1	207384_at	peptidoglycan recognition protein 1
NEK9	230153_at	NIMA related kinase 9
MTHFD2	234976_x_at	methylenetetrahydrofolate dehydroge
GPR78///CPZ	211062_s_at	G protein-coupled receptor 78///carb
RAB39B	238695_s_at	RAB39B, member RAS oncogene famil
TP63	211834_s_at	tumor protein p63
LYVE1	219059_s_at	lymphatic vessel endothelial hyalurona
RNF187	230662_at	ring finger protein 187
SNORA71A	1565858_at	small nucleolar RNA, H/ACA box 71A
MRPS31	243821_at	mitochondrial ribosomal protein S31
SPATA33	229660_at	spermatogenesis associated 33
LINC01395	1563070_at	long intergenic non-protein coding R
MTPN	223925_s_at	myotrophin
PCGEM1	234529_at	PCGEM1, prostate-specific transcript (
LOC101929796//	227067_x_at	notch homolog 2 N-terminal-like prot
FLJ32255	235291_s_at	uncharacterized LOC643977
TLR7	220146_at	toll like receptor 7
KCNQ1	211217_s_at	potassium voltage-gated channel subf
NFKBIE	203927_at	NFKB inhibitor epsilon
IKZF1	205038_at	IKAROS family zinc finger 1
THRA	214883_at	thyroid hormone receptor, alpha
RREB1	217411_s_at	ras responsive element binding protei
GALNT13	243779_at	polypeptide N-acetylgalactosaminyltr
ZNF391	1558658_at	zinc finger protein 391
GHSR	221360_s_at	growth hormone secretagogue recept
CD22	204581_at	CD22 molecule
PTGDR	215937_at	prostaglandin D2 receptor
ANLN	1552619_a_at	anillin actin binding protein
SLC25A14	211855_s_at	solute carrier family 25 member 14
SLC35F3	229065_at	solute carrier family 35 member F3
AOC1	203559_s_at	amine oxidase, copper containing 1
SERPINA10	220626_at	serpin family A member 10
PCBP1-AS1	1557727_at	PCBP1 antisense RNA 1
MROH9	221182_at	maestro heat like repeat family memb
DENND2D	221081_s_at	DENN domain containing 2D
CYP1B1-AS1	1553829_at	CYP1B1 antisense RNA 1
CLCA4	220026_at	chloride channel accessory 4
TAF1A-AS1	238491_at	TAF1A antisense RNA 1
CYR61	210764_s_at	cysteine rich angiogenic inducer 61
ADAMTS9	1554697_at	ADAM metallopeptidase with thromb
SYT16	239671_at	synaptotagmin 16
ZDHHC18	231900_at	zinc finger DHHC-type containing 18
HYAL3	211728_s_at	hyaluronoglucosaminidase 3
SKIDA1	1559266_s_at	SKI/DACH domain containing 1
SLC8B1	222727_s_at	solute carrier family 8 member B1
CCT8L2	220508_at	chaperonin containing TCP1 subunit 8
KLHL29	1554261_at	kelch like family member 29
PTK7	1555324_at	protein tyrosine kinase 7 (inactive)
LINC00887	1564485_at	long intergenic non-protein coding R
SPRY4-IT1	1566967_at	SPRY4 intronic transcript 1
FKBP15	231099_at	FK506 binding protein 15
PROSER2	230051_at	proline and serine rich 2
LOC100130987//	1562022_s_at	uncharacterized LOC100130987///RA
KCTD7///RABGEF	1555569_a_at	potassium channel tetramerization do
TSPYL6	231339_at	TSPY like 6
MYO5A	241966_at	myosin VA
BDNF	206382_s_at	brain derived neurotrophic factor
CALML6	1552402_at	calmodulin like 6
C3orf14	219288_at	chromosome 3 open reading frame 1
P4HA1	243335_at	prolyl 4-hydroxylase subunit alpha 1
PNPLA1	1553364_at	patatin like phospholipase domain co
CSTL1	234803_at	cystatin like 1
CTAG2	215733_x_at	cancer/testis antigen 2
GCG	206422_at	glucagon
LOC101928535	1561443_at	uncharacterized LOC101928535
UMODL1	1553183_at	uromodulin like 1
HGS	232627_at	hepatocyte growth factor-regulated ty
LOC105373460	234433_at	uncharacterized LOC105373460
KANK1	237162_at	KN motif and ankyrin repeat domains
BFSP2	207399_at	beaded filament structural protein 2
IGHM	211634_x_at	immunoglobulin heavy constant mu
TENM2	231867_at	teneurin transmembrane protein 2
DCAF5	1554558_at	DDB1 and CUL4 associated factor 5
SEMA6B	223567_at	semaphorin 6B
GPR84	223767_at	G protein-coupled receptor 84
FAM201A	1557014_a_at	family with sequence similarity 201 me
RORA	241760_x_at	RAR related orphan receptor A
LOC100509814	1562414_at	uncharacterized LOC100509814
MTBP	233436_at	MDM2 binding protein
FAM170B-AS1	1563254_a_at	FAM170B antisense RNA 1
KLRC2///KLRC1	206785_s_at	killer cell lectin like receptor C2///killer
DPP4	211478_s_at	dipeptidyl peptidase 4
ABCG5	220383_at	ATP binding cassette subfamily G me
DCDC2	222926_at	doublecortin domain containing 2
RBM47	222496_s_at	RNA binding motif protein 47
IGHV4-31///IGHA	234477_at	immunoglobulin heavy variable 4-31/
GNG8	233416_at	G protein subunit gamma 8
CD86	210895_s_at	CD86 molecule
LHFPL3-AS1	240366_at	LHFPL3 antisense RNA 1
ZBED3-AS1	1570204_at	ZBED3 antisense RNA 1
NIFK	234167_at	nucleolar protein interacting with the F
PDPN	208233_at	podoplanin
ARHGEF26-AS1	236575_at	ARHGEF26 antisense RNA 1
EFEMP1	228421_s_at	EGF containing fibulin like extracellular
IGKV1OR2-108	217378_x_at	immunoglobulin kappa variable 1/OR
CLEC12A	243106_at	C-type lectin domain family 12 memb
RUNX2	236859_at	runt related transcription factor 2
CDH12	207149_at	cadherin 12
FBXO36	1555195_at	F-box protein 36
FGF7	1555102_at	fibroblast growth factor 7
SAMSN1	220330_s_at	SAM domain, SH3 domain and nuclea
IQUB	1568924_a_at	IQ motif and ubiquitin domain contain
TTC34///LOC284	1565728_at	tetratricopeptide repeat domain 34///
CYP19A1	1554296_at	cytochrome P450 family 19 subfamily
PROZ	208034_s_at	protein Z, vitamin K dependent plasm
LOC101927809	1568633_a_at	uncharacterized LOC101927809
IL32	203828_s_at	interleukin 32
LOC284788	1557483_at	uncharacterized LOC284788
CRYBB2P1///CRY	206777_s_at	crystallin beta B2 pseudogene 1///crys
LOC374443	240572_s_at	C-type lectin domain family 2 membe
IL23A	220054_at	interleukin 23 subunit alpha
AKNAD1	1563834_a_at	AKNA domain containing 1
RASAL2	217194_at	RAS protein activator like 2
JUND	214326_x_at	JunD proto-oncogene, AP-1 transcript
LOC100996919//	207979_s_at	putative T-cell surface glycoprotein C
ANPEP	234458_at	alanyl aminopeptidase, membrane
TES	244870_at	testin LIM domain protein
LOC339807	1562776_at	uncharacterized LOC339807
RAB11FIP4	225746_at	RAB11 family interacting protein 4
FBF1	1556131_s_at	Fas binding factor 1
CAMK2A	207613_s_at	calcium/calmodulin dependent protei
SATB2-AS1	1553420_at	SATB2 antisense RNA 1
SCRG1	243984_at	stimulator of chondrogenesis 1
METTL6	1557991_at	methyltransferase like 6
HIPK1-AS1	1570080_at	HIPK1 antisense RNA 1
RFC1	208133_at	replication factor C subunit 1
IQCH	1569610_at	IQ motif containing H
CDR1	207276_at	cerebellar degeneration related protei
KCNH6	211046_at	potassium voltage-gated channel subf
RAB6B	210127_at	RAB6B, member RAS oncogene family
HTRA4	1553706_at	HtrA serine peptidase 4
MYH1	205951_at	myosin heavy chain 1
USP31	244441_at	ubiquitin specific peptidase 31
AGBL5	238889_at	ATP/GTP binding protein like 5
H2BFS	208579_x_at	H2B histone family member S
CRIP1	205081_at	cysteine rich protein 1
ANKRD22	238439_at	ankyrin repeat domain 22
LOC101928833	1562107_at	uncharacterized LOC101928833
PNLIPRP2	211766_s_at	pancreatic lipase related protein 2 (ge
MAMDC2-AS1	1559655_at	MAMDC2 antisense RNA 1
CD80	1555689_at	CD80 molecule
BAZ2A	215437_x_at	bromodomain adjacent to zinc finger
PYY	207080_s_at	peptide YY
SEMA3A	244849_at	semaphorin 3A
ADAMTS20	1553409_at	ADAM metallopeptidase with thromb
SFTA3	228979_at	surfactant associated 3
NSUN3	222886_at	NOP2/Sun RNA methyltransferase fam
NTN3	207640_x_at	netrin 3
ACTRT3	223665_at	actin related protein T3
PLEKHM3	1560069_at	pleckstrin homology domain containin
LOC100507165	236640_at	uncharacterized LOC100507165
GABRA2	1554308_s_at	gamma-aminobutyric acid type A rece
ZNF26	1555325_s_at	zinc finger protein 26
TPGS1	243600_at	tubulin polyglutamylase complex subu
PCNX4	201789_at	pecanex homolog 4 (Drosophila)
REG3G	231661_at	regenerating family member 3 gamma
LOC441454	239321_at	prothymosin, alpha pseudogene
SCGB1D2	206799_at	secretoglobin family 1D member 2
SMARCAD1	223197_s_at	SWI/SNF-related, matrix-associated ac
PPP1R1C	1555462_at	protein phosphatase 1 regulatory inhi
PCDHB13	232415_at	protocadherin beta 13
TBL1X	201867_s_at	transducin (beta)-like 1X-linked
CUL5	230393_at	cullin 5
LOC401098	1556609_at	uncharacterized LOC401098
STYX	235180_at	serine/threonine/tyrosine interacting p
KCNN2	220116_at	potassium calcium-activated channel
PWWP2A	228337_at	PWWP domain containing 2A
CCAR1	239014_at	cell division cycle and apoptosis regul
PLS1	205190_at	plastin 1
ZNF3	232497_at	zinc finger protein 3
SMIM10L2B///SM	227909_at	small integral membrane protein 10 lik
TRIM23	210994_x_at	tripartite motif containing 23
DKK3	202196_s_at	dickkopf WNT signaling pathway inhib
MFI2-AS1	242087_x_at	MFI2 antisense RNA 1
LZTFL1	222632_s_at	leucine zipper transcription factor like
WFDC6	1552396_at	WAP four-disulfide core domain 6
DGKE	1554623_x_at	diacylglycerol kinase epsilon
FGF9	239178_at	fibroblast growth factor 9
CCDC169	233298_at	coiled-coil domain containing 169
SOHLH1	1561403_at	spermatogenesis and oogenesis specif
RBMS3	242137_at	RNA binding motif single stranded int
ASPHD2	227015_at	aspartate beta-hydroxylase domain co
ZNF329	219765_at	zinc finger protein 329
LINC00674	225054_x_at	long intergenic non-protein coding R
IDS	236823_at	iduronate 2-sulfatase
LOC286177	1562365_at	uncharacterized LOC286177
HYAL4	220249_at	hyaluronoglucosaminidase 4
HOXA13	231786_at	homeobox A13
LAMA2	216839_at	laminin subunit alpha 2
C4orf33	1552372_at	chromosome 4 open reading frame 3
FLJ35934	1564383_s_at	FLJ35934
HSPD1	241716_at	heat shock protein family D (Hsp60) m
NLGN4X	221933_at	neuroligin 4, X-linked
LOC101930081	233153_at	uncharacterized LOC101930081
CLEC2D	220132_s_at	C-type lectin domain family 2 membe
TRA2B	239447_at	transformer 2 beta homolog (Drosoph
NBAS	240579_at	neuroblastoma amplified sequence

### GO enrichment analysis of SDEGs in skeletal muscle of T2DM

**Fig. 2:**
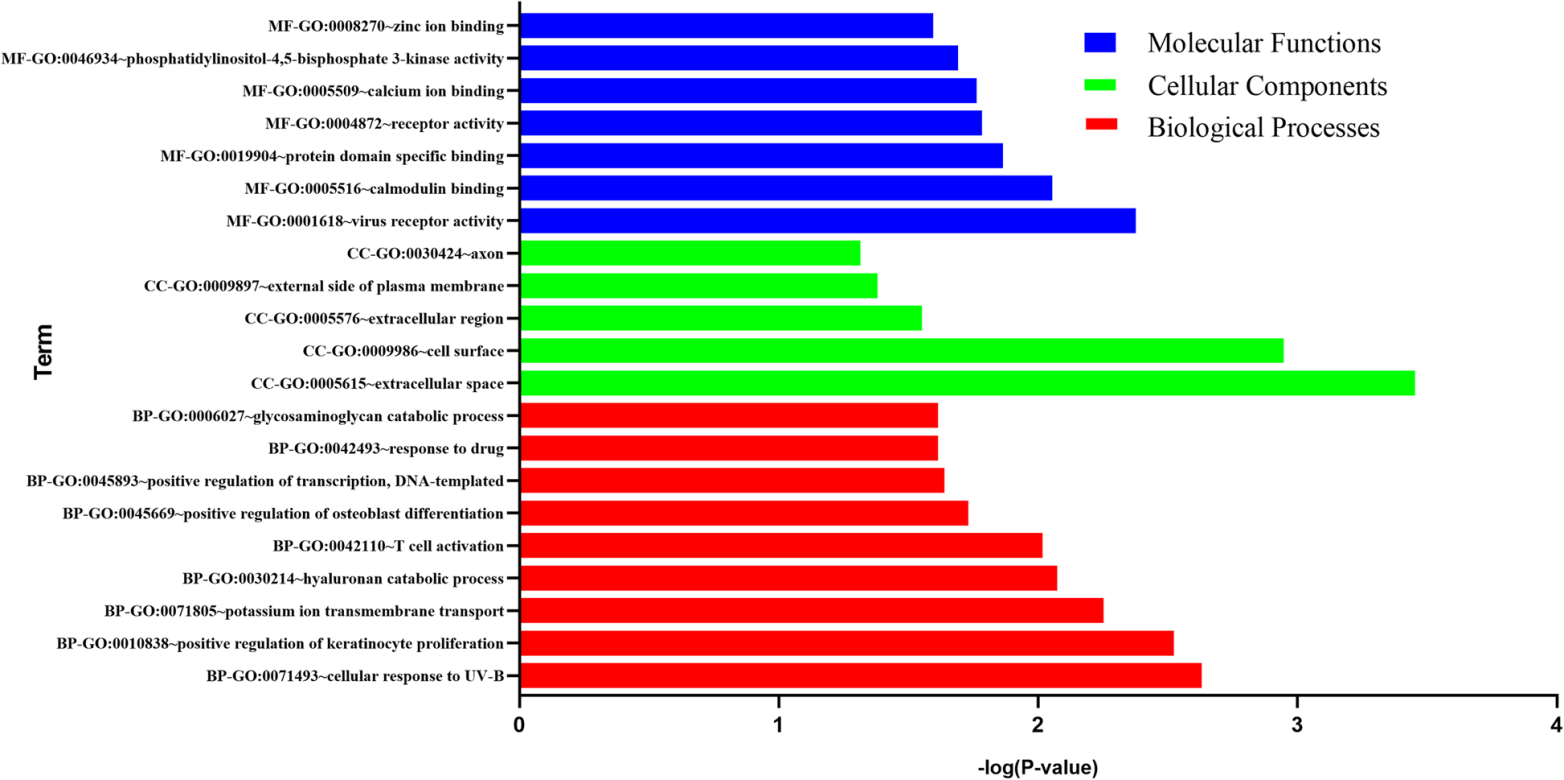
GO enrichment result of SDEGs. Abscissa represents different adjusted p value, and ordinate represents GO terms. Different colors stand for different GO classifications. GO: Gene Ontology; SDEG: susceptibility differentially expressed gene

To figure out the biological features of the SDEGs in skeletal muscle of T2DM, GO analysis was accomplished by the DAVID online tool. BP terms indicated that the SDEGs were the most significantly enriched in cellular response to UV-B and positive regulation of keratinocyte proliferation, potassium ion transmembrane transport (Fig. 2). The CC analysis showed that SDEGs were enriched in extracellular space, cell surface, extracellular region (Fig. 2,). Changes in MF of SDEGs were major enriched in zinc ion binding, heparin binding, calcium ion binding. (Fig. 2). The genes that were included GO enrichment analysis of SDEGs in skeletal muscle of T2DM were presented in Table 4.

### KEGG enrichment analysis of SDEGs in skeletal muscle of T2DM

**Fig. 3:**
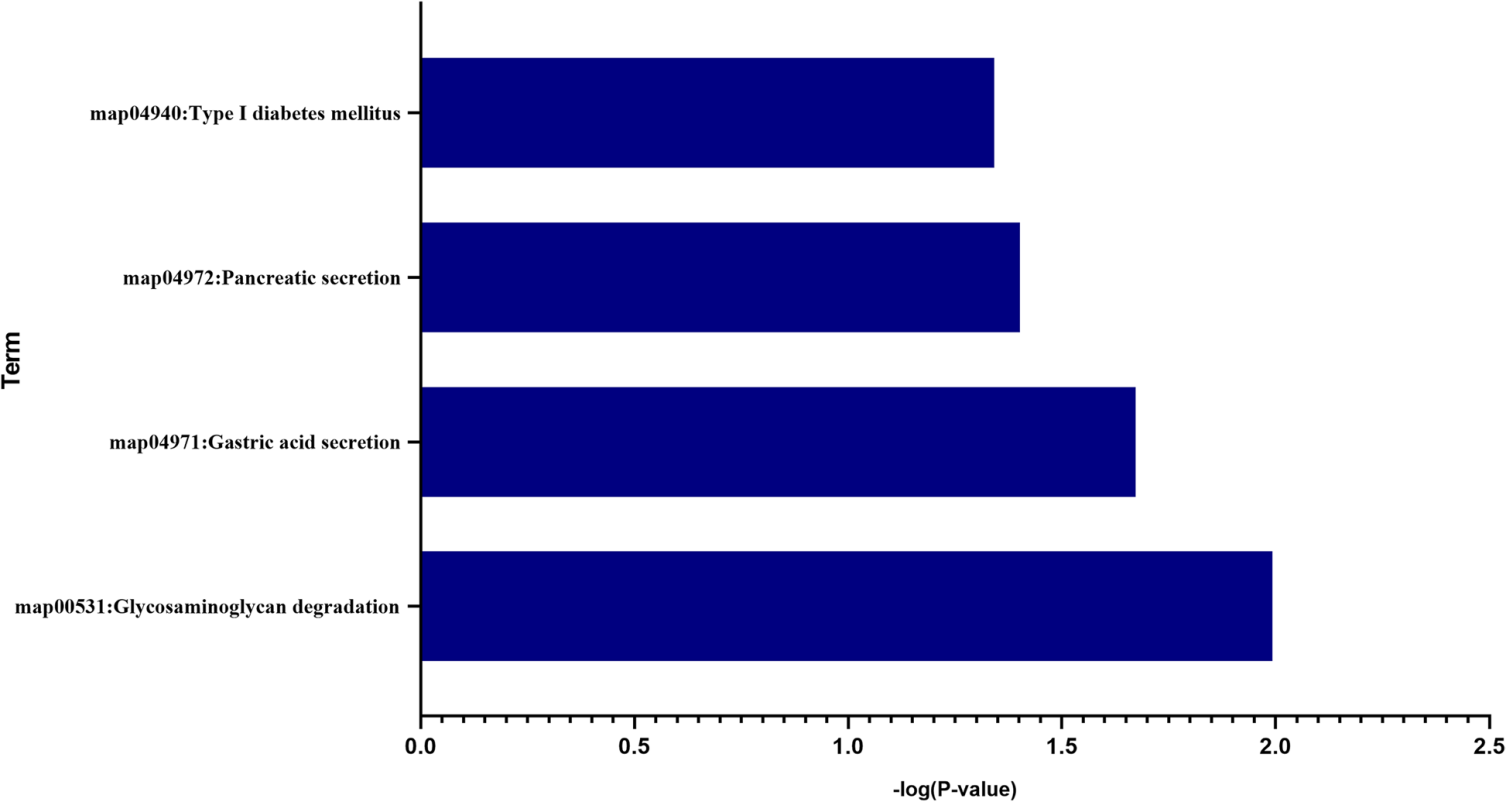
KEGG enrichment result of SDEGs. Abscissa represents different adjusted p value, and ordinate represents KEGG terms. KEGG: Kyoto Encyclopedia of Genes and Genomes SDEG: susceptibility differentially expressed gene

To explore the potential mechanism of the SDEGs in skeletal muscle of T2DM, KEGG pathway analysis was performed using DAVID online tools. KEGG analysis revealed that SDEGs were mainly involved in glycosaminoglycan degradation, gastric acid secretion and pancreatic secretion (Fig. 3). The genes that were included KEGG enrichment analysis of SDEGs in skeletal muscle of T2DM were presented in Table 5.

### Identification of the hub susceptibility genes in skeletal muscle of T2DM

**Fig. 4:**
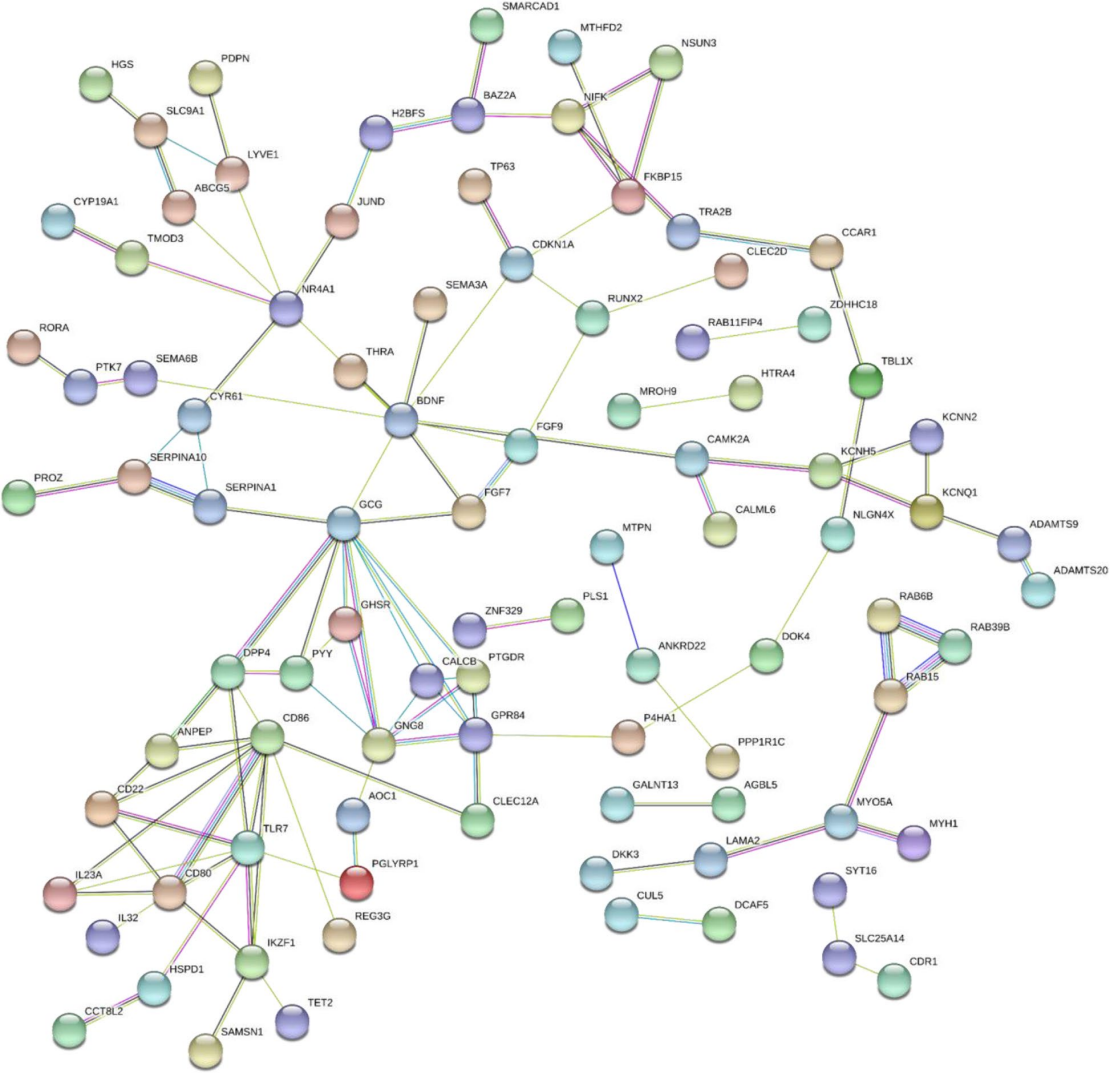
Results of the PPI network. The PPI network was analyzed by String software. Here were 176 nodes and 116 edged in the PPI network. PPI: protein–protein interaction. The color of the string maps was based on the score value with which it interacted

**Fig. 5:**
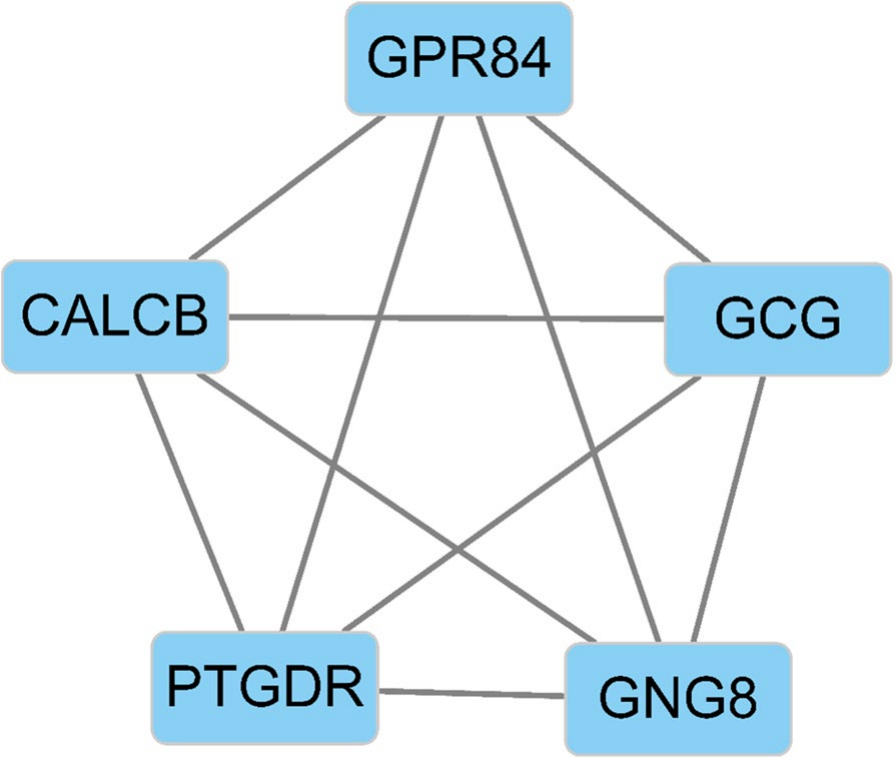
The most significant module identified by MCODE (score = 5). MCODE: molecular complex detection

To identify the most significant clusters of the SDEGs, a PPI network of SDEGs was constituted by STRING. As shown in Fig. 4, there were 176 nodes and 116 edges in the PPI network. The most significant module (score=5) was recognized by MCODE, a plug-in of Cytoscape (Fig. 5). Five hub susceptibility genes involved in SDEGs were identified, including *GPR84, CALCB, GCG, PTGDR, GNG8* (Table 2).

**Table 2:** 5 hub susceptible genes of T2DM in the skeletal muscle

Gene Symbol	ID	Gene Title
GPR84	223767_at	G protein-coupled receptor 84
CALCB	214636_at	Calcitonin related polypeptide beta
GCG	206422_at	Glucagon
PTGDR	215937_at	Prostaglandin D2 receptor
GNG8	233416_at	G protein subunit gamma 8

### Identification of the common putative transcription factors of the hub susceptibility genes in skeletal muscle of T2DM

**Fig. 6:**
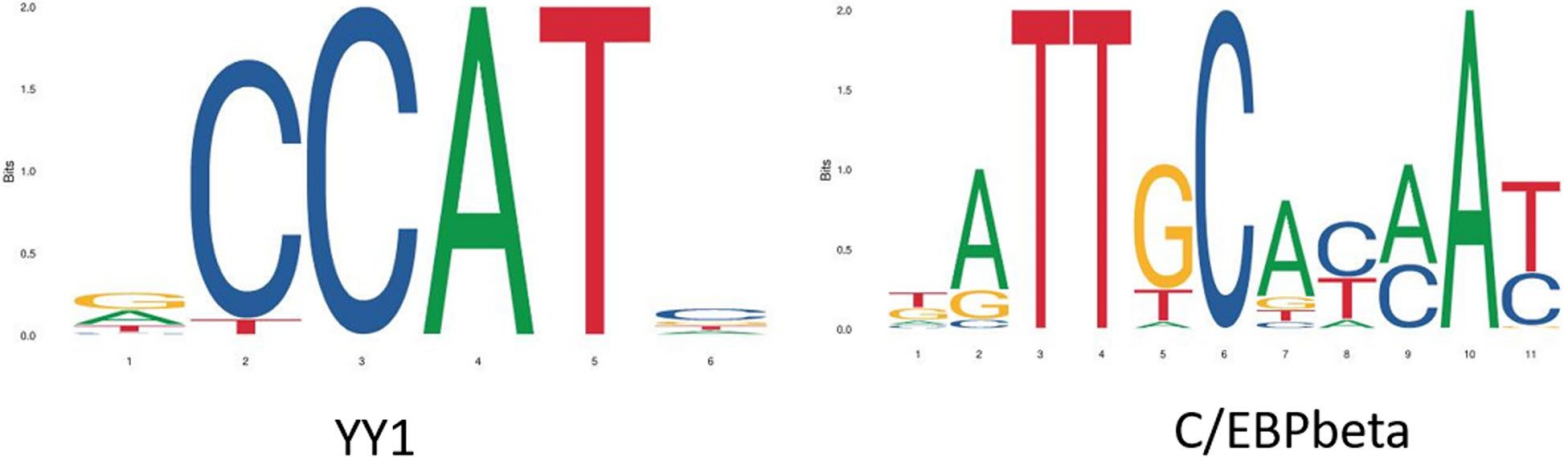
The DNA-binding sequence log of YY1, C/EBP beta in the JASPAR database

To identify the common putative transcription factors of the hub susceptibility genes in skeletal muscle of T2DM, the gene promotors were submitted to the PROMO databases (Transfac 8.3). Each hub susceptibility gene had several putative transcription factors in the PROMO databases (Table 3). The overlap of the transcription factors was identified as the common putative transcription factors of 5 hub susceptibility genes in skeletal muscle of T2DM, including *ER-alpha, YY1, GR-beta, GR-alpha, C/EBP beta, TFIID.* The DNA-binding sequence logs of *YY1* and *C/EBP* in the JASPAR database were shown in Fig. 6.

**Table 3:** Transcription factors of the nine hub genes predicted by the PROMO databases (Transfac 8.3)

GPR84	CALCB	GCG	PTDGR	GNG8
AP-2alphaA	AP-2alphaA	C/EBPbeta	AP-2alphaA	AP-2alphaA
C/EBPbeta	C/EBPalpha	ER-alpha	C/EBPbeta	C/EBPbeta
c-Ets-1	C/EBPbeta	FOXP3	c-Jun	Elk-1
ER-alpha	c-Ets-1	GATA-1	ENKTF-1	ER-alpha
FOXP3	Elk-1	GR	ER-alpha	FOXP3
GATA-1	ER-alpha	GR-alpha	FOXP3	GR-alpha
GR	GATA-1	GR-beta	GATA-1	GR-beta
GR-alpha	GR	HNF-1A	GCF	IRF-2
GR-beta	GR-alpha	HNF-3alpha	GR-alpha	Pax-5
HNF-3alpha	GR-beta	HOXD10	GR-beta	RXR-alpha
IRF-2	NFI/CTF	HOXD9	IRF-2	Sp1
NF-1	p53	IRF-2	p53	STAT4
Pax-5	Pax-5	PXR-1:RXR-alpha	Pax-5	TFIID
RXR-alpha	PR A	STAT4	PR A	TFII-I
TFIID	PR B	TBP	PR B	YY1
XBP-1	TCF-4E	TCF-4E	STAT4	
YY1	TFIID	TFIID	TBP	
	TFII-I	TFII-I	TFIID	
	XBP-1	XBP-1	TFII-I	
	YY1	YY1	XBP-1	
			YY1

**Table 4:** The genes in GO enrichment analysis of SDEGs in skeletal muscle of T2DM

Term	PValue	Genes
BP-GO:0071493~cellular response to UV-B	0.002344	CDKN1A, CRIP1, HYAL3
BP-GO:0010838~positive regulation of keratinocyte proliferation	0.002995	FGF7, TP63, REG3G
BP-GO:0071805~potassium ion transmembrane transport	0.005586	CCT8L2, KCNN2, KCNH6, KCNQ1, SLC9A1, KCNH5
CC-GO:0005615~extracellular space	3.53E-04	CSTL1, SERPINA10, FGF9, IL32, ANPEP, IGHM, MTHFD2, H2BFS, SCRG1, PROZ, SERPINA1, SEMA3A, PNLIPRP2, ADAMTS20, EFEMP1, RAB11FIP4, GCG, DKK3, ADAMTS9, UMODL1, SCGB1D2, NLGN4X, WFDC1, HSPD1, PYY, AOC1
CC-GO:0009986~cell surface	0.001131	IGHM, HYAL4, ADAMTS9, CD86, CD80, KCNN2, NLGN4X, CLEC2D, TMEM102, HSPD1, GHSR, DPP4, SLC9A1, KCNH5
CC-GO:0005576~extracellular region	0.028126	HYAL3, PNLIPRP2, FGF7, FGF9, EFEMP1, PGLYRP1, FSTL4, C17ORF77, GCG, LAMA2, DKK3, CALCB, BDNF, WFDC6, IL23A, PROZ, SFTA3, SERPINA1, HTRA4, SEMA3A, PYY, REG3G, CYR61
MF-GO:0005516~calmodulin binding	0.008803	MYO5A, MYH1, KCNN2, CAMK2A, KCNQ1, SLC9A1, KCNH5
MF-GO:0005509~calcium ion binding	0.017294	CDH12, RAB11FIP4, MYO5A, PNLIPRP2, UMODL1, TENM2, PROZ, EFEMP1, PLS1, FSTL4, PCDHB13, SYT16, CALML6, AOC1
MF-GO:0008270~zinc ion binding	0.025422	CRIP1, THRA, ADAMTS20, AGBL5, PGLYRP1, TRIM14, NR4A1, ZDHHC18, ANPEP, RNF187, RORA, TRIM23, TET2, ZNF3, ADAMTS9, ST18, AOC1, BAZ2A, TES

**Table 5:** The genes in KEGG enrichment analysis of SDEGs in skeletal muscle of T2DM

Term	PValue	Genes
hsa00531:Glycosaminoglycan degradation	0.010185	HYAL3, IDS, HYAL4
hsa04971:Gastric acid secretion	0.021273	CALML6, CAMK2A, KCNQ1, SLC9A1
hsa04972:Pancreatic secretion	0.039633	PNLIPRP2, CLCA4, KCNQ1, SLC9A1

## Discussion

The interplay of the diabetic inheritance and noninherited factors plays a critical role during the onset and pathophysiology of T2DM. In the present study, to better understand the hub susceptibility genes of the interaction of diabetic inheritance and noninherited factors in skeletal muscle on the onset and development of T2DM, bioinformatics analysis was performed.

In the present study, 229 SDEGs were identified in skeletal muscle of T2DM. The BP terms of GO analysis indicated that the SDEGs were significantly enriched in positive regulation of keratinocyte proliferation term. Although keratinocyte proliferation has been reported to be implicated in the development of T2DM [17], it was the first time to demonstrate that the genes of positive regulation of keratinocyte proliferation were involved in diabetic skeletal muscle. In the present study, the genes related with keratinocyte proliferation included FGF7, TP63, REG3G. Patrie et al. demonstrated that FGF7 was the ligands for the newt KGFR (keratinocyte growth factor receptor), which binded FGF7 with high affinity and mediates signaling in skeletal muscle myoblasts [18]. And Klasan et al. indicated that Reg3G gene played a major role in regenerating skeletal muscle [19]. Meanwhile, the CC terms indicated *FGF7* and *REG3G* were found in the extracellular region term. Maybe we could hypothesize that *FGF7* and *REG3G* could be derived from skeletal muscle cells and were transported to the keratinocyte to regulate the proliferation of the keratinocyte. The output would provide clues to explore the mechanism of diabetic keratitis.

Additionally, KEGG enrichment analysis of the SDEGs showed that these SDEGs were the most significantly mapped in the degradation of glycosaminoglycan. Glycosaminoglycan degradation could induce the decrease of proteoglycans, which were chemically diverse macromolecules to be associated with hyperglycemic conditions [20]. Yuan et al. demonstrated that glycosaminoglycan could improve insulin resistance and T2DM via enhancing liver SOD and GSH-Px activity [21]. The further connection between skeletal muscle pathology and glycosaminoglycan breakdown need to be further studied.

Based on the PPI network and the most significant module, five hub susceptibility genes in skeletal muscle of T2DM were identified in the network of SDEGs, including *GPR84, CALCB, GCG, PTGDR, GNG8. GPR84* has been reported that it was highly expressed in skeletal muscle and adipose tissue [22]. *GPR84* has been demonstrated to be involved in the regulation of energy metabolism mediated by the secretion of insulin and inflammatory responses related to insulin resistance [23]. Therefore, *GPR84* might play a crucial role during the development of skeletal muscle insulin resistance. Although Chen and his colleagues reported that *CALCB* was a calcitonin gene-related peptide which was associated with the initial events triggered in T1DM [24], up to date to our knowledge, there is no more report on *CALCB* related with insulin resistance and T2DM. *GCG* has been demonstrated to be implicated in the development of T2DM [25]. In the present study, we revealed that *GCG* alteration in the development of T2DM was regulated by both the inheritance and the noninherited factors. The previous studies on *PTGDR* were focused as an asthma susceptibility gene [26, 27]. Interestingly, metformin, which improves insulin resistance and metabolic function, has been demonstrated to play the protective role on asthma [28]. Therefore, the further study on the effect of metformin on *PTGDR* is urgent to be performed to identify whether *PTGDR* is the crosstalk target of T2DM and asthma. To the best of our knowledge, except that *GNG8* has not been reported to be implicated in the onset and development of T2DM, very few literatures reported any function of *GNG8*. It is of great significance to reveal that *GNG8* was associated with the process of T2DM with the bioinformatics tool for the first time.

To better understand the roles of the hub susceptibility genes in skeletal muscle of T2DM, the TFs of each hub SDEGs were predicted. Furthermore, the common TFs of the five hub SDEGs, including *ER-alpha, YY1, GR-beta, GR-alpha, C/EBP beta* were predicted. All the putative TFs have been reported to be implicated in diabetes and its related diseases, controlling skeletal muscle metabolism, ameliorating diabetic nephropathy pathology, modulating human pancreas development, and leading to the improvement of metabolic health and insulin sensitivity [29–31]. However, there were no previous reports on the correlation between the above five hub susceptibility genes and the putative TFs.

In the present study, the limitation focused on the absence of validation of expression on mRNA lever and the absence of further study on the exact correlation and the related mechanism.

## Conclusion

In the present study, five susceptibility hub genes and their related common TFs in skeletal muscle were identified to be related to the interplay of the inheritance and the noninherited factors on the T2DM. Furthermore, it is the first time that two genes, *PTGDR* and *GNG8,* were identified to be implicated in the development of T2DM. However, a further urgent study needs to be performed in the future to clarify the exact mechanisms of the hub susceptibility genes in skeletal muscle on the onset and development of T2DM.

## Supplementary Information

**Additional file 1: Table S1.**

**Additional file 2: Table S2.**

**Additional file 3: Figure S1.**

## Data Availability

The datasets of the GSE25462 analysed during the current study were retrieved from Gene Expression Omnibus (GEO, http://www.ncbi.nlm.nih.gov/geo/). All methods were carried out in accordance with relevant guidelines and regulations.

## Abbreviations

T2DM: Type 2 Diabetes Mellitus
IR: Insulin resistant
DEG: Differentially expressed genes
SDEG: Susceptibility differentially expressed genes
TF: Transcription factors
PFH: Parental family history
GO: Gene Ontology
KEGG: The Kyoto Encyclopedia of Genes and Genomes
DAVID: The Database for Annotation, Visualization, and Integrated Discovery
BP: Biological processes
CC: cellular components
MF: Molecular functions
PPI: Protein-Protein Interaction
