# Supplementary material for: Identification of the hub susceptibility genes and related common transcription factors in the skeletal muscle of Type 2 Diabetes Mellitus
Source: BMC Endocr Disord. 2022 Nov 11;22:276. doi: 10.1186/s12902-022-01195-0 (PMC9652898; doi:10.1186/s12902-022-01195-0)
Supplement: Supplementary file 1 — Additional file 1: Table S1. [file 12902_2022_1195_MOESM1_ESM.docx]

**Table S1. 704 DEGs between T2DM subjects and the normoglycemic IR subjects with PFH of T2DM (PFH vs T2DM)**

| **ID** | **Gene.symbol** | | **Gene.title** | | **P.Value** | | **logFC** |
| --- | --- | --- | --- | --- | --- | --- | --- |
| 1552731_at | ABRA | | actin binding Rho activating protein | | 2.49E-08 | | 1.367087 |
| 207914_x_at | EVX1 | | even-skipped homeobox 1 | | 9.27E-06 | | 1.275211 |
| 242329_at | LOC401317///CREB5 | | uncharacterized LOC401317///cAMP responsive element binding protein 5 | | 1.60E-05 | | 1.060993 |
| 1553298_at | C17orf77 | | chromosome 17 open reading frame 77 | | 1.71E-05 | | 2.083031 |
| 226140_s_at | OTUD1 | | OTU deubiquitinase 1 | | 3.96E-05 | | 1.461048 |
| 223973_at | MIR7-3HG | | MIR7-3 host gene | | 4.83E-05 | | 1.630894 |
| 231035_s_at | OTUD1 | | OTU deubiquitinase 1 | | 6.77E-05 | | 1.117467 |
| 239027_at | DOCK8 | | dedicator of cytokinesis 8 | | 8.29E-05 | | 1.928602 |
| 209794_at | SRGAP3 | | SLIT-ROBO Rho GTPase activating protein 3 | | 9.04E-05 | | 1.215008 |
| 202284_s_at | CDKN1A | | cyclin dependent kinase inhibitor 1A | | 9.89E-05 | | 1.701508 |
| 214026_s_at | SPRED2 | | sprouty related EVH1 domain containing 2 | | 9.94E-05 | | 1.272244 |
| 1569385_s_at | TET2 | | tet methylcytosine dioxygenase 2 | | 1.06E-04 | | 1.730704 |
| 237685_at | LOC101929926 | | uncharacterized LOC101929926 | | 1.32E-04 | | 1.81764 |
| 231788_at | LPAR5 | | lysophosphatidic acid receptor 5 | | 1.40E-04 | | 1.384016 |
| 1554728_at | SLC9A1 | | solute carrier family 9 member A1 | | 1.50E-04 | | 1.669991 |
| 202340_x_at | NR4A1 | | nuclear receptor subfamily 4 group A member 1 | | 1.54E-04 | | 1.238874 |
| 216984_x_at | IGLJ3///IGLV1-44///CKAP2///IGLV@///IGLC1 | | immunoglobulin lambda joining 3///immunoglobulin lambda variable 1-44///cytoskeleton associated protein 2///immunoglobulin lambda variable cluster///immunoglobulin lambda constant 1 | | 1.67E-04 | | 1.082689 |
| 236848_s_at | TEX13A | | testis expressed 13A | | 1.79E-04 | | 1.123899 |
| 240426_at | FAM47B | | family with sequence similarity 47 member B | | 1.89E-04 | | 1.625249 |
| 241441_at |  | |  | | 1.89E-04 | | 1.704696 |
| 243572_at |  | |  | | 1.94E-04 | | 1.837158 |
| 219478_at | WFDC1 | | WAP four-disulfide core domain 1 | | 2.03E-04 | | 1.535005 |
| 204144_s_at | PIGQ | | phosphatidylinositol glycan anchor biosynthesis class Q | | 2.24E-04 | | 1.187963 |
| 227858_at | PCNX3 | | pecanex homolog 3 (Drosophila) | | 2.33E-04 | | 1.056502 |
| 1567664_at |  | |  | | 2.37E-04 | | 1.994622 |
| 241934_at | NTM | | neurotrimin | | 2.43E-04 | | 1.640272 |
| 1570307_s_at | ST18 | | ST18, C2H2C-type zinc finger | | 2.52E-04 | | 1.834876 |
| 227345_at | TNFRSF10D | | TNF receptor superfamily member 10d | | 3.00E-04 | | 1.498413 |
| 211674_x_at | CTAG1A///CTAG1B | | cancer/testis antigen 1A///cancer/testis antigen 1B | | 3.74E-04 | | 1.570269 |
| 1552743_at | MCF2L | | MCF.2 cell line derived transforming sequence like | | 3.77E-04 | | 1.360413 |
| 220192_x_at | SPDEF | | SAM pointed domain containing ETS transcription factor | | 3.79E-04 | | 1.013083 |
| 213962_s_at | ANKLE2 | | ankyrin repeat and LEM domain containing 2 | | 4.33E-04 | | 1.020096 |
| 1561098_at | LINC00616 | | long intergenic non-protein coding RNA 616 | | 4.49E-04 | | 1.584339 |
| 205820_s_at | APOC3 | | apolipoprotein C3 | | 4.95E-04 | | 1.015156 |
| 215721_at | LOC100293211///IGHG1 | | uncharacterized LOC100293211///immunoglobulin heavy constant gamma 1 (G1m marker) | | 5.03E-04 | | 1.420952 |
| 1568751_at | RGS13 | | regulator of G-protein signaling 13 | | 5.20E-04 | | 2.441199 |
| 1560147_at | WDR86-AS1 | | WDR86 antisense RNA 1 | | 5.24E-04 | | 1.763074 |
| 220800_s_at | TMOD3 | | tropomodulin 3 | | 5.24E-04 | | 1.546292 |
| 215255_at | IGSF9B | | immunoglobulin superfamily member 9B | | 5.57E-04 | | 1.047226 |
| 1555371_at | ABCB5 | | ATP binding cassette subfamily B member 5 | | 5.57E-04 | | 1.61396 |
| 225305_at | SLC25A29 | | solute carrier family 25 member 29 | | 5.69E-04 | | 1.092683 |
| 217395_at | MT4 | | metallothionein 4 | | 6.36E-04 | | 1.304587 |
| 210623_at | UBXN1 | | UBX domain protein 1 | | 6.50E-04 | | 1.396074 |
| 205881_at | ZNF74 | | zinc finger protein 74 | | 6.96E-04 | | 1.231737 |
| 1559631_at |  | |  | | 6.99E-04 | | 1.311028 |
| 212292_at | SLC7A1 | | solute carrier family 7 member 1 | | 7.00E-04 | | 1.015522 |
| 205703_at | ATP6V0A2 | | ATPase H+ transporting V0 subunit a2 | | 7.10E-04 | | 1.39081 |
| 202833_s_at | SERPINA1 | | serpin family A member 1 | | 7.19E-04 | | 1.674531 |
| 238741_at | FAM83A | | family with sequence similarity 83 member A | | 7.28E-04 | | 1.028389 |
| 233446_at | ONECUT2 | | one cut homeobox 2 | | 7.44E-04 | | 1.40278 |
| 236973_at | LOC100131662 | | uncharacterized LOC100131662 | | 7.65E-04 | | 1.579235 |
| 236252_at |  | |  | | 8.04E-04 | | 1.194672 |
| 231291_at | GIPR | | gastric inhibitory polypeptide receptor | | 8.10E-04 | | 1.196106 |
| 241126_at | LOC101929529 | | uncharacterized LOC101929529 | | 8.14E-04 | | 1.299439 |
| 216821_at |  | |  | | 8.18E-04 | | 1.130963 |
| 234415_x_at |  | |  | | 8.26E-04 | | 1.239772 |
| 232423_at | ARSD | | arylsulfatase D | | 8.46E-04 | | 1.392838 |
| 221810_at | RAB15 | | RAB15, member RAS oncogene family | | 8.69E-04 | | 1.374182 |
| 204763_s_at | GNAO1 | | G protein subunit alpha o1 | | 9.22E-04 | | 1.396523 |
| 220431_at | TMPRSS11E | | transmembrane protease, serine 11E | | 9.72E-04 | | 1.611415 |
| 1561361_at | ZNF660 | | zinc finger protein 660 | | 9.84E-04 | | 1.270385 |
| 242133_s_at | LOC654342///LOC645166 | | lymphocyte-specific protein 1 pseudogene///lymphocyte-specific protein 1 pseudogene | | 9.84E-04 | | 1.475585 |
| 223810_at | KLHL1 | | kelch like family member 1 | | 1.00E-03 | | 1.675091 |
| 206521_s_at | GTF2A1 | | general transcription factor IIA subunit 1 | | 1.03E-03 | | 1.412247 |
| 227303_at | ANKS3 | | ankyrin repeat and sterile alpha motif domain containing 3 | | 1.04E-03 | | 1.217135 |
| 220337_at | NGB | | neuroglobin | | 1.06E-03 | | 1.350992 |
| 217532_x_at |  | |  | | 1.06E-03 | | 1.043469 |
| 213395_at | MLC1 | | megalencephalic leukoencephalopathy with subcortical cysts 1 | | 1.09E-03 | | 1.744639 |
| 1554744_at | CARD16 | | caspase recruitment domain family member 16 | | 1.09E-03 | | 1.249996 |
| 217639_at |  | |  | | 1.12E-03 | | 1.462925 |
| 214636_at | CALCB | | calcitonin related polypeptide beta | | 1.14E-03 | | 1.493322 |
| 1555417_a_at | TAS1R1 | | taste 1 receptor member 1 | | 1.15E-03 | | 1.111572 |
| 214248_s_at | TRIM2 | | tripartite motif containing 2 | | 1.16E-03 | | 1.033431 |
| 1565910_at | FSTL4 | | follistatin like 4 | | 1.17E-03 | | 1.662465 |
| 243583_at |  | |  | | 1.19E-03 | | 1.043864 |
| 209690_s_at | DOK4 | | docking protein 4 | | 1.19E-03 | | 1.134329 |
| 1565814_at | TRIM36 | | tripartite motif containing 36 | | 1.20E-03 | | 1.235839 |
| 216935_at | LINC00302 | | long intergenic non-protein coding RNA 302 | | 1.22E-03 | | 1.477601 |
| 230633_at | TMEM102 | | transmembrane protein 102 | | 1.26E-03 | | 1.566858 |
| 230446_at | LOC101927811 | | uncharacterized LOC101927811 | | 1.26E-03 | | 1.209259 |
| 240253_at |  | |  | | 1.27E-03 | | 1.608128 |
| 1553852_at | VPS13B | | vacuolar protein sorting 13 homolog B | | 1.28E-03 | | 1.285465 |
| 236872_at | RBM22 | | RNA binding motif protein 22 | | 1.36E-03 | | 1.049502 |
| 1557063_at | DICER1-AS1 | | DICER1 antisense RNA 1 | | 1.39E-03 | | 1.366082 |
| 1557699_x_at | |  | | 1.40E-03 | | 1.270095 | |
| 230642_at |  | |  | | 1.43E-03 | | 1.07058 |
| 204890_s_at | LCK | | LCK proto-oncogene, Src family tyrosine kinase | | 1.45E-03 | | 1.210706 |
| 221236_s_at | STMN4 | | stathmin 4 | | 1.51E-03 | | 1.035108 |
| 242502_at | KCNH5 | | potassium voltage-gated channel subfamily H member 5 | | 1.53E-03 | | 1.288104 |
| 203148_s_at | TRIM14 | | tripartite motif containing 14 | | 1.55E-03 | | 1.46957 |
| 207384_at | PGLYRP1 | | peptidoglycan recognition protein 1 | | 1.59E-03 | | 1.096201 |
| 230153_at | NEK9 | | NIMA related kinase 9 | | 1.61E-03 | | 1.281608 |
| 211794_at | FYB | | FYN binding protein | | 1.63E-03 | | 1.081469 |
| 234976_x_at | MTHFD2 | | methylenetetrahydrofolate dehydrogenase (NADP+ dependent) 2, methenyltetrahydrofolate cyclohydrolase | | 1.66E-03 | | 1.536189 |
| 212728_at | DLG3 | | discs large MAGUK scaffold protein 3 | | 1.68E-03 | | 1.088851 |
| 219447_s_at | SLC35C2 | | solute carrier family 35 member C2 | | 1.74E-03 | | 1.129794 |
| 208563_x_at | POU3F3 | | POU class 3 homeobox 3 | | 1.78E-03 | | 1.393005 |
| 205048_s_at | PSPH | | phosphoserine phosphatase | | 1.79E-03 | | 2.944911 |
| 211062_s_at | GPR78///CPZ | | G protein-coupled receptor 78///carboxypeptidase Z | | 1.80E-03 | | 1.061744 |
| 238695_s_at | RAB39B | | RAB39B, member RAS oncogene family | | 1.83E-03 | | 1.929043 |
| 1564620_at |  | |  | | 1.84E-03 | | 1.193352 |
| 222290_at | OR2A9P///OR2A20P | | olfactory receptor family 2 subfamily A member 9 pseudogene///olfactory receptor family 2 subfamily A member 20 pseudogene | | 1.86E-03 | | 1.377186 |
| 241340_at |  | |  | | 1.87E-03 | | 1.759851 |
| 211834_s_at | TP63 | | tumor protein p63 | | 1.90E-03 | | 1.206401 |
| 1566861_at | GATM | | glycine amidinotransferase | | 1.90E-03 | | 1.252053 |
| 1552452_at | WDR88 | | WD repeat domain 88 | | 1.92E-03 | | 1.008563 |
| 237303_at |  | |  | | 1.94E-03 | | 1.355821 |
| 1570250_at | PLPPR1 | | phospholipid phosphatase related 1 | | 1.95E-03 | | 1.374125 |
| 231254_at |  | |  | | 1.95E-03 | | 1.103594 |
| 239822_at | LINC01354 | | long intergenic non-protein coding RNA 1354 | | 2.02E-03 | | 1.282034 |
| 229965_at |  | |  | | 2.08E-03 | | 1.206079 |
| 236791_at |  | |  | | 2.09E-03 | | 1.098821 |
| 219059_s_at | LYVE1 | | lymphatic vessel endothelial hyaluronan receptor 1 | | 2.09E-03 | | 1.110755 |
| 214154_s_at | PKP2 | | plakophilin 2 | | 2.10E-03 | | 1.13777 |
| 216122_at |  | |  | | 2.12E-03 | | 1.044685 |
| 215448_at |  | |  | | 2.21E-03 | | 1.108222 |
| 1558768_at | DNAH1 | | dynein axonemal heavy chain 1 | | 2.22E-03 | | 1.436721 |
| 1565684_at | LOC400940 | | uncharacterized LOC400940 | | 2.29E-03 | | 1.378829 |
| 237394_at |  | |  | | 2.31E-03 | | 1.445265 |
| 243994_at |  | |  | | 2.31E-03 | | 1.075804 |
| 202238_s_at | LOC101928916///NNMT | | uncharacterized LOC101928916///nicotinamide N-methyltransferase | | 2.33E-03 | | 1.708943 |
| 230662_at | RNF187 | | ring finger protein 187 | | 2.33E-03 | | 1.298395 |
| 240137_at |  | |  | | 2.35E-03 | | 1.907634 |
| 1559229_at |  | |  | | 2.37E-03 | | 1.205755 |
| 241537_at |  | |  | | 2.37E-03 | | 1.207586 |
| 236190_at | LOC102723692 | | uncharacterized LOC102723692 | | 2.40E-03 | | 1.404826 |
| 1553053_at | LINC00521 | | long intergenic non-protein coding RNA 521 | | 2.43E-03 | | 1.164445 |
| 220462_at | CSRNP3 | | cysteine and serine rich nuclear protein 3 | | 2.46E-03 | | 1.127723 |
| 229577_at | GPAT4 | | glycerol-3-phosphate acyltransferase 4 | | 2.46E-03 | | 1.043643 |
| 1565858_at | SNORA71A | | small nucleolar RNA, H/ACA box 71A | | 2.47E-03 | | 1.263291 |
| 243821_at | MRPS31 | | mitochondrial ribosomal protein S31 | | 2.50E-03 | | 1.228874 |
| 217380_s_at | ADD3-AS1 | | ADD3 antisense RNA 1 | | 2.60E-03 | | 1.512775 |
| 226189_at | ITGB8 | | integrin subunit beta 8 | | 2.61E-03 | | 1.652779 |
| 1556329_a_at | PCDH10 | | protocadherin 10 | | 2.62E-03 | | 1.646802 |
| 229660_at | SPATA33 | | spermatogenesis associated 33 | | 2.66E-03 | | 1.469703 |
| 1563070_at | LINC01395 | | long intergenic non-protein coding RNA 1395 | | 2.67E-03 | | 1.226365 |
| 244127_at |  | |  | | 2.67E-03 | | 1.084497 |
| 232992_at | SAYSD1 | | SAYSVFN motif domain containing 1 | | 2.68E-03 | | 1.188346 |
| 223925_s_at | MTPN | | myotrophin | | 2.72E-03 | | 1.344599 |
| 1556202_at | SRGAP2 | | SLIT-ROBO Rho GTPase activating protein 2 | | 2.76E-03 | | 1.246405 |
| 1568791_s_at | EDNRB-AS1 | | EDNRB antisense RNA 1 | | 2.79E-03 | | 1.01206 |
| 204198_s_at | RUNX3 | | runt related transcription factor 3 | | 2.87E-03 | | 1.281868 |
| 234529_at | PCGEM1 | | PCGEM1, prostate-specific transcript (non-protein coding) | | 2.89E-03 | | 1.662904 |
| 227067_x_at | LOC101929796///LOC100996763///LOC100996717///NOTCH2NL///NOTCH2 | | notch homolog 2 N-terminal-like protein///notch homolog 2 N-terminal-like protein///notch homolog 2 N-terminal-like protein///notch 2 N-terminal like///notch 2 | | 2.97E-03 | | 1.253228 |
| 237661_at |  | |  | | 2.99E-03 | | 1.326197 |
| 1557624_at |  | |  | | 3.00E-03 | | 1.713678 |
| 218798_at | KRI1 | | KRI1 homolog | | 3.02E-03 | | 1.194874 |
| 210546_x_at | CTAG1A///CTAG1B | | cancer/testis antigen 1A///cancer/testis antigen 1B | | 3.03E-03 | | 1.066018 |
| 206570_s_at | PSG11 | | pregnancy specific beta-1-glycoprotein 11 | | 3.06E-03 | | 1.45442 |
| 207978_s_at | NR4A3 | | nuclear receptor subfamily 4 group A member 3 | | 3.07E-03 | | 1.32108 |
| 235291_s_at | FLJ32255 | | uncharacterized LOC643977 | | 3.11E-03 | | 1.122712 |
| 220146_at | TLR7 | | toll like receptor 7 | | 3.19E-03 | | 1.679024 |
| 1552583_s_at | ABCC13 | | ATP binding cassette subfamily C member 13 (pseudogene) | | 3.31E-03 | | 1.264983 |
| 244624_at | RPS27A | | ribosomal protein S27a | | 3.32E-03 | | 1.472201 |
| 211217_s_at | KCNQ1 | | potassium voltage-gated channel subfamily Q member 1 | | 3.37E-03 | | 1.074885 |
| 232934_at |  | |  | | 3.47E-03 | | 1.248993 |
| 203927_at | NFKBIE | | NFKB inhibitor epsilon | | 3.49E-03 | | 1.162179 |
| 207824_s_at | MAZ | | MYC associated zinc finger protein | | 3.51E-03 | | 1.157004 |
| 214128_at | DAGLA | | diacylglycerol lipase alpha | | 3.60E-03 | | 1.136795 |
| 1553637_s_at | TMCO5A | | transmembrane and coiled-coil domains 5A | | 3.60E-03 | | 1.170373 |
| 205038_at | IKZF1 | | IKAROS family zinc finger 1 | | 3.63E-03 | | 1.291778 |
| 214883_at | THRA | | thyroid hormone receptor, alpha | | 3.66E-03 | | 1.291527 |
| 236724_at | CFC1 | | cripto, FRL-1, cryptic family 1 | | 3.67E-03 | | 1.039039 |
| 205460_at | NPAS2 | | neuronal PAS domain protein 2 | | 3.71E-03 | | 1.08041 |
| 221881_s_at | CLIC4 | | chloride intracellular channel 4 | | 3.72E-03 | | 1.214784 |
| 237699_at | LINC00427 | | long intergenic non-protein coding RNA 427 | | 3.74E-03 | | 1.300499 |
| 237210_at | NFRKB | | nuclear factor related to kappaB binding protein | | 3.77E-03 | | 1.719174 |
| 217411_s_at | RREB1 | | ras responsive element binding protein 1 | | 3.85E-03 | | 1.023159 |
| 211884_s_at | CIITA | | class II major histocompatibility complex transactivator | | 3.88E-03 | | 1.387466 |
| 243779_at | GALNT13 | | polypeptide N-acetylgalactosaminyltransferase 13 | | 4.04E-03 | | 1.309117 |
| 1558658_at | ZNF391 | | zinc finger protein 391 | | 4.07E-03 | | 1.268016 |
| 221360_s_at | GHSR | | growth hormone secretagogue receptor | | 4.08E-03 | | 1.207359 |
| 217022_s_at | IGHA2///IGHA1///IGH | | immunoglobulin heavy constant alpha 2 (A2m marker)///immunoglobulin heavy constant alpha 1///immunoglobulin heavy locus | | 4.09E-03 | | 1.059206 |
| 218275_at | SLC25A10 | | solute carrier family 25 member 10 | | 4.10E-03 | | 1.363961 |
| 204581_at | CD22 | | CD22 molecule | | 4.11E-03 | | 1.009874 |
| 1569777_a_at | ZPLD1 | | zona pellucida like domain containing 1 | | 4.11E-03 | | 1.201807 |
| 215937_at | PTGDR | | prostaglandin D2 receptor | | 4.14E-03 | | 1.327372 |
| 208575_at | HIST1H3A | | histone cluster 1, H3a | | 4.22E-03 | | 1.293729 |
| 211336_x_at | LILRB1 | | leukocyte immunoglobulin like receptor B1 | | 4.29E-03 | | 1.311531 |
| 206017_at | KIAA0319 | | KIAA0319 | | 4.52E-03 | | 1.262531 |
| 236378_at | CIB4 | | calcium and integrin binding family member 4 | | 4.60E-03 | | 1.222432 |
| 1561352_at | LINC01204 | | long intergenic non-protein coding RNA 1204 | | 4.69E-03 | | 1.030597 |
| 1552619_a_at | ANLN | | anillin actin binding protein | | 4.80E-03 | | 1.129446 |
| 230273_at | CFAP206 | | cilia and flagella associated protein 206 | | 4.83E-03 | | 1.154266 |
| 211855_s_at | SLC25A14 | | solute carrier family 25 member 14 | | 4.87E-03 | | 1.150802 |
| 1557714_at | CTBP1 | | C-terminal binding protein 1 | | 5.00E-03 | | 1.232245 |
| 229065_at | SLC35F3 | | solute carrier family 35 member F3 | | 5.05E-03 | | 1.228856 |
| 203559_s_at | AOC1 | | amine oxidase, copper containing 1 | | 5.05E-03 | | 1.510565 |
| 220626_at | SERPINA10 | | serpin family A member 10 | | 5.15E-03 | | 1.155676 |
| 231553_s_at | MICAL3 | | microtubule associated monooxygenase, calponin and LIM domain containing 3 | | 5.21E-03 | | 1.093974 |
| 1557727_at | PCBP1-AS1 | | PCBP1 antisense RNA 1 | | 5.23E-03 | | 1.45755 |
| 231469_at | NTRK3-AS1 | | NTRK3 antisense RNA 1 | | 5.25E-03 | | 1.048718 |
| 221182_at | MROH9 | | maestro heat like repeat family member 9 | | 5.26E-03 | | 1.509354 |
| 221081_s_at | DENND2D | | DENN domain containing 2D | | 5.29E-03 | | 1.070677 |
| 235494_at | LSAMP | | limbic system-associated membrane protein | | 5.31E-03 | | 1.179125 |
| 211694_at | TSSK1B | | testis specific serine kinase 1B | | 5.34E-03 | | 1.067577 |
| 233913_at | WFDC10A | | WAP four-disulfide core domain 10A | | 5.38E-03 | | 1.177955 |
| 207720_at | LOR | | loricrin | | 5.48E-03 | | 1.210853 |
| 1553829_at | CYP1B1-AS1 | | CYP1B1 antisense RNA 1 | | 5.56E-03 | | 1.189511 |
| 220026_at | CLCA4 | | chloride channel accessory 4 | | 5.66E-03 | | 1.353077 |
| 1553282_at | UMODL1-AS1 | | UMODL1 antisense RNA 1 | | 5.70E-03 | | 1.098732 |
| 206500_s_at | MIS18BP1 | | MIS18 binding protein 1 | | 5.75E-03 | | 1.105477 |
| 238459_x_at | SPATA6 | | spermatogenesis associated 6 | | 5.90E-03 | | 1.354279 |
| 238491_at | TAF1A-AS1 | | TAF1A antisense RNA 1 | | 5.93E-03 | | 1.054678 |
| 213723_s_at | IDUA | | iduronidase, alpha-L- | | 5.98E-03 | | 1.003758 |
| 210764_s_at | CYR61 | | cysteine rich angiogenic inducer 61 | | 6.00E-03 | | 1.310941 |
| 1569830_at | PTPRC | | protein tyrosine phosphatase, receptor type C | | 6.01E-03 | | 1.621237 |
| 1554697_at | ADAMTS9 | | ADAM metallopeptidase with thrombospondin type 1 motif 9 | | 6.02E-03 | | 1.231112 |
| 239671_at | SYT16 | | synaptotagmin 16 | | 6.06E-03 | | 1.386055 |
| 229892_at | EP400NL | | EP400 N-terminal like | | 6.10E-03 | | 1.133053 |
| 209347_s_at | MAF | | MAF bZIP transcription factor | | 6.15E-03 | | 1.389231 |
| 231900_at | ZDHHC18 | | zinc finger DHHC-type containing 18 | | 6.18E-03 | | 1.282084 |
| 211728_s_at | HYAL3 | | hyaluronoglucosaminidase 3 | | 6.19E-03 | | 1.13165 |
| 207293_s_at | AGTR2 | | angiotensin II receptor type 2 | | 6.27E-03 | | 1.212221 |
| 1559266_s_at | SKIDA1 | | SKI/DACH domain containing 1 | | 6.27E-03 | | 1.103344 |
| 237002_at | NCDN | | neurochondrin | | 6.36E-03 | | 1.070953 |
| 240536_at |  | |  | | 6.43E-03 | | 1.161447 |
| 210882_s_at | TRO | | trophinin | | 6.45E-03 | | 1.354773 |
| 222814_s_at | ZNHIT2 | | zinc finger HIT-type containing 2 | | 6.52E-03 | | 1.703459 |
| 222727_s_at | SLC8B1 | | solute carrier family 8 member B1 | | 6.65E-03 | | 1.236782 |
| 220508_at | CCT8L2 | | chaperonin containing TCP1 subunit 8 like 2 | | 6.83E-03 | | 1.019255 |
| 1554261_at | KLHL29 | | kelch like family member 29 | | 6.84E-03 | | 1.478018 |
| 206407_s_at | CCL13 | | C-C motif chemokine ligand 13 | | 6.89E-03 | | 1.163016 |
| 1552715_a_at | RXFP1 | | relaxin/insulin like family peptide receptor 1 | | 6.94E-03 | | 1.125566 |
| 1555324_at | PTK7 | | protein tyrosine kinase 7 (inactive) | | 6.98E-03 | | 1.47863 |
| 244644_at | FAM9C | | family with sequence similarity 9 member C | | 6.99E-03 | | 1.143187 |
| 1564485_at | LINC00887 | | long intergenic non-protein coding RNA 887 | | 7.01E-03 | | 1.485633 |
| 240303_at | TMC5 | | transmembrane channel like 5 | | 7.02E-03 | | 1.284019 |
| 202463_s_at | MBD3 | | methyl-CpG binding domain protein 3 | | 7.02E-03 | | 1.089157 |
| 1566967_at | SPRY4-IT1 | | SPRY4 intronic transcript 1 | | 7.05E-03 | | 1.124314 |
| 216180_s_at | SYNJ2 | | synaptojanin 2 | | 7.13E-03 | | 1.130518 |
| 1555939_at | PRKCA-AS1 | | PRKCA antisense RNA 1 | | 7.14E-03 | | 1.728505 |
| 1563405_at | ATP4B | | ATPase H+/K+ transporting beta subunit | | 7.32E-03 | | 1.144607 |
| 1562436_at | CARS-AS1 | | CARS antisense RNA 1 | | 7.35E-03 | | 1.285883 |
| 1562895_at | LOC101927502 | | uncharacterized LOC101927502 | | 7.37E-03 | | 1.25661 |
| 1554996_at | ZNF733P///LOC285902///ZNF479///ZNF273 | | zinc finger protein 733, pseudogene///uncharacterized LOC285902///zinc finger protein 479///zinc finger protein 273 | | 7.41E-03 | | 1.634608 |
| 1567179_at | LOC105376944 | | uncharacterized LOC105376944 | | 7.44E-03 | | 1.465334 |
| 241371_at | TNFRSF10A | | TNF receptor superfamily member 10a | | 7.47E-03 | | 1.077254 |
| 231099_at | FKBP15 | | FK506 binding protein 15 | | 7.49E-03 | | 1.221494 |
| 230051_at | PROSER2 | | proline and serine rich 2 | | 7.51E-03 | | 1.160694 |
| 1562022_s_at | LOC100130987///RAD9A | | uncharacterized LOC100130987///RAD9 checkpoint clamp component A | | 7.52E-03 | | 1.048181 |
| 1555569_a_at | KCTD7///RABGEF1 | | potassium channel tetramerization domain containing 7///RAB guanine nucleotide exchange factor 1 | | 7.57E-03 | | 1.159489 |
| 1554500_a_at | RGS7 | | regulator of G-protein signaling 7 | | 7.58E-03 | | 1.803893 |
| 1560316_s_at | GLCCI1 | | glucocorticoid induced 1 | | 7.63E-03 | | 1.281133 |
| 231339_at | TSPYL6 | | TSPY like 6 | | 7.63E-03 | | 1.460343 |
| 241966_at | MYO5A | | myosin VA | | 7.69E-03 | | 1.16287 |
| 1554165_at | FAM53B | | family with sequence similarity 53 member B | | 7.72E-03 | | 1.239984 |
| 206382_s_at | BDNF | | brain derived neurotrophic factor | | 7.78E-03 | | 1.184105 |
| 217248_s_at | SLC7A8 | | solute carrier family 7 member 8 | | 7.90E-03 | | 1.501945 |
| 1552402_at | CALML6 | | calmodulin like 6 | | 8.08E-03 | | 1.57112 |
| 219288_at | C3orf14 | | chromosome 3 open reading frame 14 | | 8.08E-03 | | 1.055273 |
| 243335_at | P4HA1 | | prolyl 4-hydroxylase subunit alpha 1 | | 8.09E-03 | | 1.347381 |
| 210206_s_at | DDX11 | | DEAD/H-box helicase 11 | | 8.12E-03 | | 1.454167 |
| 230776_at | RNF157-AS1 | | RNF157 antisense RNA 1 | | 8.21E-03 | | 1.382243 |
| 1556058_s_at | SPEN | | spen family transcriptional repressor | | 8.24E-03 | | 1.169269 |
| 1557693_at | SMC5-AS1 | | SMC5 antisense RNA 1 (head to head) | | 8.35E-03 | | 1.033082 |
| 240967_at | KRTAP19-3 | | keratin associated protein 19-3 | | 8.36E-03 | | 1.080963 |
| 235776_x_at | LINC00475 | | long intergenic non-protein coding RNA 475 | | 8.44E-03 | | 1.049537 |
| 1559427_at | MCF2L | | MCF.2 cell line derived transforming sequence like | | 8.44E-03 | | 1.125865 |
| 204818_at | HSD17B2 | | hydroxysteroid 17-beta dehydrogenase 2 | | 8.61E-03 | | 1.116912 |
| 1561625_at | LOC101928565 | | uncharacterized LOC101928565 | | 8.75E-03 | | 1.078135 |
| 1553364_at | PNPLA1 | | patatin like phospholipase domain containing 1 | | 8.76E-03 | | 1.480411 |
| 238355_at |  | |  | | 8.79E-03 | | 1.254656 |
| 234803_at | CSTL1 | | cystatin like 1 | | 9.01E-03 | | 1.135083 |
| 1566337_x_at | CHML | | CHM like, Rab escort protein 2 | | 9.14E-03 | | 1.737211 |
| 216785_at |  | |  | | 9.16E-03 | | 1.371094 |
| 215733_x_at | CTAG2 | | cancer/testis antigen 2 | | 9.20E-03 | | 1.145347 |
| 1563168_at | NRG1-IT1 | | NRG1 intronic transcript 1 | | 9.22E-03 | | 1.155223 |
| 238450_at | PFKFB2 | | 6-phosphofructo-2-kinase/fructose-2,6-biphosphatase 2 | | 9.23E-03 | | 1.05624 |
| 206422_at | GCG | | glucagon | | 9.30E-03 | | 1.180548 |
| 204869_at | PCSK2 | | proprotein convertase subtilisin/kexin type 2 | | 9.35E-03 | | 1.102169 |
| 1561443_at | LOC101928535 | | uncharacterized LOC101928535 | | 9.39E-03 | | 1.172639 |
| 1568784_at | LOC102725116 | | uncharacterized LOC102725116 | | 9.40E-03 | | 1.26624 |
| 1553183_at | UMODL1 | | uromodulin like 1 | | 9.42E-03 | | 1.413677 |
| 215612_at |  | |  | | 9.42E-03 | | 1.20018 |
| 1570484_at |  | |  | | 9.43E-03 | | 1.147817 |
| 232627_at | HGS | | hepatocyte growth factor-regulated tyrosine kinase substrate | | 9.53E-03 | | 1.019278 |
| 244702_at |  | |  | | 9.74E-03 | | 1.017518 |
| 1559506_x_at | MIR2682///MIR137///MIR137HG | | microRNA 2682///microRNA 137///MIR137 host gene | | 9.83E-03 | | 1.407859 |
| 1554604_at | MBTPS2 | | membrane bound transcription factor peptidase, site 2 | | 9.86E-03 | | 1.315487 |
| 234433_at | LOC105373460 | | uncharacterized LOC105373460 | | 9.92E-03 | | 1.211298 |
| 1553380_at | PARP15 | | poly(ADP-ribose) polymerase family member 15 | | 9.92E-03 | | 1.489691 |
| 215702_s_at | CFTR | | cystic fibrosis transmembrane conductance regulator | | 1.00E-02 | | 1.45218 |
| 223618_at | FMN2 | | formin 2 | | 1.00E-02 | | 1.107992 |
| 237162_at | KANK1 | | KN motif and ankyrin repeat domains 1 | | 1.01E-02 | | 1.092094 |
| 211132_at | INTS3 | | integrator complex subunit 3 | | 1.01E-02 | | 1.089171 |
| 1566805_at |  | |  | | 1.02E-02 | | 1.171503 |
| 1561219_x_at | |  | | 1.03E-02 | | 1.076748 | |
| 207399_at | BFSP2 | | beaded filament structural protein 2 | | 1.03E-02 | | 1.188446 |
| 240520_at |  | |  | | 1.03E-02 | | 1.042115 |
| 211634_x_at | IGHM | | immunoglobulin heavy constant mu | | 1.04E-02 | | 1.069889 |
| 231867_at | TENM2 | | teneurin transmembrane protein 2 | | 1.04E-02 | | 1.391187 |
| 1562924_at | LOC340357 | | uncharacterized LOC340357 | | 1.05E-02 | | 1.177705 |
| 1554558_at | DCAF5 | | DDB1 and CUL4 associated factor 5 | | 1.05E-02 | | 1.124272 |
| 238047_at | ARHGAP36 | | Rho GTPase activating protein 36 | | 1.06E-02 | | 1.456249 |
| 223718_at | ACRBP | | acrosin binding protein | | 1.07E-02 | | 1.141116 |
| 1565715_at |  | |  | | 1.08E-02 | | 1.139872 |
| 1562811_at | CTD-2194D22.4 | | uncharacterized LOC101929081 | | 1.08E-02 | | 1.06772 |
| 231460_at |  | |  | | 1.08E-02 | | 1.076537 |
| 228085_at | MMP25-AS1 | | MMP25 antisense RNA 1 | | 1.08E-02 | | 1.026201 |
| 223567_at | SEMA6B | | semaphorin 6B | | 1.11E-02 | | 1.150581 |
| 206045_s_at | NOL4 | | nucleolar protein 4 | | 1.12E-02 | | 1.046371 |
| 227714_s_at | RABL6 | | RAB, member RAS oncogene family-like 6 | | 1.12E-02 | | 1.11113 |
| 223767_at | GPR84 | | G protein-coupled receptor 84 | | 1.12E-02 | | 1.455218 |
| 1555876_at | SRGAP1 | | SLIT-ROBO Rho GTPase activating protein 1 | | 1.13E-02 | | 1.112688 |
| 1557014_a_at | FAM201A | | family with sequence similarity 201 member A | | 1.13E-02 | | 1.574124 |
| 1556750_at | LOC153577 | | uncharacterized LOC153577 | | 1.14E-02 | | 1.118633 |
| 241760_x_at | RORA | | RAR related orphan receptor A | | 1.15E-02 | | 1.681726 |
| 210300_at | REM1 | | RRAD and GEM like GTPase 1 | | 1.16E-02 | | 1.192348 |
| 1561654_at |  | |  | | 1.16E-02 | | 1.104121 |
| 1562414_at | LOC100509814 | | uncharacterized LOC100509814 | | 1.16E-02 | | 1.174596 |
| 216982_x_at |  | |  | | 1.17E-02 | | 1.15146 |
| 233436_at | MTBP | | MDM2 binding protein | | 1.17E-02 | | 1.286402 |
| 231816_s_at | UBE2Q1 | | ubiquitin conjugating enzyme E2 Q1 | | 1.17E-02 | | 1.025605 |
| 222560_at | LANCL2 | | LanC like 2 | | 1.18E-02 | | 1.059232 |
| 1566156_at |  | |  | | 1.18E-02 | | 1.143663 |
| 237467_at |  | |  | | 1.18E-02 | | 1.017489 |
| 239894_at | NEBL-AS1 | | NEBL antisense RNA 1 | | 1.18E-02 | | 1.007082 |
| 209189_at | FOS | | Fos proto-oncogene, AP-1 transcription factor subunit | | 1.19E-02 | | 1.396574 |
| 1563254_a_at | FAM170B-AS1 | | FAM170B antisense RNA 1 | | 1.19E-02 | | 1.313662 |
| 1552853_at | VWA5B1 | | von Willebrand factor A domain containing 5B1 | | 1.20E-02 | | 1.196586 |
| 238817_at | RIMBP2 | | RIMS binding protein 2 | | 1.20E-02 | | 1.04172 |
| 1565805_at | FLJ37035 | | uncharacterized LOC399821 | | 1.21E-02 | | 1.147982 |
| 205697_at | SCGN | | secretagogin, EF-hand calcium binding protein | | 1.21E-02 | | 1.040528 |
| 244543_s_at | BCDIN3D-AS1 | | BCDIN3D antisense RNA 1 | | 1.21E-02 | | 1.118583 |
| 1564373_a_at | LOC283887 | | uncharacterized LOC283887 | | 1.21E-02 | | 1.010936 |
| 206785_s_at | KLRC2///KLRC1 | | killer cell lectin like receptor C2///killer cell lectin like receptor C1 | | 1.22E-02 | | 1.156621 |
| 242286_at | GRIN2A | | glutamate ionotropic receptor NMDA type subunit 2A | | 1.23E-02 | | 1.057969 |
| 233863_at | CASZ1 | | castor zinc finger 1 | | 1.23E-02 | | 1.139448 |
| 1563121_at | LOC101927650 | | uncharacterized LOC101927650 | | 1.23E-02 | | 1.266223 |
| 1556341_s_at | MAPK12 | | mitogen-activated protein kinase 12 | | 1.24E-02 | | 1.124277 |
| 1560909_x_at | |  | | 1.25E-02 | | 1.24952 | |
| 237256_at | FBXL12 | | F-box and leucine rich repeat protein 12 | | 1.25E-02 | | 1.046404 |
| 216871_at | ZDHHC17 | | zinc finger DHHC-type containing 17 | | 1.26E-02 | | 1.035744 |
| 213904_at | FRRS1L | | ferric chelate reductase 1 like | | 1.27E-02 | | 1.38316 |
| 228659_at |  | |  | | 1.27E-02 | | 1.128002 |
| 217507_at | SLC11A1 | | solute carrier family 11 member 1 | | 1.27E-02 | | 1.142724 |
| 211478_s_at | DPP4 | | dipeptidyl peptidase 4 | | 1.27E-02 | | 1.308677 |
| 241608_at |  | |  | | 1.27E-02 | | 1.045305 |
| 240022_at | ZC3H4 | | zinc finger CCCH-type containing 4 | | 1.28E-02 | | 1.247073 |
| 1561762_s_at |  | |  | | 1.29E-02 | | 1.09972 |
| 229625_at | GBP5 | | guanylate binding protein 5 | | 1.29E-02 | | 1.330477 |
| 220383_at | ABCG5 | | ATP binding cassette subfamily G member 5 | | 1.30E-02 | | 1.179349 |
| 222926_at | DCDC2 | | doublecortin domain containing 2 | | 1.31E-02 | | 1.582346 |
| 207731_at |  | |  | | 1.31E-02 | | 1.513549 |
| 229629_at |  | |  | | 1.32E-02 | | 1.250614 |
| 232875_at | FOCAD | | focadhesin | | 1.33E-02 | | 1.145996 |
| 207546_at | ATP4B | | ATPase H+/K+ transporting beta subunit | | 1.33E-02 | | 1.053987 |
| 222496_s_at | RBM47 | | RNA binding motif protein 47 | | 1.33E-02 | | 1.08096 |
| 1555745_a_at | LYZ | | lysozyme | | 1.34E-02 | | 1.086743 |
| 234799_at | ADARB1 | | adenosine deaminase, RNA specific B1 | | 1.34E-02 | | 1.178106 |
| 207398_at | HOXD13 | | homeobox D13 | | 1.35E-02 | | 1.154859 |
| 214312_at | FOXA2 | | forkhead box A2 | | 1.36E-02 | | 1.250897 |
| 239803_at |  | |  | | 1.36E-02 | | 1.182891 |
| 1556964_s_at |  | |  | | 1.36E-02 | | 1.248567 |
| 1557211_a_at | FAM181A-AS1 | | FAM181A antisense RNA 1 | | 1.37E-02 | | 1.253129 |
| 240986_at | LOC101928943 | | uncharacterized LOC101928943 | | 1.37E-02 | | 1.027842 |
| 1561469_at | LOC101928865 | | uncharacterized LOC101928865 | | 1.38E-02 | | 1.522852 |
| 1559543_at | LINC00441 | | long intergenic non-protein coding RNA 441 | | 1.38E-02 | | 1.042093 |
| 222950_at | NIPAL2 | | NIPA like domain containing 2 | | 1.39E-02 | | 1.164252 |
| 234477_at | IGHV4-31///IGHA1 | | immunoglobulin heavy variable 4-31///immunoglobulin heavy constant alpha 1 | | 1.39E-02 | | 1.002076 |
| 223579_s_at | APOB | | apolipoprotein B | | 1.40E-02 | | 1.323358 |
| 1555502_at | NPSA | | novel prostate-specific antigen | | 1.40E-02 | | 1.103846 |
| 233416_at | GNG8 | | G protein subunit gamma 8 | | 1.41E-02 | | 1.070719 |
| 1560823_at | LOC340017 | | uncharacterized LOC340017 | | 1.41E-02 | | 1.352078 |
| 222281_s_at | LOC101929219///LOC100505650///C1orf186 | | uncharacterized LOC101929219///uncharacterized LOC100505650///chromosome 1 open reading frame 186 | | 1.41E-02 | | 1.105783 |
| 215876_at |  | |  | | 1.42E-02 | | 1.103893 |
| 212466_at | SPRED2 | | sprouty related EVH1 domain containing 2 | | 1.42E-02 | | 1.059966 |
| 205960_at | PDK4 | | pyruvate dehydrogenase kinase 4 | | 1.42E-02 | | 1.449506 |
| 238226_at | TMEM255B | | transmembrane protein 255B | | 1.42E-02 | | 1.215042 |
| 207804_s_at | FCN2 | | ficolin 2 | | 1.42E-02 | | 1.024479 |
| 219466_s_at | APOA2 | | apolipoprotein A2 | | 1.42E-02 | | 1.134253 |
| 210895_s_at | CD86 | | CD86 molecule | | 1.43E-02 | | 1.041456 |
| 1564672_at |  | |  | | 1.44E-02 | | 1.394689 |
| 238033_at | HEXDC | | hexosaminidase D | | 1.45E-02 | | 1.073712 |
| 240366_at | LHFPL3-AS1 | | LHFPL3 antisense RNA 1 | | 1.47E-02 | | 1.188776 |
| 1570204_at | ZBED3-AS1 | | ZBED3 antisense RNA 1 | | 1.47E-02 | | 1.206636 |
| 239278_at | BCAS4 | | breast carcinoma amplified sequence 4 | | 1.48E-02 | | 1.136005 |
| 1557633_at | POM121L8P | | POM121 transmembrane nucleoporin like 8, pseudogene | | 1.48E-02 | | 1.010662 |
| 1562067_at |  | |  | | 1.48E-02 | | 1.050663 |
| 232649_at | GLDN | | gliomedin | | 1.49E-02 | | 1.05396 |
| 1563595_at | SRGAP3 | | SLIT-ROBO Rho GTPase activating protein 3 | | 1.50E-02 | | 1.157644 |
| 210600_s_at | GRK4 | | G protein-coupled receptor kinase 4 | | 1.51E-02 | | 1.399062 |
| 234121_at | LOC100129069 | | uncharacterized LOC100129069 | | 1.51E-02 | | 1.092222 |
| 1570482_at |  | |  | | 1.52E-02 | | 1.009721 |
| 226961_at | PRR15 | | proline rich 15 | | 1.52E-02 | | 1.028966 |
| 234167_at | NIFK | | nucleolar protein interacting with the FHA domain of MKI67 | | 1.53E-02 | | 1.079458 |
| 241162_at |  | |  | | 1.53E-02 | | 1.25734 |
| 208233_at | PDPN | | podoplanin | | 1.54E-02 | | 1.049789 |
| 233337_s_at | SEZ6L2 | | seizure related 6 homolog like 2 | | 1.55E-02 | | 1.024882 |
| 216234_s_at | PRKACA | | protein kinase cAMP-activated catalytic subunit alpha | | 1.55E-02 | | 1.098766 |
| 1570181_a_at | |  | | 1.55E-02 | | 1.49975 | |
| 1562847_at | LOC101929297 | | uncharacterized LOC101929297 | | 1.56E-02 | | 1.196282 |
| 236575_at | ARHGEF26-AS1 | | ARHGEF26 antisense RNA 1 | | 1.56E-02 | | 1.168137 |
| 228421_s_at | EFEMP1 | | EGF containing fibulin like extracellular matrix protein 1 | | 1.56E-02 | | 1.021878 |
| 217378_x_at | IGKV1OR2-108 | | immunoglobulin kappa variable 1/OR2-108 (non-functional) | | 1.57E-02 | | 1.01098 |
| 1557652_a_at | CCDC13-AS1 | | CCDC13 antisense RNA 1 | | 1.57E-02 | | 1.176108 |
| 1563128_at |  | |  | | 1.58E-02 | | 1.454795 |
| 243106_at | CLEC12A | | C-type lectin domain family 12 member A | | 1.58E-02 | | 1.025755 |
| 1561491_at | LOC283214 | | uncharacterized LOC283214 | | 1.58E-02 | | 1.12637 |
| 233598_at | C20orf187 | | chromosome 20 open reading frame 187 | | 1.58E-02 | | 1.052508 |
| 236859_at | RUNX2 | | runt related transcription factor 2 | | 1.58E-02 | | 1.130472 |
| 208564_at | KCNA2 | | potassium voltage-gated channel subfamily A member 2 | | 1.59E-02 | | 1.370103 |
| 1564697_a_at | LINC01144 | | long intergenic non-protein coding RNA 1144 | | 1.59E-02 | | 1.135937 |
| 1555244_at | LOC554207 | | uncharacterized LOC554207 | | 1.61E-02 | | 1.258875 |
| 231356_at |  | |  | | 1.61E-02 | | 1.182045 |
| 244240_at |  | |  | | 1.62E-02 | | 1.053327 |
| 223974_at | DLGAP1-AS2 | | DLGAP1 antisense RNA 2 | | 1.62E-02 | | 1.268279 |
| 207149_at | CDH12 | | cadherin 12 | | 1.63E-02 | | 1.071291 |
| 1555195_at | FBXO36 | | F-box protein 36 | | 1.63E-02 | | 1.399298 |
| 1558599_at |  | |  | | 1.63E-02 | | 1.090797 |
| 1556874_a_at | MEX3C | | mex-3 RNA binding family member C | | 1.66E-02 | | 1.115548 |
| 205462_s_at | HPCAL1 | | hippocalcin like 1 | | 1.66E-02 | | 1.383364 |
| 228699_at | NRP2 | | neuropilin 2 | | 1.67E-02 | | 1.094728 |
| 1555102_at | FGF7 | | fibroblast growth factor 7 | | 1.67E-02 | | 1.717214 |
| 220330_s_at | SAMSN1 | | SAM domain, SH3 domain and nuclear localization signals 1 | | 1.67E-02 | | 1.038488 |
| 1562822_at | LOC101928389 | | uncharacterized LOC101928389 | | 1.68E-02 | | 1.215621 |
| 240761_at |  | |  | | 1.68E-02 | | 1.013584 |
| 1555224_at | CCDC148-AS1 | | CCDC148 antisense RNA 1 | | 1.69E-02 | | 1.166207 |
| 1552833_at | B3GNT6 | | UDP-GlcNAc:betaGal beta-1,3-N-acetylglucosaminyltransferase 6 | | 1.69E-02 | | 1.064154 |
| 233769_at | LOC284561 | | uncharacterized LOC284561 | | 1.71E-02 | | 1.194946 |
| 240059_at |  | |  | | 1.71E-02 | | 1.143541 |
| 243499_at |  | |  | | 1.71E-02 | | 1.067669 |
| 208501_at | GFI1B | | growth factor independent 1B transcriptional repressor | | 1.72E-02 | | 1.191534 |
| 1568924_a_at | IQUB | | IQ motif and ubiquitin domain containing | | 1.73E-02 | | 1.084553 |
| 215249_at | RPL35A | | ribosomal protein L35a | | 1.74E-02 | | 1.1124 |
| 221414_s_at | DEFB126 | | defensin beta 126 | | 1.75E-02 | | 1.304285 |
| 243666_at | CELF4 | | CUGBP, Elav-like family member 4 | | 1.76E-02 | | 1.181382 |
| 1570398_at | FAM47E | | family with sequence similarity 47 member E | | 1.76E-02 | | 1.212613 |
| 215060_at | LOC101927610 | | uncharacterized LOC101927610 | | 1.78E-02 | | 1.279327 |
| 236622_at | PIGM | | phosphatidylinositol glycan anchor biosynthesis class M | | 1.79E-02 | | 1.102629 |
| 1565728_at | TTC34///LOC284630 | | tetratricopeptide repeat domain 34///uncharacterized LOC284630 | | 1.80E-02 | | 1.089217 |
| 1560142_at | GRIK2 | | glutamate ionotropic receptor kainate type subunit 2 | | 1.81E-02 | | 1.214924 |
| 1554296_at | CYP19A1 | | cytochrome P450 family 19 subfamily A member 1 | | 1.81E-02 | | 1.260225 |
| 208034_s_at | PROZ | | protein Z, vitamin K dependent plasma glycoprotein | | 1.81E-02 | | 1.017261 |
| 1559144_x_at | LINC00910 | | long intergenic non-protein coding RNA 910 | | 1.82E-02 | | 1.03407 |
| 1567705_at |  | |  | | 1.82E-02 | | 1.139772 |
| 1569454_a_at | LOC283352 | | uncharacterized LOC283352 | | 1.84E-02 | | 1.245626 |
| 1568633_a_at | LOC101927809 | | uncharacterized LOC101927809 | | 1.84E-02 | | 1.140105 |
| 1566869_at |  | |  | | 1.84E-02 | | 1.057667 |
| 203828_s_at | IL32 | | interleukin 32 | | 1.86E-02 | | 1.666653 |
| 1557483_at | LOC284788 | | uncharacterized LOC284788 | | 1.86E-02 | | 1.057222 |
| 1562919_at | FAM45A | | family with sequence similarity 45 member A | | 1.87E-02 | | 1.159143 |
| 229057_at | SCN2A | | sodium voltage-gated channel alpha subunit 2 | | 1.87E-02 | | 1.348489 |
| 222491_at | HGSNAT | | heparan-alpha-glucosaminide N-acetyltransferase | | 1.87E-02 | | 1.15213 |
| 222165_x_at | C9orf16 | | chromosome 9 open reading frame 16 | | 1.87E-02 | | 1.005888 |
| 1560850_at | LOC101926959 | | uncharacterized LOC101926959 | | 1.89E-02 | | 1.016473 |
| 237149_at |  | |  | | 1.90E-02 | | 1.296089 |
| 241507_x_at |  | |  | | 1.91E-02 | | 1.17343 |
| 207425_s_at | 9-Sep | | septin 9 | | 1.92E-02 | | 1.167991 |
| 1558470_at | VWA3B | | von Willebrand factor A domain containing 3B | | 1.93E-02 | | 1.060744 |
| 206155_at | ABCC2 | | ATP binding cassette subfamily C member 2 | | 1.93E-02 | | 1.284317 |
| 240893_at |  | |  | | 1.95E-02 | | 1.183664 |
| 241265_x_at |  | |  | | 1.95E-02 | | 1.026038 |
| 1557315_a_at | |  | | 1.98E-02 | | 1.08207 | |
| 243021_at | LOC102724312 | | uncharacterized LOC102724312 | | 1.99E-02 | | 1.096965 |
| 1557742_a_at | UST-AS1 | | UST antisense RNA 1 | | 2.01E-02 | | 1.334015 |
| 236255_at | PLEKHG4B | | pleckstrin homology and RhoGEF domain containing G4B | | 2.01E-02 | | 1.041929 |
| 1563743_at | LINC01555 | | long intergenic non-protein coding RNA 1555 | | 2.02E-02 | | 1.086943 |
| 217684_at | TYMS | | thymidylate synthetase | | 2.03E-02 | | 1.001971 |
| 210437_at | MAGEA9B///MAGEA9 | | MAGE family member A9B///MAGE family member A9 | | 2.03E-02 | | 1.078461 |
| 206346_at | PRLR | | prolactin receptor | | 2.04E-02 | | 1.097995 |
| 206777_s_at | CRYBB2P1///CRYBB2 | | crystallin beta B2 pseudogene 1///crystallin beta B2 | | 2.05E-02 | | 1.03087 |
| 234449_at |  | |  | | 2.07E-02 | | 1.03546 |
| 233133_at |  | |  | | 2.07E-02 | | 1.383427 |
| 1566836_at |  | |  | | 2.09E-02 | | 1.086961 |
| 240572_s_at | LOC374443 | | C-type lectin domain family 2 member D pseudogene | | 2.09E-02 | | 1.267772 |
| 224101_x_at | MRS2 | | MRS2, magnesium transporter | | 2.09E-02 | | 1.455724 |
| 215396_at | ADGRV1 | | adhesion G protein-coupled receptor V1 | | 2.09E-02 | | 1.305443 |
| 220054_at | IL23A | | interleukin 23 subunit alpha | | 2.10E-02 | | 1.286137 |
| 1569528_at | LOC105375240 | | uncharacterized LOC105375240 | | 2.10E-02 | | 1.022414 |
| 1563834_a_at | AKNAD1 | | AKNA domain containing 1 | | 2.10E-02 | | 1.238482 |
| 215846_at |  | |  | | 2.11E-02 | | 1.222629 |
| 218876_at | TPPP3 | | tubulin polymerization promoting protein family member 3 | | 2.11E-02 | | 1.38728 |
| 207723_s_at | KLRC3 | | killer cell lectin like receptor C3 | | 2.12E-02 | | 1.058163 |
| 1552906_at | FMR1NB | | fragile X mental retardation 1 neighbor | | 2.12E-02 | | 1.028119 |
| 1556086_at | HPF1 | | histone PARylation factor 1 | | 2.14E-02 | | 1.294081 |
| 241854_at | DNASE1 | | deoxyribonuclease 1 | | 2.15E-02 | | 1.030955 |
| 217194_at | RASAL2 | | RAS protein activator like 2 | | 2.15E-02 | | 1.416483 |
| 1560346_at |  | |  | | 2.16E-02 | | 1.060858 |
| 1560181_at | LDLRAD4-AS1 | | LDLRAD4 antisense RNA 1 | | 2.17E-02 | | 1.116519 |
| 233709_at |  | |  | | 2.20E-02 | | 1.014518 |
| 224056_at |  | |  | | 2.21E-02 | | 1.136342 |
| 234946_at | ENTPD6 | | ectonucleoside triphosphate diphosphohydrolase 6 (putative) | | 2.21E-02 | | 1.202056 |
| 214326_x_at | JUND | | JunD proto-oncogene, AP-1 transcription factor subunit | | 2.23E-02 | | 1.411237 |
| 207979_s_at | LOC100996919///CD8B | | putative T-cell surface glycoprotein CD8 beta-2 chain-like///CD8b molecule | | 2.23E-02 | | 1.058192 |
| 1562628_at | KRT40 | | keratin 40 | | 2.23E-02 | | 1.449753 |
| 237625_s_at |  | |  | | 2.26E-02 | | 1.228587 |
| 234458_at | ANPEP | | alanyl aminopeptidase, membrane | | 2.27E-02 | | 1.080538 |
| 240714_at |  | |  | | 2.27E-02 | | 1.118904 |
| 203290_at | HLA-DQA1 | | major histocompatibility complex, class II, DQ alpha 1 | | 2.27E-02 | | 1.437858 |
| 224232_s_at | PRELID1 | | PRELI domain containing 1 | | 2.28E-02 | | 1.16881 |
| 244870_at | TES | | testin LIM domain protein | | 2.28E-02 | | 1.067538 |
| 1562776_at | LOC339807 | | uncharacterized LOC339807 | | 2.32E-02 | | 1.077308 |
| 236421_at | ANKRD45 | | ankyrin repeat domain 45 | | 2.33E-02 | | 1.033614 |
| 224356_x_at | MS4A6A | | membrane spanning 4-domains A6A | | 2.34E-02 | | 1.060411 |
| 241660_at |  | |  | | 2.34E-02 | | 1.012163 |
| 1569826_at |  | |  | | 2.38E-02 | | 1.056748 |
| 207772_s_at | PRMT8 | | protein arginine methyltransferase 8 | | 2.38E-02 | | 1.186229 |
| 1566202_at |  | |  | | 2.39E-02 | | 1.335519 |
| 211775_x_at |  | |  | | 2.40E-02 | | 1.108158 |
| 225746_at | RAB11FIP4 | | RAB11 family interacting protein 4 | | 2.40E-02 | | 1.086079 |
| 1567380_at |  | |  | | 2.41E-02 | | 1.35236 |
| 242217_s_at | FBRS | | fibrosin | | 2.41E-02 | | 1.141194 |
| 1557037_a_at | UBAC2-AS1 | | UBAC2 antisense RNA 1 | | 2.42E-02 | | 1.246246 |
| 213085_s_at | WWC1 | | WW and C2 domain containing 1 | | 2.42E-02 | | 1.083699 |
| 243373_at |  | |  | | 2.45E-02 | | 1.216727 |
| 1556131_s_at | FBF1 | | Fas binding factor 1 | | 2.45E-02 | | 1.286937 |
| 207613_s_at | CAMK2A | | calcium/calmodulin dependent protein kinase II alpha | | 2.45E-02 | | 1.093036 |
| 1557871_at | LOC253573 | | uncharacterized LOC253573 | | 2.45E-02 | | 1.396032 |
| 1553420_at | SATB2-AS1 | | SATB2 antisense RNA 1 | | 2.46E-02 | | 1.178818 |
| 1556872_s_at | IQSEC3 | | IQ motif and Sec7 domain 3 | | 2.48E-02 | | 1.151047 |
| 204855_at | SERPINB5 | | serpin family B member 5 | | 2.50E-02 | | 1.091876 |
| 1562112_at |  | |  | | 2.50E-02 | | 1.038652 |
| 1566970_at |  | |  | | 2.50E-02 | | 1.313787 |
| 230442_at | MTHFSD | | methenyltetrahydrofolate synthetase domain containing | | 2.50E-02 | | 1.120727 |
| 239021_at | TLR6 | | toll like receptor 6 | | 2.51E-02 | | 1.277076 |
| 207138_at | PHF2 | | PHD finger protein 2 | | 2.52E-02 | | 1.108523 |
| 1563246_at | LINC01432 | | long intergenic non-protein coding RNA 1432 | | 2.53E-02 | | 1.003431 |
| 1552845_at | CLDN15 | | claudin 15 | | 2.54E-02 | | 1.033175 |
| 1559528_at | LOC100129917 | | uncharacterized LOC100129917 | | 2.54E-02 | | 1.068529 |
| 236745_at | CCDC78 | | coiled-coil domain containing 78 | | 2.54E-02 | | 1.007342 |
| 1569140_at | UBR2 | | ubiquitin protein ligase E3 component n-recognin 2 | | 2.58E-02 | | 1.347877 |
| 243984_at | SCRG1 | | stimulator of chondrogenesis 1 | | 2.59E-02 | | 1.037495 |
| 1557991_at | METTL6 | | methyltransferase like 6 | | 2.59E-02 | | 1.013672 |
| 236519_at | C9orf135 | | chromosome 9 open reading frame 135 | | 2.61E-02 | | 1.058737 |
| 1564371_a_at | CASC2 | | cancer susceptibility candidate 2 (non-protein coding) | | 2.61E-02 | | 1.037403 |
| 223316_at | CCDC3 | | coiled-coil domain containing 3 | | 2.62E-02 | | 1.055462 |
| 214742_at | CEP131 | | centrosomal protein 131 | | 2.64E-02 | | 1.03033 |
| 243348_at |  | |  | | 2.64E-02 | | 1.181094 |
| 211923_s_at | ZNF471 | | zinc finger protein 471 | | 2.65E-02 | | 1.044093 |
| 233408_at | PREX2 | | phosphatidylinositol-3,4,5-trisphosphate dependent Rac exchange factor 2 | | 2.66E-02 | | 1.075322 |
| 219607_s_at | MS4A4A | | membrane spanning 4-domains A4A | | 2.66E-02 | | 1.00871 |
| 231617_at | TEX33 | | testis expressed 33 | | 2.67E-02 | | 1.138683 |
| 1570080_at | HIPK1-AS1 | | HIPK1 antisense RNA 1 | | 2.67E-02 | | 1.457333 |
| 224077_at | WHSC1L1 | | Wolf-Hirschhorn syndrome candidate 1-like 1 | | 2.67E-02 | | 1.103768 |
| 1568907_at |  | |  | | 2.68E-02 | | 1.020666 |
| 1569886_a_at | GLB1L3 | | galactosidase beta 1 like 3 | | 2.68E-02 | | 1.449775 |
| 203886_s_at | FBLN2 | | fibulin 2 | | 2.69E-02 | | 1.102679 |
| 208133_at | RFC1 | | replication factor C subunit 1 | | 2.70E-02 | | 1.002056 |
| 1565637_at | PMP22 | | peripheral myelin protein 22 | | 2.73E-02 | | 1.220016 |
| 1566222_at |  | |  | | 2.78E-02 | | 1.196253 |
| 214907_at | CEACAM21 | | carcinoembryonic antigen related cell adhesion molecule 21 | | 2.79E-02 | | 1.023339 |
| 1569469_a_at | LHX8 | | LIM homeobox 8 | | 2.81E-02 | | 1.427275 |
| 224401_s_at | FCRL4 | | Fc receptor like 4 | | 2.82E-02 | | 1.056422 |
| 236398_s_at | LOC101927851 | | uncharacterized LOC101927851 | | 2.82E-02 | | 1.136158 |
| 243954_at | LINC00877 | | long intergenic non-protein coding RNA 877 | | 2.84E-02 | | 1.13446 |
| 215414_at | FARS2 | | phenylalanyl-tRNA synthetase 2, mitochondrial | | 2.86E-02 | | 1.039788 |
| 202075_s_at | PLTP | | phospholipid transfer protein | | 2.87E-02 | | 1.198735 |
| 207204_at | FSCN2 | | fascin actin-bundling protein 2, retinal | | 2.89E-02 | | 1.017207 |
| 1569610_at | IQCH | | IQ motif containing H | | 2.89E-02 | | 1.36573 |
| 206642_at | DSG1 | | desmoglein 1 | | 2.90E-02 | | 1.028191 |
| 210033_s_at | SPAG6 | | sperm associated antigen 6 | | 2.90E-02 | | 1.102067 |
| 215745_at |  | |  | | 2.92E-02 | | 1.038844 |
| 1564051_at |  | |  | | 2.92E-02 | | 1.033415 |
| 207276_at | CDR1 | | cerebellar degeneration related protein 1 | | 2.94E-02 | | 1.034465 |
| 1552315_at | GIMAP1 | | GTPase, IMAP family member 1 | | 2.95E-02 | | 1.087782 |
| 229479_at |  | |  | | 2.96E-02 | | 1.112395 |
| 221138_s_at |  | |  | | 2.96E-02 | | 1.079196 |
| 1553822_at | RTP1 | | receptor transporter protein 1 | | 2.96E-02 | | 1.034015 |
| 1567657_at | OR2H1 | | olfactory receptor family 2 subfamily H member 1 | | 2.98E-02 | | 1.066152 |
| 1567181_x_at | LOC105376944 | | uncharacterized LOC105376944 | | 2.98E-02 | | 1.180527 |
| 211046_at | KCNH6 | | potassium voltage-gated channel subfamily H member 6 | | 3.00E-02 | | 1.219151 |
| 1558530_at | LRTM2 | | leucine rich repeats and transmembrane domains 2 | | 3.00E-02 | | 1.056705 |
| 230021_at | TICRR | | TOPBP1 interacting checkpoint and replication regulator | | 3.02E-02 | | 1.135932 |
| 240964_at |  | |  | | 3.02E-02 | | 1.074146 |
| 216605_s_at | CEACAM21 | | carcinoembryonic antigen related cell adhesion molecule 21 | | 3.02E-02 | | 1.042641 |
| 205747_at | CBLN1 | | cerebellin 1 precursor | | 3.06E-02 | | 1.022894 |
| 243753_at | ERICH3 | | glutamate rich 3 | | 3.10E-02 | | 1.122674 |
| 1555177_at | PRKAA1 | | protein kinase AMP-activated catalytic subunit alpha 1 | | 3.12E-02 | | 1.12364 |
| 223949_at | TMPRSS3 | | transmembrane protease, serine 3 | | 3.13E-02 | | 1.035292 |
| 244860_at |  | |  | | 3.13E-02 | | 1.020672 |
| 219423_x_at | TNFRSF25 | | TNF receptor superfamily member 25 | | 3.15E-02 | | 1.108918 |
| 234904_x_at | ELAVL4 | | ELAV like neuron-specific RNA binding protein 4 | | 3.15E-02 | | 1.1898 |
| 210127_at | RAB6B | | RAB6B, member RAS oncogene family | | 3.16E-02 | | 1.022623 |
| 1554440_at | KIAA0513 | | KIAA0513 | | 3.17E-02 | | 1.02201 |
| 229459_at | FAM19A5 | | family with sequence similarity 19 member A5, C-C motif chemokine like | | 3.18E-02 | | 1.089873 |
| 204444_at | KIF11 | | kinesin family member 11 | | 3.20E-02 | | 1.002924 |
| 244048_x_at |  | |  | | 3.20E-02 | | 1.188446 |
| 204380_s_at | FGFR3 | | fibroblast growth factor receptor 3 | | 3.22E-02 | | 1.296174 |
| 1559653_at | GRTP1-AS1 | | GRTP1 antisense RNA 1 | | 3.22E-02 | | 1.259233 |
| 1553627_s_at | EFCAB13 | | EF-hand calcium binding domain 13 | | 3.23E-02 | | 1.189846 |
| 207441_at | SMR3B | | submaxillary gland androgen regulated protein 3B | | 3.24E-02 | | 1.016503 |
| 1554865_at |  | |  | | 3.24E-02 | | 1.309014 |
| 211122_s_at | CXCL11 | | C-X-C motif chemokine ligand 11 | | 3.24E-02 | | 1.119429 |
| 234164_at |  | |  | | 3.26E-02 | | 1.190664 |
| 1557275_a_at | TLCD2 | | TLC domain containing 2 | | 3.32E-02 | | 1.265662 |
| 206891_at | ACTN3 | | actinin alpha 3 (gene/pseudogene) | | 3.34E-02 | | 1.74024 |
| 214110_s_at | LOC654342///LOC645166 | | lymphocyte-specific protein 1 pseudogene///lymphocyte-specific protein 1 pseudogene | | 3.34E-02 | | 1.121484 |
| 221405_at |  | |  | | 3.36E-02 | | 1.008066 |
| 239272_at | MMP28 | | matrix metallopeptidase 28 | | 3.38E-02 | | 1.10317 |
| 1554409_at | RCC1L | | RCC1 like | | 3.38E-02 | | 1.000734 |
| 1564220_a_at | LINC01234 | | long intergenic non-protein coding RNA 1234 | | 3.38E-02 | | 1.170848 |
| 231760_at | LINC00029 | | long intergenic non-protein coding RNA 29 | | 3.41E-02 | | 1.231547 |
| 1556541_s_at |  | |  | | 3.42E-02 | | 1.224949 |
| 211039_at | CHRNA1 | | cholinergic receptor nicotinic alpha 1 subunit | | 3.43E-02 | | 1.001171 |
| 1558957_s_at | CEPT1 | | choline/ethanolamine phosphotransferase 1 | | 3.45E-02 | | 1.313691 |
| 216476_at |  | |  | | 3.46E-02 | | 1.114117 |
| 1553706_at | HTRA4 | | HtrA serine peptidase 4 | | 3.46E-02 | | 1.246807 |
| 241198_s_at | C11orf70 | | chromosome 11 open reading frame 70 | | 3.49E-02 | | 1.114327 |
| 231711_at | ACPP | | acid phosphatase, prostate | | 3.49E-02 | | 1.136154 |
| 244736_at |  | |  | | 3.50E-02 | | 1.142998 |
| 1562595_at | LOC101928314 | | uncharacterized LOC101928314 | | 3.51E-02 | | 1.193482 |
| 214554_at | HIST1H2AL | | histone cluster 1, H2al | | 3.52E-02 | | 1.104278 |
| 226570_at | ATP1B3 | | ATPase Na+/K+ transporting subunit beta 3 | | 3.54E-02 | | 1.033877 |
| 243758_at | CFAP100 | | cilia and flagella associated protein 100 | | 3.54E-02 | | 1.062745 |
| 235650_at | CDHR3 | | cadherin related family member 3 | | 3.54E-02 | | 1.113207 |
| 207031_at | NKX3-2 | | NK3 homeobox 2 | | 3.54E-02 | | 1.000393 |
| 1552912_a_at | IL23R | | interleukin 23 receptor | | 3.57E-02 | | 1.093408 |
| 1561025_at | CDKAL1 | | CDK5 regulatory subunit associated protein 1 like 1 | | 3.57E-02 | | 1.125778 |
| 237281_at | AKAP14 | | A-kinase anchoring protein 14 | | 3.57E-02 | | 1.035124 |
| 205951_at | MYH1 | | myosin heavy chain 1 | | 3.58E-02 | | 1.031316 |
| 230190_at | NDFIP2 | | Nedd4 family interacting protein 2 | | 3.58E-02 | | 1.028297 |
| 241023_at |  | |  | | 3.59E-02 | | 1.278146 |
| 233355_at | LINC00176 | | long intergenic non-protein coding RNA 176 | | 3.61E-02 | | 1.090979 |
| 231668_x_at | IGHG1 | | immunoglobulin heavy constant gamma 1 (G1m marker) | | 3.62E-02 | | 1.053356 |
| 244441_at | USP31 | | ubiquitin specific peptidase 31 | | 3.62E-02 | | 1.056514 |
| 209460_at | ABAT | | 4-aminobutyrate aminotransferase | | 3.64E-02 | | 1.081908 |
| 238889_at | AGBL5 | | ATP/GTP binding protein like 5 | | 3.66E-02 | | 1.04996 |
| 237435_at |  | |  | | 3.66E-02 | | 1.365772 |
| 207334_s_at | TGFBR2 | | transforming growth factor beta receptor 2 | | 3.68E-02 | | 1.007531 |
| 237296_at |  | |  | | 3.69E-02 | | 1.058364 |
| 1558846_at | PNLIPRP3 | | pancreatic lipase related protein 3 | | 3.70E-02 | | 1.078951 |
| 208579_x_at | H2BFS | | H2B histone family member S | | 3.72E-02 | | 1.005789 |
| 244513_at | TMEM161B | | transmembrane protein 161B | | 3.73E-02 | | 1.014293 |
| 237205_at | LINC00238 | | long intergenic non-protein coding RNA 238 | | 3.73E-02 | | 1.30174 |
| 205081_at | CRIP1 | | cysteine rich protein 1 | | 3.74E-02 | | 1.072887 |
| 238439_at | ANKRD22 | | ankyrin repeat domain 22 | | 3.76E-02 | | 1.030148 |
| 228792_at | MYEF2 | | myelin expression factor 2 | | 3.77E-02 | | 1.013519 |
| 204999_s_at | ATF5 | | activating transcription factor 5 | | 3.78E-02 | | 1.044082 |
| 1562107_at | LOC101928833 | | uncharacterized LOC101928833 | | 3.78E-02 | | 1.071258 |
| 219152_at | PODXL2 | | podocalyxin like 2 | | 3.79E-02 | | 1.025393 |
| 243871_at | LOC100130476 | | uncharacterized LOC100130476 | | 3.80E-02 | | 1.032288 |
| 244350_at | MYO10 | | myosin X | | 3.80E-02 | | 1.008074 |
| 207299_s_at | GRM1 | | glutamate metabotropic receptor 1 | | 3.81E-02 | | 1.099851 |
| 211766_s_at | PNLIPRP2 | | pancreatic lipase related protein 2 (gene/pseudogene) | | 3.81E-02 | | 1.032893 |
| 236608_at | ADGRF3 | | adhesion G protein-coupled receptor F3 | | 3.82E-02 | | 1.165926 |
| 220810_at | CLCA3P | | chloride channel accessory 3, pseudogene | | 3.83E-02 | | 1.070257 |
| 1559655_at | MAMDC2-AS1 | | MAMDC2 antisense RNA 1 | | 3.83E-02 | | 1.011182 |
| 1558971_at | THEMIS | | thymocyte selection associated | | 3.85E-02 | | 1.088357 |
| 1562234_a_at | NAV3 | | neuron navigator 3 | | 3.86E-02 | | 1.069591 |
| 1562973_at | LOC101928201 | | uncharacterized LOC101928201 | | 3.87E-02 | | 1.041695 |
| 231309_at |  | |  | | 3.87E-02 | | 1.088473 |
| 1559842_at |  | |  | | 3.89E-02 | | 1.256689 |
| 205314_x_at | SNTB2 | | syntrophin beta 2 | | 3.89E-02 | | 1.049739 |
| 1555689_at | CD80 | | CD80 molecule | | 3.94E-02 | | 1.178423 |
| 1556350_a_at | EIF4A2 | | eukaryotic translation initiation factor 4A2 | | 3.94E-02 | | 1.00574 |
| 1563920_at | FAM45A | | family with sequence similarity 45 member A | | 3.95E-02 | | 1.134801 |
| 243193_at |  | |  | | 3.97E-02 | | 1.018992 |
| 1562272_at | LOC101927914 | | uncharacterized LOC101927914 | | 3.97E-02 | | 1.045129 |
| 239680_at | WDR76 | | WD repeat domain 76 | | 4.02E-02 | | 1.196786 |
| 210498_at | CLTC | | clathrin heavy chain | | 4.03E-02 | | 1.114944 |
| 1565107_x_at | |  | | 4.05E-02 | | 1.158964 | |
| 239609_s_at | LPCAT4 | | lysophosphatidylcholine acyltransferase 4 | | 4.05E-02 | | 1.107149 |
| 237126_at |  | |  | | 4.09E-02 | | 1.022274 |
| 1558944_at | LOC100507353///CACNA1A | | uncharacterized LOC100507353///calcium voltage-gated channel subunit alpha1 A | | 4.09E-02 | | 1.118173 |
| 1559403_at | HMGN3-AS1 | | HMGN3 antisense RNA 1 | | 4.09E-02 | | 1.033947 |
| 224965_at | GNG2 | | G protein subunit gamma 2 | | 4.14E-02 | | 1.122313 |
| 215437_x_at | BAZ2A | | bromodomain adjacent to zinc finger domain 2A | | 4.20E-02 | | 1.003532 |
| 205888_s_at | JAKMIP2 | | janus kinase and microtubule interacting protein 2 | | 4.20E-02 | | 1.025547 |
| 237233_at |  | |  | | 4.21E-02 | | 1.151021 |
| 222202_at |  | |  | | 4.24E-02 | | 1.035264 |
| 1556445_at |  | |  | | 4.25E-02 | | 1.009622 |
| 243197_at |  | |  | | 4.31E-02 | | 1.089495 |
| 242908_x_at |  | |  | | 4.32E-02 | | 1.197425 |
| 235700_at | CT45A8///CT45A9///CT45A10///CT45A7///CT45A2///CT45A1///CT45A6///CT45A5///CT45A3 | | cancer/testis antigen family 45, member A8///cancer/testis antigen family 45, member A9///cancer/testis antigen family 45, member A10///cancer/testis antigen family 45, member A7///cancer/testis antigen family 45, member A2///cancer/testis antigen family 45, member A1///cancer/testis antigen family 45, member A6///cancer/testis antigen family 45, member A5///cancer/testis antigen family 45, member A3 | | 4.34E-02 | | 1.127019 |
| 207080_s_at | PYY | | peptide YY | | 4.35E-02 | | 1.026825 |
| 1564794_at |  | |  | | 4.35E-02 | | 1.082615 |
| 232830_at | RNF32 | | ring finger protein 32 | | 4.37E-02 | | 1.008134 |
| 244849_at | SEMA3A | | semaphorin 3A | | 4.40E-02 | | 1.156075 |
| 231417_at |  | |  | | 4.41E-02 | | 1.084449 |
| 1559288_at |  | |  | | 4.41E-02 | | 1.340365 |
| 232956_at |  | |  | | 4.43E-02 | | 1.018303 |
| 210834_s_at | PTGER3 | | prostaglandin E receptor 3 | | 4.45E-02 | | 1.069064 |
| 216966_at | ITGA2B | | integrin subunit alpha 2b | | 4.47E-02 | | 1.075788 |
| 215752_at | SIK2 | | salt inducible kinase 2 | | 4.47E-02 | | 1.006081 |
| 1554828_at | PDGFRA | | platelet derived growth factor receptor alpha | | 4.47E-02 | | 1.080041 |
| 1561596_at |  | |  | | 4.48E-02 | | 1.069362 |
| 242084_at | THEG5 | | testis highly expressed protein 5 | | 4.49E-02 | | 1.004776 |
| 241207_at |  | |  | | 4.49E-02 | | 1.059074 |
| 210631_at | NF1P9///NF1 | | neurofibromin 1 pseudogene 9///neurofibromin 1 | | 4.51E-02 | | 1.07578 |
| 1553126_a_at | SLC39A12 | | solute carrier family 39 member 12 | | 4.54E-02 | | 1.082514 |
| 1561274_at | CRYBB2P1 | | crystallin beta B2 pseudogene 1 | | 4.54E-02 | | 1.294229 |
| 1553409_at | ADAMTS20 | | ADAM metallopeptidase with thrombospondin type 1 motif 20 | | 4.55E-02 | | 1.032582 |
| 1560912_at | LINC01120 | | long intergenic non-protein coding RNA 1120 | | 4.56E-02 | | 1.084788 |
| 1559725_at | LINC01537 | | long intergenic non-protein coding RNA 1537 | | 4.58E-02 | | 1.013458 |
| 215231_at | PRKAG2 | | protein kinase AMP-activated non-catalytic subunit gamma 2 | | 4.58E-02 | | 1.048393 |
| 244173_at | MIS18BP1 | | MIS18 binding protein 1 | | 4.58E-02 | | 1.113483 |
| 244812_at |  | |  | | 4.62E-02 | | 1.021933 |
| 216027_at | TMX4 | | thioredoxin related transmembrane protein 4 | | 4.63E-02 | | 1.118794 |
| 1556461_at |  | |  | | 4.65E-02 | | 1.256463 |
| 1558247_s_at | NTN5 | | netrin 5 | | 4.70E-02 | | 1.068683 |
| 1563083_s_at | LINC00486 | | long intergenic non-protein coding RNA 486 | | 4.72E-02 | | 1.129173 |
| 230423_at | FOXD3-AS1 | | FOXD3 antisense RNA 1 (head to head) | | 4.77E-02 | | 1.016791 |
| 238392_at |  | |  | | 4.78E-02 | | 1.082067 |
| 228979_at | SFTA3 | | surfactant associated 3 | | 4.80E-02 | | 1.182003 |
| 1552554_a_at | NLRC4 | | NLR family CARD domain containing 4 | | 4.80E-02 | | 1.027651 |
| 224022_x_at | WNT16 | | Wnt family member 16 | | 4.81E-02 | | 1.279209 |
| 1559688_at | GRAPL | | GRB2 related adaptor protein like | | 4.81E-02 | | 1.26799 |
| 205764_at | CSNK1A1 | | casein kinase 1 alpha 1 | | 4.82E-02 | | 1.042592 |
| 222886_at | NSUN3 | | NOP2/Sun RNA methyltransferase family member 3 | | 4.84E-02 | | 1.014965 |
| 236157_at | RBM14-RBM4///RBM4 | | RBM14-RBM4 readthrough///RNA binding motif protein 4 | | 4.85E-02 | | 1.051048 |
| 207640_x_at | NTN3 | | netrin 3 | | 4.87E-02 | | 1.011369 |
| 241979_x_at |  | |  | | 4.92E-02 | | 1.262376 |
