# Supplementary material for: Identification of the hub susceptibility genes and related common transcription factors in the skeletal muscle of Type 2 Diabetes Mellitus
Source: BMC Endocr Disord. 2022 Nov 11;22:276. doi: 10.1186/s12902-022-01195-0 (PMC9652898; doi:10.1186/s12902-022-01195-0)
Supplement: Supplementary file 2 — Additional file 2: Table S2. [file 12902_2022_1195_MOESM2_ESM.docx]

**Table S2. 767 DEGs between T2DM subjects and the normoglycemic IR subjects without PFH of T2DM (non PFH vs T2DM)**

| **ID** | **Gene.symbol** | **Gene.title** | **P.Value** | **logFC** |
| --- | --- | --- | --- | --- |
| 1552731_at | ABRA | actin binding Rho activating protein | 1.71E-06 | 1.365671 |
| 1552732_at | ABRA | actin binding Rho activating protein | 1.86E-05 | 1.162363 |
| 202149_at | NEDD9 | neural precursor cell expressed, developmentally down-regulated 9 | 3.25E-05 | 1.009585 |
| 1561361_at | ZNF660 | zinc finger protein 660 | 3.45E-05 | 1.551033 |
| 223973_at | MIR7-3HG | MIR7-3 host gene | 5.5E-05 | 1.932639 |
| 207384_at | PGLYRP1 | peptidoglycan recognition protein 1 | 7.38E-05 | 1.05081 |
| 1563070_at | LINC01395 | long intergenic non-protein coding RNA 1395 | 8.97E-05 | 1.737242 |
| 211062_s_at | GPR78///CPZ | G protein-coupled receptor 78///carboxypeptidase Z | 9.78E-05 | 1.830561 |
| 1553347_s_at | KCNA6 | potassium voltage-gated channel subfamily A member 6 | 0.000109 | 1.272036 |
| 207800_at | AKAP5 | A-kinase anchoring protein 5 | 0.000124 | 1.403669 |
| 222848_at | CENPK | centromere protein K | 0.000133 | 2.208237 |
| 244572_at | KY | kyphoscoliosis peptidase | 0.000173 | -1.04049 |
| 206799_at | SCGB1D2 | secretoglobin family 1D member 2 | 0.000181 | -2.17596 |
| 207914_x_at | EVX1 | even-skipped homeobox 1 | 0.000191 | 1.147837 |
| 236859_at | RUNX2 | runt related transcription factor 2 | 0.000204 | 1.353136 |
| 215176_x_at | IGK///IGKC | immunoglobulin kappa locus///immunoglobulin kappa constant | 0.000216 | 1.356961 |
| 230458_at | SLC45A1 | solute carrier family 45 member 1 | 0.000223 | 1.348776 |
| 237828_at | SRRM4 | serine/arginine repetitive matrix 4 | 0.000243 | 1.502145 |
| 238491_at | TAF1A-AS1 | TAF1A antisense RNA 1 | 0.000245 | 1.331896 |
| 238439_at | ANKRD22 | ankyrin repeat domain 22 | 0.000298 | 1.9419 |
| 243410_at | PTPN2 | protein tyrosine phosphatase, non-receptor type 2 | 0.0003 | -1.12312 |
| 224169_at | NPFFR2 | neuropeptide FF receptor 2 | 0.00031 | -1.29257 |
| 227067_x_at | LOC101929796///LOC100996763///LOC100996717///NOTCH2NL///NOTCH2 | notch homolog 2 N-terminal-like protein///notch homolog 2 N-terminal-like protein///notch homolog 2 N-terminal-like protein///notch 2 N-terminal like///notch 2 | 0.000313 | 1.728067 |
| 209081_s_at | COL18A1 | collagen type XVIII alpha 1 chain | 0.000324 | 1.514119 |
| 202833_s_at | SERPINA1 | serpin family A member 1 | 0.000328 | 1.669157 |
| 214296_x_at | IZUMO4 | IZUMO family member 4 | 0.000338 | 1.438645 |
| 201867_s_at | TBL1X | transducin (beta)-like 1X-linked | 0.000345 | -2.23862 |
| 206039_at | RAB33A | RAB33A, member RAS oncogene family | 0.000436 | 1.493508 |
| 230662_at | RNF187 | ring finger protein 187 | 0.000442 | 1.660666 |
| 1560412_at | LOC100507506 | uncharacterized LOC100507506 | 0.000478 | 1.92775 |
| 238695_s_at | RAB39B | RAB39B, member RAS oncogene family | 0.000516 | 2.20087 |
| 212225_at | EIF1 | eukaryotic translation initiation factor 1 | 0.000522 | -1.12098 |
| 233297_s_at | CCDC169-SOHLH2///CCDC169 | CCDC169-SOHLH2 readthrough///coiled-coil domain containing 169 | 0.000543 | 1.950085 |
| 231256_at | LOC727944 | uncharacterized LOC727944 | 0.000615 | 1.364507 |
| 233742_at | METTL22 | methyltransferase like 22 | 0.000633 | -1.52612 |
| 235291_s_at | FLJ32255 | uncharacterized LOC643977 | 0.000665 | 1.327145 |
| 222234_s_at | DBNDD1 | dysbindin domain containing 1 | 0.000667 | 1.197909 |
| 222764_at | ASRGL1 | asparaginase like 1 | 0.000734 | 1.137891 |
| 1564402_at | LOC146795 | uncharacterized LOC146795 | 0.000769 | -1.01365 |
| 1561098_at | LINC00616 | long intergenic non-protein coding RNA 616 | 0.000777 | 1.5704 |
| 221583_s_at | KCNMA1 | potassium calcium-activated channel subfamily M alpha 1 | 0.000814 | -1.09888 |
| 1553298_at | C17orf77 | chromosome 17 open reading frame 77 | 0.000844 | 1.802309 |
| 1557483_at | LOC284788 | uncharacterized LOC284788 | 0.000875 | 1.495295 |
| 243721_at | C18orf61 | uncharacterized LOC497259 | 0.000876 | -1.06921 |
| 209116_x_at | HBB | hemoglobin subunit beta | 0.000917 | 1.009028 |
| 211217_s_at | KCNQ1 | potassium voltage-gated channel subfamily Q member 1 | 0.000943 | 1.479555 |
| 1562364_at | GVINP1 | GTPase, very large interferon inducible pseudogene 1 | 0.000944 | 1.958656 |
| 211674_x_at | CTAG1A///CTAG1B | cancer/testis antigen 1A///cancer/testis antigen 1B | 0.000956 | 1.627535 |
| 231035_s_at | OTUD1 | OTU deubiquitinase 1 | 0.001027 | 1.130638 |
| 219386_s_at | SLAMF8 | SLAM family member 8 | 0.001033 | 1.793307 |
| 1562902_at | LOC101927723 | uncharacterized LOC101927723 | 0.001033 | 1.517904 |
| 226678_at | UNC13D | unc-13 homolog D | 0.001117 | 1.14072 |
| 233436_at | MTBP | MDM2 binding protein | 0.001134 | 1.316411 |
| 1563595_at | SRGAP3 | SLIT-ROBO Rho GTPase activating protein 3 | 0.001146 | 1.41956 |
| 215052_at | FRMPD4 | FERM and PDZ domain containing 4 | 0.001184 | 1.414431 |
| 236312_at | MAD2L1 | MAD2 mitotic arrest deficient-like 1 (yeast) | 0.001188 | 1.730099 |
| 217495_x_at | CALCA | calcitonin related polypeptide alpha | 0.001213 | 1.156096 |
| 217411_s_at | RREB1 | ras responsive element binding protein 1 | 0.001259 | 1.109348 |
| 1554744_at | CARD16 | caspase recruitment domain family member 16 | 0.001302 | 1.286475 |
| 236499_at | FAAP20 | Fanconi anemia core complex associated protein 20 | 0.001316 | 1.148252 |
| 1552289_a_at | CILP2 | cartilage intermediate layer protein 2 | 0.001346 | -1.20261 |
| 1555569_a_at | KCTD7///RABGEF1 | potassium channel tetramerization domain containing 7///RAB guanine nucleotide exchange factor 1 | 0.001362 | 1.02487 |
| 1552402_at | CALML6 | calmodulin like 6 | 0.001379 | 1.664936 |
| 1570204_at | ZBED3-AS1 | ZBED3 antisense RNA 1 | 0.001388 | 1.824577 |
| 203645_s_at | CD163 | CD163 molecule | 0.00145 | 1.790973 |
| 218300_at | PAGR1 | PAXIP1 associated glutamate rich protein 1 | 0.001467 | 1.133479 |
| 244849_at | SEMA3A | semaphorin 3A | 0.001541 | 1.745727 |
| 222926_at | DCDC2 | doublecortin domain containing 2 | 0.001543 | 2.044274 |
| 1568286_at | HMGA2 | high mobility group AT-hook 2 | 0.001563 | 1.86328 |
| 230989_s_at | TSSK6 | testis specific serine kinase 6 | 0.001579 | 1.1404 |
| 209794_at | SRGAP3 | SLIT-ROBO Rho GTPase activating protein 3 | 0.001602 | 1.190553 |
| 204105_s_at | NRCAM | neuronal cell adhesion molecule | 0.001621 | -1.15012 |
| 1560069_at | PLEKHM3 | pleckstrin homology domain containing M3 | 0.001629 | -1.30971 |
| 240288_at | KCNRG///TRIM13 | potassium channel regulator///tripartite motif containing 13 | 0.00165 | -1.68135 |
| 207149_at | CDH12 | cadherin 12 | 0.001672 | 1.711702 |
| 219429_at | FA2H | fatty acid 2-hydroxylase | 0.001685 | 1.718929 |
| 227355_at | RBM26 | RNA binding motif protein 26 | 0.001691 | 1.291655 |
| 206803_at | PDYN | prodynorphin | 0.001693 | 1.292236 |
| 233153_at | LOC101930081 | uncharacterized LOC101930081 | 0.001697 | -1.60453 |
| 237504_at | INTS10 | integrator complex subunit 10 | 0.001725 | -1.15145 |
| 226140_s_at | OTUD1 | OTU deubiquitinase 1 | 0.001726 | 1.310178 |
| 1554594_at | ARHGAP27 | Rho GTPase activating protein 27 | 0.001743 | 1.194639 |
| 1553917_at | PIKFYVE | phosphoinositide kinase, FYVE-type zinc finger containing | 0.001746 | 1.416555 |
| 213975_s_at | LYZ | lysozyme | 0.001782 | 1.429563 |
| 209652_s_at | PGF | placental growth factor | 0.001801 | -1.17388 |
| 216632_at | NAV3 | neuron navigator 3 | 0.001834 | 1.120521 |
| 236688_at | FRMPD3 | FERM and PDZ domain containing 3 | 0.001855 | 1.114292 |
| 211745_x_at | HBA2///HBA1 | hemoglobin subunit alpha 2///hemoglobin subunit alpha 1 | 0.001875 | 1.145183 |
| 239178_at | FGF9 | fibroblast growth factor 9 | 0.001891 | -2.15964 |
| 216452_at | TRPM3 | transient receptor potential cation channel subfamily M member 3 | 0.001897 | 1.226148 |
| 223665_at | ACTRT3 | actin related protein T3 | 0.001936 | -1.07265 |
| 241934_at | NTM | neurotrimin | 0.001953 | 1.311401 |
| 1561347_a_at | LOC101927166 | uncharacterized LOC101927166 | 0.00196 | 1.521784 |
| 216925_s_at | TAL1 | TAL bHLH transcription factor 1, erythroid differentiation factor | 0.001981 | 1.460037 |
| 1563542_a_at | SCML4 | sex comb on midleg-like 4 (Drosophila) | 0.001983 | 1.508663 |
| 232423_at | ARSD | arylsulfatase D | 0.002006 | 1.30665 |
| 1557063_at | DICER1-AS1 | DICER1 antisense RNA 1 | 0.002079 | 1.449758 |
| 239027_at | DOCK8 | dedicator of cytokinesis 8 | 0.00209 | 1.831353 |
| 1568633_a_at | LOC101927809 | uncharacterized LOC101927809 | 0.002096 | 1.678389 |
| 221081_s_at | DENND2D | DENN domain containing 2D | 0.002108 | 1.401756 |
| 205038_at | IKZF1 | IKAROS family zinc finger 1 | 0.002139 | 1.603808 |
| 222183_x_at | MBD3 | methyl-CpG binding domain protein 3 | 0.002143 | 1.027932 |
| 216026_s_at | POLE | DNA polymerase epsilon, catalytic subunit | 0.00229 | 1.167574 |
| 1562022_s_at | LOC100130987///RAD9A | uncharacterized LOC100130987///RAD9 checkpoint clamp component A | 0.002303 | 1.313323 |
| 241819_at | TNFSF8 | tumor necrosis factor superfamily member 8 | 0.002306 | 1.193659 |
| 226267_at | JDP2 | Jun dimerization protein 2 | 0.002314 | 1.257679 |
| 202340_x_at | NR4A1 | nuclear receptor subfamily 4 group A member 1 | 0.002379 | 1.209538 |
| 220156_at | EFCAB1 | EF-hand calcium binding domain 1 | 0.00238 | 1.447539 |
| 231159_at | CXorf51B///CXorf51A | chromosome X open reading frame 51B///chromosome X open reading frame 51A | 0.002389 | 1.274072 |
| 239500_at | EFCAB1 | EF-hand calcium binding domain 1 | 0.002426 | -1.33597 |
| 1561443_at | LOC101928535 | uncharacterized LOC101928535 | 0.002539 | 1.178438 |
| 220315_at | PARP11 | poly(ADP-ribose) polymerase family member 11 | 0.002581 | 1.108662 |
| 232627_at | HGS | hepatocyte growth factor-regulated tyrosine kinase substrate | 0.002601 | 1.05056 |
| 217414_x_at | HBA2///HBA1 | hemoglobin subunit alpha 2///hemoglobin subunit alpha 1 | 0.002642 | 1.33754 |
| 229783_at | AKAP13 | A-kinase anchoring protein 13 | 0.002648 | 1.264281 |
| 208034_s_at | PROZ | protein Z, vitamin K dependent plasma glycoprotein | 0.002752 | 1.619031 |
| 224229_s_at | AKT3 | AKT serine/threonine kinase 3 | 0.002759 | -1.37626 |
| 210895_s_at | CD86 | CD86 molecule | 0.002772 | 1.422437 |
| 232079_s_at | NECTIN2 | nectin cell adhesion molecule 2 | 0.002775 | 1.345432 |
| 232329_at | RANBP10 | RAN binding protein 10 | 0.002819 | -1.19971 |
| 233892_at | GRIN3B | glutamate ionotropic receptor NMDA type subunit 3B | 0.002872 | 1.491354 |
| 218704_at | RNF43 | ring finger protein 43 | 0.002894 | -1.18764 |
| 219059_s_at | LYVE1 | lymphatic vessel endothelial hyaluronan receptor 1 | 0.002899 | 1.216966 |
| 222632_s_at | LZTFL1 | leucine zipper transcription factor like 1 | 0.002956 | -1.35367 |
| 1554296_at | CYP19A1 | cytochrome P450 family 19 subfamily A member 1 | 0.002983 | 1.792584 |
| 240192_at | GATA3-AS1 | GATA3 antisense RNA 1 | 0.003003 | 1.381948 |
| 221872_at | RARRES1 | retinoic acid receptor responder 1 | 0.003022 | -1.14103 |
| 204018_x_at | HBA2///HBA1 | hemoglobin subunit alpha 2///hemoglobin subunit alpha 1 | 0.003023 | 1.232945 |
| 242502_at | KCNH5 | potassium voltage-gated channel subfamily H member 5 | 0.003076 | 1.375674 |
| 211699_x_at | HBA2///HBA1 | hemoglobin subunit alpha 2///hemoglobin subunit alpha 1 | 0.003114 | 1.269972 |
| 241047_at | CASC15 | cancer susceptibility candidate 15 (non-protein coding) | 0.003131 | 1.356033 |
| 1554280_a_at | C9orf43 | chromosome 9 open reading frame 43 | 0.003152 | 1.256626 |
| 221671_x_at | IGK///IGKC | immunoglobulin kappa locus///immunoglobulin kappa constant | 0.003162 | 1.258886 |
| 210765_at | CSE1L | chromosome segregation 1 like | 0.003197 | 1.006184 |
| 1562776_at | LOC339807 | uncharacterized LOC339807 | 0.003245 | 1.294327 |
| 235180_at | STYX | serine/threonine/tyrosine interacting protein | 0.00325 | -1.6079 |
| 238889_at | AGBL5 | ATP/GTP binding protein like 5 | 0.003288 | 1.352352 |
| 227015_at | ASPHD2 | aspartate beta-hydroxylase domain containing 2 | 0.003339 | -1.55329 |
| 222886_at | NSUN3 | NOP2/Sun RNA methyltransferase family member 3 | 0.00342 | 1.370329 |
| 1558463_s_at | LMF1 | lipase maturation factor 1 | 0.003426 | 1.031428 |
| 233680_at | LOC101929454 | uncharacterized LOC101929454 | 0.003427 | -1.27253 |
| 241966_at | MYO5A | myosin VA | 0.003491 | 1.230577 |
| 209458_x_at | HBA2///HBA1 | hemoglobin subunit alpha 2///hemoglobin subunit alpha 1 | 0.003562 | 1.202256 |
| 220561_at | IGF2-AS | IGF2 antisense RNA | 0.00358 | 1.03393 |
| 215028_at | SEMA6A | semaphorin 6A | 0.003626 | -1.00043 |
| 1565728_at | TTC34///LOC284630 | tetratricopeptide repeat domain 34///uncharacterized LOC284630 | 0.00365 | 1.372024 |
| 220626_at | SERPINA10 | serpin family A member 10 | 0.003652 | 1.538891 |
| 223977_s_at | LINC00470 | long intergenic non-protein coding RNA 470 | 0.003672 | 2.129174 |
| 220845_at | ACOXL | acyl-CoA oxidase-like | 0.003681 | 1.4447 |
| 1553318_at | RIBC1 | RIB43A domain with coiled-coils 1 | 0.003689 | 1.58329 |
| 206919_at | ELK4 | ELK4, ETS transcription factor | 0.003794 | 1.248735 |
| 1555195_at | FBXO36 | F-box protein 36 | 0.003819 | 1.623561 |
| 234195_at | TNFRSF10C | TNF receptor superfamily member 10c | 0.003907 | 1.026258 |
| 1555325_s_at | ZNF26 | zinc finger protein 26 | 0.003961 | -1.81997 |
| 1569306_at | LOC105371622 | uncharacterized LOC105371622 | 0.004 | 1.459137 |
| 243776_at | LOC105374428 | uncharacterized LOC105374428 | 0.004061 | -1.80437 |
| 235704_at | LOC102723983///DAZAP2 | uncharacterized LOC102723983///DAZ associated protein 2 | 0.004075 | 1.269634 |
| 241117_at | LOXHD1 | lipoxygenase homology domains 1 | 0.00416 | 1.294641 |
| 205481_at | ADORA1 | adenosine A1 receptor | 0.004176 | 1.038206 |
| 224310_s_at | BCL11B | B-cell CLL/lymphoma 11B | 0.004207 | 1.353318 |
| 219434_at | TREM1 | triggering receptor expressed on myeloid cells 1 | 0.004266 | 1.110264 |
| 1565858_at | SNORA71A | small nucleolar RNA, H/ACA box 71A | 0.004291 | 1.185413 |
| 205960_at | PDK4 | pyruvate dehydrogenase kinase 4 | 0.004376 | 1.521894 |
| 232624_at | ABTB2 | ankyrin repeat and BTB domain containing 2 | 0.004395 | 1.011021 |
| 233895_at | ANKRD24 | ankyrin repeat domain 24 | 0.004425 | -1.48401 |
| 243301_at | COL22A1 | collagen type XXII alpha 1 chain | 0.00446 | 1.610703 |
| 1560493_a_at | CPXCR1 | CPX chromosome region, candidate 1 | 0.004475 | 1.042609 |
| 215097_at | CAPZB | capping actin protein of muscle Z-line beta subunit | 0.004511 | 1.000828 |
| 228144_at | ZNF300 | zinc finger protein 300 | 0.004545 | -1.05346 |
| 1569954_at | LOC101928834 | uncharacterized LOC101928834 | 0.004547 | 1.344507 |
| 1557998_at | NAALADL2 | N-acetylated alpha-linked acidic dipeptidase-like 2 | 0.004562 | 1.560828 |
| 208525_s_at | OR2F2///OR2F1 | olfactory receptor family 2 subfamily F member 2///olfactory receptor family 2 subfamily F member 1 (gene/pseudogene) | 0.004567 | -1.62251 |
| 237162_at | KANK1 | KN motif and ankyrin repeat domains 1 | 0.004577 | 1.229278 |
| 223472_at | WHSC1 | Wolf-Hirschhorn syndrome candidate 1 | 0.004592 | 1.45025 |
| 211602_s_at | TRPC1 | transient receptor potential cation channel subfamily C member 1 | 0.004606 | 1.625557 |
| 1561940_at | LOC100128843///CHFR | uncharacterized LOC100128843///checkpoint with forkhead and ring finger domains, E3 ubiquitin protein ligase | 0.004609 | 1.144512 |
| 236257_at | CD2AP | CD2 associated protein | 0.004689 | -1.0962 |
| 1557991_at | METTL6 | methyltransferase like 6 | 0.004701 | 1.329456 |
| 211736_at | SP2 | Sp2 transcription factor | 0.004714 | -1.38794 |
| 1553829_at | CYP1B1-AS1 | CYP1B1 antisense RNA 1 | 0.00474 | 1.326956 |
| 1559266_s_at | SKIDA1 | SKI/DACH domain containing 1 | 0.004745 | 1.196289 |
| 205190_at | PLS1 | plastin 1 | 0.004753 | -1.35779 |
| 232734_at | TTC23 | tetratricopeptide repeat domain 23 | 0.004775 | 1.291959 |
| 211579_at | ITGB3 | integrin subunit beta 3 | 0.004802 | 1.248571 |
| 234005_x_at | STK36 | serine/threonine kinase 36 | 0.004829 | 1.082695 |
| 1554380_at | NEK11 | NIMA related kinase 11 | 0.004839 | 1.493643 |
| 1557719_at | PIKFYVE | phosphoinositide kinase, FYVE-type zinc finger containing | 0.004977 | 1.366971 |
| 230044_at | PCYT2 | phosphate cytidylyltransferase 2, ethanolamine | 0.00498 | 1.163216 |
| 220026_at | CLCA4 | chloride channel accessory 4 | 0.005005 | 1.336215 |
| 1558102_at | HDGFRP3 | hepatoma-derived growth factor, related protein 3 | 0.00502 | 1.156949 |
| 224211_at | FOXP3 | forkhead box P3 | 0.005036 | 1.374521 |
| 240572_s_at | LOC374443 | C-type lectin domain family 2 member D pseudogene | 0.005052 | 1.685567 |
| 207262_at | APOF | apolipoprotein F | 0.005089 | -1.48812 |
| 220709_at | ZNF556 | zinc finger protein 556 | 0.005095 | -1.11877 |
| 214326_x_at | JUND | JunD proto-oncogene, AP-1 transcription factor subunit | 0.005135 | 1.678296 |
| 216935_at | LINC00302 | long intergenic non-protein coding RNA 302 | 0.005185 | 1.537042 |
| 206071_s_at | EPHA3 | EPH receptor A3 | 0.005267 | 1.030483 |
| 238016_s_at | LOC100507507 | uncharacterized LOC100507507 | 0.005316 | 1.437319 |
| 1554190_s_at | PLEKHS1 | pleckstrin homology domain containing S1 | 0.005326 | 1.626063 |
| 219612_s_at | FGG | fibrinogen gamma chain | 0.005338 | -1.25225 |
| 219309_at | C22orf46 | chromosome 22 open reading frame 46 | 0.005349 | 1.099128 |
| 209690_s_at | DOK4 | docking protein 4 | 0.005352 | 1.106988 |
| 219987_at | ERVMER34-1 | endogenous retrovirus group MER34 member 1 | 0.005396 | -1.04938 |
| 204848_x_at | HBG2///HBG1 | hemoglobin subunit gamma 2///hemoglobin subunit gamma 1 | 0.005398 | 1.115048 |
| 240579_at | NBAS | neuroblastoma amplified sequence | 0.005403 | -1.61028 |
| 207527_at | KCNJ9 | potassium voltage-gated channel subfamily J member 9 | 0.005425 | 1.414454 |
| 209982_s_at | NRXN2 | neurexin 2 | 0.00547 | 1.392342 |
| 230393_at | CUL5 | cullin 5 | 0.005521 | -1.11437 |
| 1557558_s_at | MATN1-AS1 | MATN1 antisense RNA 1 | 0.005526 | 1.392723 |
| 241267_at | EHD3 | EH domain containing 3 | 0.005566 | 1.125369 |
| 211834_s_at | TP63 | tumor protein p63 | 0.005622 | 1.315377 |
| 213905_x_at | BGN | biglycan | 0.005653 | 1.541365 |
| 243433_at | ARHGAP30 | Rho GTPase activating protein 30 | 0.005723 | 1.07419 |
| 1558651_at | GVQW2 | GVQW motif containing 2 | 0.005725 | -1.52969 |
| 207130_at | ZMYND8 | zinc finger MYND-type containing 8 | 0.005767 | -1.01487 |
| 202917_s_at | S100A8 | S100 calcium binding protein A8 | 0.005812 | 1.89492 |
| 220316_at | NPAS3 | neuronal PAS domain protein 3 | 0.005917 | 1.01031 |
| 232497_at | ZNF3 | zinc finger protein 3 | 0.005959 | -1.60188 |
| 1559603_at | GPR12 | G protein-coupled receptor 12 | 0.006017 | 1.679125 |
| 233589_x_at | TOR4A | torsin family 4 member A | 0.006084 | 1.218089 |
| 1557016_a_at | LEXM | lymphocyte expansion molecule | 0.006095 | 1.062849 |
| 236823_at | IDS | iduronate 2-sulfatase | 0.006189 | -1.55079 |
| 236396_at | LOC101927263 | uncharacterized LOC101927263 | 0.006197 | 1.094588 |
| 230955_s_at | NOL4L | nucleolar protein 4 like | 0.006245 | 1.074129 |
| 219054_at | NPR3 | natriuretic peptide receptor 3 | 0.006311 | 1.185116 |
| 229440_at | RBM47 | RNA binding motif protein 47 | 0.006365 | 1.2695 |
| 1563255_at | FAM170B-AS1 | FAM170B antisense RNA 1 | 0.006424 | 1.090008 |
| 213556_at | PINLYP | phospholipase A2 inhibitor and LY6/PLAUR domain containing | 0.006434 | 1.205619 |
| 238870_at | KCNK9 | potassium two pore domain channel subfamily K member 9 | 0.006465 | 1.171359 |
| 228892_at | SH3RF2 | SH3 domain containing ring finger 2 | 0.006489 | 1.116819 |
| 224200_s_at | RAD18 | RAD18, E3 ubiquitin protein ligase | 0.006491 | 1.850599 |
| 231661_at | REG3G | regenerating family member 3 gamma | 0.00654 | -1.33851 |
| 210629_x_at | LST1 | leukocyte specific transcript 1 | 0.006666 | 1.143677 |
| 237203_at | LOC101929305 | uncharacterized LOC101929305 | 0.006702 | 1.21861 |
| 241760_x_at | RORA | RAR related orphan receptor A | 0.006914 | 1.893573 |
| 208586_s_at | SSX4B///SSX4 | SSX family member 4B///SSX family member 4 | 0.006933 | 1.85369 |
| 204581_at | CD22 | CD22 molecule | 0.006976 | 1.089836 |
| 244870_at | TES | testin LIM domain protein | 0.007053 | 1.204991 |
| 210823_s_at | PTPRS | protein tyrosine phosphatase, receptor type S | 0.007081 | 1.018087 |
| 1553364_at | PNPLA1 | patatin like phospholipase domain containing 1 | 0.007103 | 1.725347 |
| 206497_at | COA1 | cytochrome c oxidase assembly factor 1 homolog | 0.007116 | -1.06515 |
| 1556609_at | LOC401098 | uncharacterized LOC401098 | 0.00717 | -1.50283 |
| 1555462_at | PPP1R1C | protein phosphatase 1 regulatory inhibitor subunit 1C | 0.007233 | -1.33196 |
| 1560147_at | WDR86-AS1 | WDR86 antisense RNA 1 | 0.007247 | 1.499958 |
| 209622_at | STK16 | serine/threonine kinase 16 | 0.00725 | 1.177415 |
| 223550_s_at | CA10 | carbonic anhydrase 10 | 0.007273 | 1.230501 |
| 210380_s_at | CACNA1G | calcium voltage-gated channel subunit alpha1 G | 0.00731 | 1.413509 |
| 217613_at | TMEM144 | transmembrane protein 144 | 0.007325 | -1.19876 |
| 1569433_at | SAMD5 | sterile alpha motif domain containing 5 | 0.007445 | 1.571718 |
| 234803_at | CSTL1 | cystatin like 1 | 0.007579 | 1.199302 |
| 240036_at | SEC14L1 | SEC14 like lipid binding 1 | 0.007619 | -1.244 |
| 203270_at | DTYMK | deoxythymidylate kinase | 0.007635 | -1.18614 |
| 240838_s_at | DRAIC | downregulated RNA in cancer, inhibitor of cell invasion and migration | 0.007652 | -1.09324 |
| 1557727_at | PCBP1-AS1 | PCBP1 antisense RNA 1 | 0.007696 | 1.318087 |
| 1557864_x_at | PLA2G4E-AS1 | PLA2G4E antisense RNA 1 | 0.007792 | 1.148505 |
| 243334_at | CACNA1D | calcium voltage-gated channel subunit alpha1 D | 0.00789 | 1.369743 |
| 241401_at | WDFY3-AS2 | WDFY3 antisense RNA 2 | 0.007897 | -1.08645 |
| 244790_at | MTCP1 | mature T-cell proliferation 1 | 0.007906 | 1.14491 |
| 216904_at | COL6A1 | collagen type VI alpha 1 chain | 0.007995 | -1.2583 |
| 234477_at | IGHV4-31///IGHA1 | immunoglobulin heavy variable 4-31///immunoglobulin heavy constant alpha 1 | 0.008048 | 1.345609 |
| 221945_at | LOC101927826///FBXO41 | uncharacterized LOC101927826///F-box protein 41 | 0.008065 | 1.425206 |
| 213138_at | ARID5A | AT-rich interaction domain 5A | 0.008237 | 1.253218 |
| 221810_at | RAB15 | RAB15, member RAS oncogene family | 0.008316 | 1.105073 |
| 211131_s_at | EDA | ectodysplasin A | 0.008316 | 1.062761 |
| 226187_at | CDS1 | CDP-diacylglycerol synthase 1 | 0.008393 | 1.03765 |
| 1552396_at | WFDC6 | WAP four-disulfide core domain 6 | 0.00849 | -1.21013 |
| 201442_s_at | ATP6AP2 | ATPase H+ transporting accessory protein 2 | 0.008634 | 1.151881 |
| 1554940_a_at | LOC388882 | uncharacterized LOC388882 | 0.008719 | 1.242733 |
| 216248_s_at | NR4A2 | nuclear receptor subfamily 4 group A member 2 | 0.008845 | 1.798601 |
| 218778_x_at | EPS8L1 | EPS8 like 1 | 0.008863 | -1.17528 |
| 219288_at | C3orf14 | chromosome 3 open reading frame 14 | 0.008866 | 1.251286 |
| 206568_at | TNP1 | transition protein 1 | 0.008882 | 1.007432 |
| 1552487_a_at | BNC1 | basonuclin 1 | 0.008905 | 1.168682 |
| 220843_s_at | DCAF13 | DDB1 and CUL4 associated factor 13 | 0.008983 | 1.419147 |
| 207548_at | LOC100996542///GRM7 | uncharacterized LOC100996542///glutamate metabotropic receptor 7 | 0.009033 | 1.233393 |
| 240117_at | FBN3 | fibrillin 3 | 0.009203 | 1.322611 |
| 232443_at | LOC441052 | uncharacterized LOC441052 | 0.009247 | 1.042686 |
| 219238_at | PIGV | phosphatidylinositol glycan anchor biosynthesis class V | 0.009284 | 1.034868 |
| 1561633_at | HMGA2 | high mobility group AT-hook 2 | 0.009309 | 1.196575 |
| 1566861_at | GATM | glycine amidinotransferase | 0.009353 | 1.084573 |
| 206422_at | GCG | glucagon | 0.009403 | 1.18305 |
| 1557865_at | LOC100505658 | uncharacterized LOC100505658 | 0.009469 | 1.084147 |
| 223197_s_at | SMARCAD1 | SWI/SNF-related, matrix-associated actin-dependent regulator of chromatin, subfamily a, containing DEAD/H box 1 | 0.00948 | -1.00036 |
| 243703_x_at | LIPE-AS1 | LIPE antisense RNA 1 | 0.009497 | 1.462427 |
| 207276_at | CDR1 | cerebellar degeneration related protein 1 | 0.009565 | 1.184162 |
| 206655_s_at | SEPT5-GP1BB///SEPT5///GP1BB | SEPT5-GP1BB readthrough///septin 5///glycoprotein Ib platelet beta subunit | 0.009755 | 1.148987 |
| 1554287_at | TRIM4 | tripartite motif containing 4 | 0.009929 | -1.23739 |
| 220800_s_at | TMOD3 | tropomodulin 3 | 0.010004 | 1.384142 |
| 205881_at | ZNF74 | zinc finger protein 74 | 0.010064 | 1.013786 |
| 220383_at | ABCG5 | ATP binding cassette subfamily G member 5 | 0.010074 | 1.353313 |
| 224434_s_at | WDR83 | WD repeat domain 83 | 0.010091 | 1.458658 |
| 1552619_a_at | ANLN | anillin actin binding protein | 0.010174 | 1.252162 |
| 1553079_at | TRIM40 | tripartite motif containing 40 | 0.01022 | -1.17751 |
| 1569701_at | PER3 | period circadian clock 3 | 0.01025 | -1.03952 |
| 240027_at | LIN7A | lin-7 homolog A, crumbs cell polarity complex component | 0.010441 | 1.344126 |
| 205209_at | ACVR1B | activin A receptor type 1B | 0.01047 | 1.218392 |
| 240265_at | TRAF3IP3 | TRAF3 interacting protein 3 | 0.010522 | 1.415527 |
| 204130_at | HSD11B2 | hydroxysteroid 11-beta dehydrogenase 2 | 0.010554 | 1.173586 |
| 237417_at | RCBTB1 | RCC1 and BTB domain containing protein 1 | 0.010577 | -1.19023 |
| 206249_at | MAP3K13 | mitogen-activated protein kinase kinase kinase 13 | 0.010589 | -1.05984 |
| 232082_x_at | SPRR3 | small proline rich protein 3 | 0.010632 | 1.029851 |
| 229065_at | SLC35F3 | solute carrier family 35 member F3 | 0.010704 | 1.030143 |
| 205081_at | CRIP1 | cysteine rich protein 1 | 0.010754 | 1.375641 |
| 234410_at | GABRR3 | gamma-aminobutyric acid type A receptor rho3 subunit (gene/pseudogene) | 0.010808 | 1.456038 |
| 210392_x_at | NR6A1 | nuclear receptor subfamily 6 group A member 1 | 0.010844 | 1.457174 |
| 1560934_at | LOC284669 | uncharacterized LOC284669 | 0.010857 | 1.02476 |
| 202284_s_at | CDKN1A | cyclin dependent kinase inhibitor 1A | 0.010885 | 1.175204 |
| 215276_at | WFDC8 | WAP four-disulfide core domain 8 | 0.010898 | 1.0286 |
| 1555745_a_at | LYZ | lysozyme | 0.010912 | 1.098649 |
| 232446_at | LOC90768 | uncharacterized LOC90768 | 0.010934 | 1.1757 |
| 239997_at | LOC780529 | uncharacterized LOC780529 | 0.010944 | 1.222477 |
| 204111_at | HNMT | histamine N-methyltransferase | 0.011029 | 1.058886 |
| 1569468_at | ZNF876P | zinc finger protein 876, pseudogene | 0.011123 | 1.643879 |
| 203713_s_at | LLGL2 | LLGL2, scribble cell polarity complex component | 0.011258 | 1.089842 |
| 243821_at | MRPS31 | mitochondrial ribosomal protein S31 | 0.011469 | 1.121837 |
| 244441_at | USP31 | ubiquitin specific peptidase 31 | 0.011543 | 1.293858 |
| 228672_at | ING5 | inhibitor of growth family member 5 | 0.011599 | 1.03773 |
| 220794_at | GREM2 | gremlin 2, DAN family BMP antagonist | 0.01162 | 1.370791 |
| 231158_x_at | PTBP1 | polypyrimidine tract binding protein 1 | 0.011711 | 1.047774 |
| 208579_x_at | H2BFS | H2B histone family member S | 0.011734 | 1.205629 |
| 205147_x_at | NCF4 | neutrophil cytosolic factor 4 | 0.011899 | 1.096452 |
| 243106_at | CLEC12A | C-type lectin domain family 12 member A | 0.011908 | 1.167707 |
| 220430_at | FAM110D | family with sequence similarity 110 member D | 0.011965 | 1.046574 |
| 206603_at | SLC2A4 | solute carrier family 2 member 4 | 0.012049 | -1.06624 |
| 206785_s_at | KLRC2///KLRC1 | killer cell lectin like receptor C2///killer cell lectin like receptor C1 | 0.012082 | 1.398068 |
| 222727_s_at | SLC8B1 | solute carrier family 8 member B1 | 0.012142 | 1.355818 |
| 210546_x_at | CTAG1A///CTAG1B | cancer/testis antigen 1A///cancer/testis antigen 1B | 0.012146 | 1.14027 |
| 203927_at | NFKBIE | NFKB inhibitor epsilon | 0.012166 | 1.020462 |
| 223774_at | SNORA16A///SNORA61///SNORA44///SNHG12 | small nucleolar RNA, H/ACA box 16A///small nucleolar RNA, H/ACA box 61///small nucleolar RNA, H/ACA box 44///small nucleolar RNA host gene 12 | 0.012179 | 1.474203 |
| 216238_s_at | FGB | fibrinogen beta chain | 0.01218 | 1.146344 |
| 231798_at | NOG | noggin | 0.012276 | -1.13935 |
| 223767_at | GPR84 | G protein-coupled receptor 84 | 0.012342 | 1.370495 |
| 227644_at | RIMS4 | regulating synaptic membrane exocytosis 4 | 0.012387 | -1.10601 |
| 219488_at | A4GALT | alpha 1,4-galactosyltransferase | 0.012401 | 1.164715 |
| 213419_at | APBB2 | amyloid beta precursor protein binding family B member 2 | 0.012554 | -1.10114 |
| 243335_at | P4HA1 | prolyl 4-hydroxylase subunit alpha 1 | 0.012622 | 1.364637 |
| 1554697_at | ADAMTS9 | ADAM metallopeptidase with thrombospondin type 1 motif 9 | 0.012644 | 1.132112 |
| 243600_at | TPGS1 | tubulin polyglutamylase complex subunit 1 | 0.01266 | -1.1116 |
| 1563221_at | LOC414300 | uncharacterized LOC414300 | 0.01272 | 1.295585 |
| 243679_at | JPH3 | junctophilin 3 | 0.012784 | 1.017453 |
| 216365_x_at | IGLJ3///CKAP2 | immunoglobulin lambda joining 3///cytoskeleton associated protein 2 | 0.012829 | 1.087776 |
| 225807_at | AJUBA | ajuba LIM protein | 0.012854 | 1.212044 |
| 201789_at | PCNX4 | pecanex homolog 4 (Drosophila) | 0.012897 | -1.41367 |
| 224179_s_at | MIOX | myo-inositol oxygenase | 0.012913 | 1.203045 |
| 1554728_at | SLC9A1 | solute carrier family 9 member A1 | 0.01293 | 1.195791 |
| 201187_s_at | ITPR3 | inositol 1,4,5-trisphosphate receptor type 3 | 0.012937 | -1.04887 |
| 244499_at | THAP2 | THAP domain containing 2 | 0.012946 | 1.142472 |
| 236009_at | PERP | PERP, TP53 apoptosis effector | 0.012994 | -1.04591 |
| 1552458_at | MBD3L1 | methyl-CpG binding domain protein 3 like 1 | 0.013059 | 1.29841 |
| 202431_s_at | MYC | v-myc avian myelocytomatosis viral oncogene homolog | 0.013156 | 1.281933 |
| 233852_at | POLH | DNA polymerase eta | 0.013166 | -1.16493 |
| 243680_at | LOC100506476 | uncharacterized LOC100506476 | 0.013197 | -1.45198 |
| 220359_s_at | ARPP21 | cAMP regulated phosphoprotein 21 | 0.01321 | 1.152573 |
| 224053_s_at | SLC4A9 | solute carrier family 4 member 9 | 0.013235 | -1.00157 |
| 208329_at | PBOV1 | prostate and breast cancer overexpressed 1 | 0.013236 | 1.13891 |
| 230051_at | PROSER2 | proline and serine rich 2 | 0.013243 | 1.18834 |
| 225451_at | GRIPAP1 | GRIP1 associated protein 1 | 0.013248 | 1.118986 |
| 221340_at | CDX4 | caudal type homeobox 4 | 0.013259 | 1.108033 |
| 207457_s_at | LY6G6F///LY6G6D | lymphocyte antigen 6 complex, locus G6F///lymphocyte antigen 6 complex, locus G6D | 0.013296 | 1.168538 |
| 1560527_at | NFE4 | nuclear factor, erythroid 4 | 0.01333 | 1.14647 |
| 237139_at | PDE9A | phosphodiesterase 9A | 0.013653 | 1.041237 |
| 215732_s_at | LOC102725292///DTX2 | probable E3 ubiquitin-protein ligase DTX2-like///deltex E3 ubiquitin ligase 2 | 0.013848 | 1.102084 |
| 1566251_at | SH3GL1P1 | SH3 domain containing GRB2 like 1, endophilin A2 pseudogene 1 | 0.013976 | 1.37508 |
| 232303_at | ZNF608 | zinc finger protein 608 | 0.014089 | 1.028045 |
| 240366_at | LHFPL3-AS1 | LHFPL3 antisense RNA 1 | 0.014095 | 1.190411 |
| 1553515_at | COPS9 | COP9 signalosome subunit 9 | 0.014099 | 1.366013 |
| 1557215_at | LINC00648 | long intergenic non-protein coding RNA 648 | 0.014188 | 1.315878 |
| 1565910_at | FSTL4 | follistatin like 4 | 0.014289 | 1.448062 |
| 207977_s_at | DPT | dermatopontin | 0.014355 | 1.232976 |
| 244170_at | RAB3C | RAB3C, member RAS oncogene family | 0.014369 | 1.638666 |
| 1553675_at | KLC4 | kinesin light chain 4 | 0.014405 | 1.244582 |
| 223912_s_at | CLN8 | ceroid-lipofuscinosis, neuronal 8 | 0.014447 | -1.03203 |
| 227297_at | ITGA9 | integrin subunit alpha 9 | 0.01455 | 1.12267 |
| 214031_s_at | KRT7 | keratin 7 | 0.014561 | 1.469948 |
| 243978_at | FAM65C | family with sequence similarity 65 member C | 0.014686 | 1.244748 |
| 203649_s_at | PLA2G2A | phospholipase A2 group IIA | 0.014745 | 1.042993 |
| 210059_s_at | MAPK13 | mitogen-activated protein kinase 13 | 0.014749 | 1.248243 |
| 203867_s_at | NLE1 | notchless homolog 1 | 0.014797 | 1.115277 |
| 203828_s_at | IL32 | interleukin 32 | 0.014864 | 1.625044 |
| 1555102_at | FGF7 | fibroblast growth factor 7 | 0.014887 | 1.826526 |
| 1564485_at | LINC00887 | long intergenic non-protein coding RNA 887 | 0.014976 | 1.471509 |
| 219408_at | PRMT7 | protein arginine methyltransferase 7 | 0.015017 | 1.290974 |
| 211861_x_at | CD28 | CD28 molecule | 0.015054 | 1.327008 |
| 1562440_at | MAP3K13 | mitogen-activated protein kinase kinase kinase 13 | 0.015149 | -1.3403 |
| 221236_s_at | STMN4 | stathmin 4 | 0.015228 | 1.184665 |
| 220456_at | SPTLC3 | serine palmitoyltransferase long chain base subunit 3 | 0.015254 | 1.250134 |
| 1405_i_at | CCL5 | C-C motif chemokine ligand 5 | 0.015301 | 1.313845 |
| 205951_at | MYH1 | myosin heavy chain 1 | 0.015355 | 1.058934 |
| 236640_at | LOC100507165 | uncharacterized LOC100507165 | 0.015366 | -1.12954 |
| 1561403_at | SOHLH1 | spermatogenesis and oogenesis specific basic helix-loop-helix 1 | 0.015387 | -1.32519 |
| 1570080_at | HIPK1-AS1 | HIPK1 antisense RNA 1 | 0.015441 | 1.111777 |
| 1562368_at | CARD11 | caspase recruitment domain family member 11 | 0.015453 | 1.097578 |
| 211728_s_at | HYAL3 | hyaluronoglucosaminidase 3 | 0.015537 | 1.281536 |
| 208129_x_at | LOC101928269///LOC100506403///RUNX1 | uncharacterized LOC101928269///uncharacterized LOC100506403///runt related transcription factor 1 | 0.015561 | 1.072651 |
| 222362_at | AGFG2 | ArfGAP with FG repeats 2 | 0.01559 | 1.351056 |
| 201374_x_at | PPP2CB | protein phosphatase 2 catalytic subunit beta | 0.015614 | 1.058686 |
| 215443_at | TSHR | thyroid stimulating hormone receptor | 0.015663 | 1.255847 |
| 237577_at | PCNP | PEST proteolytic signal containing nuclear protein | 0.015765 | -1.1734 |
| 1553409_at | ADAMTS20 | ADAM metallopeptidase with thrombospondin type 1 motif 20 | 0.015796 | 1.481561 |
| 211820_x_at | GYPA | glycophorin A (MNS blood group) | 0.015876 | -1.10426 |
| 213745_at | ATRNL1 | attractin like 1 | 0.015986 | 1.644892 |
| 206464_at | BMX | BMX non-receptor tyrosine kinase | 0.016061 | 1.045767 |
| 232170_at | S100A7A | S100 calcium binding protein A7A | 0.016061 | 1.16634 |
| 234976_x_at | MTHFD2 | methylenetetrahydrofolate dehydrogenase (NADP+ dependent) 2, methenyltetrahydrofolate cyclohydrolase | 0.016129 | 1.178876 |
| 1558887_at | ZNF816-ZNF321P///ZNF321P | ZNF816-ZNF321P readthrough///zinc finger protein 321, pseudogene | 0.016135 | 1.256786 |
| 220116_at | KCNN2 | potassium calcium-activated channel subfamily N member 2 | 0.016168 | -1.24756 |
| 232173_at | CLEC2L | C-type lectin domain family 2 member L | 0.016258 | 1.095629 |
| 219562_at | RAB26 | RAB26, member RAS oncogene family | 0.016263 | 1.015859 |
| 220219_s_at | LRRC37A2///LRRC37A3///LRRC37A4P///LRRC37A | leucine rich repeat containing 37 member A2///leucine rich repeat containing 37 member A3///leucine rich repeat containing 37 member A4, pseudogene///leucine rich repeat containing 37A | 0.016408 | 1.043468 |
| 1555485_s_at | LOC100507387///FAM153A///FAM153B | uncharacterized LOC100507387///family with sequence similarity 153 member A///family with sequence similarity 153 member B | 0.016411 | 1.143489 |
| 232426_at | SV2B | synaptic vesicle glycoprotein 2B | 0.016422 | 1.199083 |
| 208133_at | RFC1 | replication factor C subunit 1 | 0.016425 | 1.256699 |
| 1559655_at | MAMDC2-AS1 | MAMDC2 antisense RNA 1 | 0.016436 | 1.454615 |
| 237137_at | SCARNA2 | small Cajal body-specific RNA 2 | 0.016467 | 1.171229 |
| 233934_at | MIR490///LOC349160 | microRNA 490///uncharacterized LOC349160 | 0.016482 | -1.00843 |
| 213433_at | ARL3 | ADP ribosylation factor like GTPase 3 | 0.016567 | 1.117768 |
| 209924_at | CCL18 | C-C motif chemokine ligand 18 | 0.016844 | 1.091844 |
| 208555_x_at | CST2 | cystatin SA | 0.016881 | -1.02397 |
| 1556309_s_at | FAAP20 | Fanconi anemia core complex associated protein 20 | 0.016946 | 1.139849 |
| 202533_s_at | DHFR | dihydrofolate reductase | 0.017012 | 1.417057 |
| 210224_at | MR1 | major histocompatibility complex, class I-related | 0.017031 | 1.126577 |
| 244493_at | GPR22 | G protein-coupled receptor 22 | 0.017091 | 1.5885 |
| 206110_at | HIST1H3F///HIST1H3B///HIST1H3H///HIST1H3J///HIST1H3G///HIST1H3I///HIST1H3E///HIST1H3C///HIST1H3D///HIST1H3A | histone cluster 1, H3f///histone cluster 1, H3b///histone cluster 1, H3h///histone cluster 1, H3j///histone cluster 1, H3g///histone cluster 1, H3i///histone cluster 1, H3e///histone cluster 1, H3c///histone cluster 1, H3d///histone cluster 1, H3a | 0.017119 | 1.433705 |
| 241034_at | GLS | glutaminase | 0.017133 | -1.17639 |
| 202463_s_at | MBD3 | methyl-CpG binding domain protein 3 | 0.017253 | 1.191735 |
| 232801_at | ERCC6L2 | ERCC excision repair 6 like 2 | 0.017261 | 1.348428 |
| 34210_at | CD52 | CD52 molecule | 0.017284 | 1.112976 |
| 232672_x_at | CTXN2///SLC24A5 | cortexin 2///solute carrier family 24 member 5 | 0.0173 | 1.115681 |
| 1569599_at | SAMSN1 | SAM domain, SH3 domain and nuclear localization signals 1 | 0.017346 | 1.436115 |
| 242087_x_at | MFI2-AS1 | MFI2 antisense RNA 1 | 0.01737 | -1.09044 |
| 208343_s_at | NR5A2 | nuclear receptor subfamily 5 group A member 2 | 0.017453 | -1.29839 |
| 243282_at | CCDC93 | coiled-coil domain containing 93 | 0.017595 | -1.05296 |
| 209963_s_at | EPOR | erythropoietin receptor | 0.017644 | 1.067692 |
| 1552570_at | TSPEAR | thrombospondin type laminin G domain and EAR repeats | 0.01765 | 1.30614 |
| 233416_at | GNG8 | G protein subunit gamma 8 | 0.017671 | 1.316563 |
| 205612_at | MMRN1 | multimerin 1 | 0.01774 | 1.058307 |
| 220054_at | IL23A | interleukin 23 subunit alpha | 0.017825 | 1.186275 |
| 210562_at | GREB1 | growth regulation by estrogen in breast cancer 1 | 0.017837 | 1.154486 |
| 217034_at | LOC101928457 | uncharacterized LOC101928457 | 0.017998 | 1.014177 |
| 235395_at | SEC63 | SEC63 homolog, protein translocation regulator | 0.018004 | 1.168781 |
| 1555324_at | PTK7 | protein tyrosine kinase 7 (inactive) | 0.018025 | 1.141539 |
| 215437_x_at | BAZ2A | bromodomain adjacent to zinc finger domain 2A | 0.018059 | 1.373648 |
| 205948_at | PTPRT | protein tyrosine phosphatase, receptor type T | 0.018098 | 1.034712 |
| 217655_at | LOC100127972 | uncharacterized LOC100127972 | 0.018197 | -1.07385 |
| 237730_at | TP53TG3HP | TP53 target 3 family member H, pseudogene | 0.018205 | -1.09581 |
| 237685_at | LOC101929926 | uncharacterized LOC101929926 | 0.018213 | 1.384058 |
| 206894_at | APOA4 | apolipoprotein A4 | 0.018214 | 1.047131 |
| 1569385_s_at | TET2 | tet methylcytosine dioxygenase 2 | 0.018251 | 1.227975 |
| 211478_s_at | DPP4 | dipeptidyl peptidase 4 | 0.018319 | 1.285701 |
| 207080_s_at | PYY | peptide YY | 0.018343 | 1.286882 |
| 238008_at | PRR18 | proline rich 18 | 0.018366 | 1.221548 |
| 232015_at | GAREM2 | GRB2 associated regulator of MAPK1 subtype 2 | 0.018368 | 1.316392 |
| 234167_at | NIFK | nucleolar protein interacting with the FHA domain of MKI67 | 0.018392 | 1.200221 |
| 1553474_at | LOC100288966 | POTE ankyrin domain family member D-like | 0.018491 | 1.468801 |
| 233298_at | CCDC169 | coiled-coil domain containing 169 | 0.01852 | -1.14597 |
| 1558163_at | PEX13 | peroxisomal biogenesis factor 13 | 0.018533 | -1.1067 |
| 231786_at | HOXA13 | homeobox A13 | 0.018552 | -1.25249 |
| 207399_at | BFSP2 | beaded filament structural protein 2 | 0.018618 | 1.247848 |
| 1562414_at | LOC100509814 | uncharacterized LOC100509814 | 0.01871 | 1.107385 |
| 1566888_at | THADA | THADA, armadillo repeat containing | 0.018723 | 1.259891 |
| 1561450_at | LOC101928618 | uncharacterized LOC101928618 | 0.01884 | -1.4932 |
| 1569086_at | EML2-AS1 | EML2 antisense RNA 1 | 0.018873 | -1.04776 |
| 229706_at | TCERG1 | transcription elongation regulator 1 | 0.018884 | -1.01993 |
| 207640_x_at | NTN3 | netrin 3 | 0.019094 | 1.151524 |
| 240444_x_at | CLIP1 | CAP-Gly domain containing linker protein 1 | 0.019131 | 1.483423 |
| 1569054_at | SLC1A3 | solute carrier family 1 member 3 | 0.01922 | -1.04286 |
| 1554967_at | DIP2A | disco interacting protein 2 homolog A | 0.019297 | 1.124343 |
| 1552722_at | ARPP21 | cAMP regulated phosphoprotein 21 | 0.019307 | 1.258832 |
| 1561237_at | LOC101929657 | uncharacterized LOC101929657 | 0.019363 | -1.11814 |
| 1552365_at | SCIN | scinderin | 0.019444 | 1.535154 |
| 220508_at | CCT8L2 | chaperonin containing TCP1 subunit 8 like 2 | 0.019451 | 1.149235 |
| 1559548_at | ACVR2B | activin A receptor type 2B | 0.019626 | -1.06505 |
| 1554784_at | CNTN1 | contactin 1 | 0.019718 | 1.147075 |
| 231470_at | LINC00664 | long intergenic non-protein coding RNA 664 | 0.019726 | 1.34401 |
| 224559_at | MALAT1 | metastasis associated lung adenocarcinoma transcript 1 (non-protein coding) | 0.019798 | -1.13728 |
| 1552897_a_at | KCNG3 | potassium voltage-gated channel modifier subfamily G member 3 | 0.02007 | 1.308218 |
| 217605_at | USP27X | ubiquitin specific peptidase 27, X-linked | 0.020071 | 1.162176 |
| 241716_at | HSPD1 | heat shock protein family D (Hsp60) member 1 | 0.020078 | -1.37806 |
| 1567179_at | LOC105376944 | uncharacterized LOC105376944 | 0.020099 | 1.369527 |
| 220863_at | MIP | major intrinsic protein of lens fiber | 0.02011 | 1.14098 |
| 1558420_at | C14orf180 | chromosome 14 open reading frame 180 | 0.020199 | 1.085598 |
| 208606_s_at | WNT4 | Wnt family member 4 | 0.02028 | 1.093747 |
| 217439_at | LOC101928278 | uncharacterized LOC101928278 | 0.020297 | 1.244399 |
| 203418_at | CCNA2 | cyclin A2 | 0.020395 | 1.414643 |
| 1566860_at | GATM | glycine amidinotransferase | 0.020513 | 1.109719 |
| 244190_at | THAP5 | THAP domain containing 5 | 0.020527 | -1.42961 |
| 221243_s_at | PRO1596 | uncharacterized LOC29013 | 0.020543 | -1.17013 |
| 236666_s_at | LRRC10B | leucine rich repeat containing 10B | 0.020564 | -1.1052 |
| 1552671_a_at | SLC9A7 | solute carrier family 9 member A7 | 0.020653 | 1.052911 |
| 219101_x_at | ABHD8 | abhydrolase domain containing 8 | 0.020828 | -1.14957 |
| 239040_at | HNRNPD | heterogeneous nuclear ribonucleoprotein D | 0.020888 | -1.05741 |
| 238296_at | GLIPR1L1 | GLI pathogenesis related 1 like 1 | 0.02089 | 1.092838 |
| 217171_at | SMPD1 | sphingomyelin phosphodiesterase 1 | 0.020893 | 1.076773 |
| 211855_s_at | SLC25A14 | solute carrier family 25 member 14 | 0.020914 | 1.193855 |
| 232739_at | SPIB | Spi-B transcription factor | 0.021012 | 1.059291 |
| 223567_at | SEMA6B | semaphorin 6B | 0.021034 | 1.396403 |
| 206777_s_at | CRYBB2P1///CRYBB2 | crystallin beta B2 pseudogene 1///crystallin beta B2 | 0.021104 | 1.304785 |
| 233295_at | VN1R108P | vomeronasal 1 receptor 108 pseudogene | 0.021133 | 1.286429 |
| 1554156_a_at | WFDC8 | WAP four-disulfide core domain 8 | 0.021178 | 1.551388 |
| 219745_at | MFSD13A | major facilitator superfamily domain containing 13A | 0.02126 | 1.015377 |
| 224401_s_at | FCRL4 | Fc receptor like 4 | 0.02132 | 1.272397 |
| 1552373_s_at | C4orf33 | chromosome 4 open reading frame 33 | 0.021505 | 1.272502 |
| 205509_at | CPB1 | carboxypeptidase B1 | 0.021538 | -1.2076 |
| 214836_x_at | IGK///IGKC | immunoglobulin kappa locus///immunoglobulin kappa constant | 0.021552 | 1.064892 |
| 234458_at | ANPEP | alanyl aminopeptidase, membrane | 0.021554 | 1.129411 |
| 228181_at | SLC30A1 | solute carrier family 30 member 1 | 0.021612 | -1.17825 |
| 1556809_a_at | RAP2A | RAP2A, member of RAS oncogene family | 0.021622 | 1.292441 |
| 234709_at | CAPN13 | calpain 13 | 0.021684 | -1.30813 |
| 215937_at | PTGDR | prostaglandin D2 receptor | 0.021907 | 1.29156 |
| 239321_at | LOC441454 | prothymosin, alpha pseudogene | 0.022133 | -1.16675 |
| 211092_s_at | NF2 | neurofibromin 2 | 0.022137 | 1.333209 |
| 242137_at | RBMS3 | RNA binding motif single stranded interacting protein 3 | 0.022142 | -1.21431 |
| 1566967_at | SPRY4-IT1 | SPRY4 intronic transcript 1 | 0.022172 | 1.00099 |
| 230861_at | DKFZP434L187 | uncharacterized LOC26082 | 0.022223 | -1.25472 |
| 210764_s_at | CYR61 | cysteine rich angiogenic inducer 61 | 0.022395 | 1.037914 |
| 1568974_at | NHEG1 | neuroblastoma highly expressed 1 | 0.0224 | -1.00955 |
| 231900_at | ZDHHC18 | zinc finger DHHC-type containing 18 | 0.022436 | 1.06112 |
| 221360_s_at | GHSR | growth hormone secretagogue receptor | 0.022542 | 1.096801 |
| 218848_at | THOC6 | THO complex 6 | 0.02257 | 1.080579 |
| 242927_at | DCLRE1C | DNA cross-link repair 1C | 0.022593 | 1.293321 |
| 238214_at | LRRC69 | leucine rich repeat containing 69 | 0.022659 | -1.01093 |
| 237411_at | ADAMTS6 | ADAM metallopeptidase with thrombospondin type 1 motif 6 | 0.022788 | -1.05741 |
| 229140_at | ZNF579 | zinc finger protein 579 | 0.022804 | 1.213025 |
| 1561708_at | MB21D1 | Mab-21 domain containing 1 | 0.022842 | -1.05206 |
| 233645_s_at | C1RL | complement C1r subcomponent like | 0.023185 | 1.124843 |
| 227673_at | ZNRD1 | zinc ribbon domain containing 1 | 0.023206 | -1.092 |
| 211631_x_at | B4GALT1 | beta-1,4-galactosyltransferase 1 | 0.023207 | 1.070652 |
| 231941_s_at | MUC20 | mucin 20, cell surface associated | 0.023305 | 1.442089 |
| 243984_at | SCRG1 | stimulator of chondrogenesis 1 | 0.023385 | 1.171678 |
| 242560_at | FANCD2 | Fanconi anemia complementation group D2 | 0.023424 | 1.138318 |
| 217378_x_at | IGKV1OR2-108 | immunoglobulin kappa variable 1/OR2-108 (non-functional) | 0.023428 | 1.168923 |
| 205591_at | OLFM1 | olfactomedin 1 | 0.023437 | 1.414343 |
| 206693_at | IL7 | interleukin 7 | 0.023512 | -1.12306 |
| 230863_at | LRP2 | LDL receptor related protein 2 | 0.02362 | 1.273146 |
| 224403_at | FCRL4 | Fc receptor like 4 | 0.023671 | -1.1743 |
| 1553077_at | SDR9C7 | short chain dehydrogenase/reductase family 9C, member 7 | 0.023745 | 1.106499 |
| 1561521_at | S100B | S100 calcium binding protein B | 0.023808 | 1.229939 |
| 216542_x_at | IGHM///IGHG1///IGHA1 | immunoglobulin heavy constant mu///immunoglobulin heavy constant gamma 1 (G1m marker)///immunoglobulin heavy constant alpha 1 | 0.023853 | -1.04001 |
| 1569156_at | LOC100505555///ZNF846 | uncharacterized LOC100505555///zinc finger protein 846 | 0.023871 | -1.17263 |
| 1553420_at | SATB2-AS1 | SATB2 antisense RNA 1 | 0.024389 | 1.273729 |
| 1564383_s_at | FLJ35934 | FLJ35934 | 0.024467 | -1.33233 |
| 211634_x_at | IGHM | immunoglobulin heavy constant mu | 0.024477 | 1.041492 |
| 219712_s_at | SHPK///TRPV1 | sedoheptulokinase///transient receptor potential cation channel subfamily V member 1 | 0.02459 | 1.051116 |
| 231339_at | TSPYL6 | TSPY like 6 | 0.024691 | 1.456567 |
| 1554308_s_at | GABRA2 | gamma-aminobutyric acid type A receptor alpha2 subunit | 0.024854 | -1.42951 |
| 1563834_a_at | AKNAD1 | AKNA domain containing 1 | 0.024897 | 1.032817 |
| 230153_at | NEK9 | NIMA related kinase 9 | 0.024926 | 1.069482 |
| 224583_at | COTL1 | coactosin like F-actin binding protein 1 | 0.02498 | 1.350673 |
| 1570307_s_at | ST18 | ST18, C2H2C-type zinc finger | 0.025188 | 1.041462 |
| 1557014_a_at | FAM201A | family with sequence similarity 201 member A | 0.025198 | 1.412841 |
| 206521_s_at | GTF2A1 | general transcription factor IIA subunit 1 | 0.025305 | 1.179323 |
| 209810_at | SFTPB | surfactant protein B | 0.025379 | 1.066917 |
| 1557498_a_at | LINC01487 | long intergenic non-protein coding RNA 1487 | 0.025415 | 1.265608 |
| 229430_at | C8orf46 | chromosome 8 open reading frame 46 | 0.02543 | -1.00244 |
| 220354_at | MCF2L-AS1 | MCF2L antisense RNA 1 | 0.025436 | 1.111914 |
| 203820_s_at | IGF2BP3 | insulin like growth factor 2 mRNA binding protein 3 | 0.02548 | 1.253245 |
| 223456_s_at | TCHP | trichoplein keratin filament binding | 0.025615 | 1.134173 |
| 208571_at | ANP32D | acidic nuclear phosphoprotein 32 family member D | 0.025637 | 1.228259 |
| 1562234_a_at | NAV3 | neuron navigator 3 | 0.025811 | 1.243725 |
| 206341_at | IL2RA | interleukin 2 receptor subunit alpha | 0.025831 | -1.04562 |
| 1553183_at | UMODL1 | uromodulin like 1 | 0.025951 | 1.511144 |
| 221933_at | NLGN4X | neuroligin 4, X-linked | 0.025959 | -1.50082 |
| 235522_at | CLEC2D | C-type lectin domain family 2 member D | 0.025977 | -1.14339 |
| 210994_x_at | TRIM23 | tripartite motif containing 23 | 0.026135 | -1.13991 |
| 210019_at | CALML3 | calmodulin like 3 | 0.026169 | 1.028637 |
| 232415_at | PCDHB13 | protocadherin beta 13 | 0.026173 | -1.42269 |
| 218725_at | SLC25A22 | solute carrier family 25 member 22 | 0.026207 | 1.210361 |
| 240420_at | AADACL2 | arylacetamide deacetylase like 2 | 0.026208 | -1.00776 |
| 1559206_at | PSEN1 | presenilin 1 | 0.026231 | 1.256045 |
| 1560595_at | LOC100128993 | uncharacterized LOC100128993 | 0.026272 | 1.293355 |
| 214883_at | THRA | thyroid hormone receptor, alpha | 0.026293 | 1.082358 |
| 1557692_a_at | GATM | glycine amidinotransferase | 0.026312 | -1.30529 |
| 202196_s_at | DKK3 | dickkopf WNT signaling pathway inhibitor 3 | 0.026324 | -1.12933 |
| 220872_at | PRO2964 | uncharacterized protein PRO2964 | 0.026393 | 1.081994 |
| 238049_at | GRAMD3 | GRAM domain containing 3 | 0.026432 | 1.027802 |
| 1569431_at | PAFAH1B2 | platelet activating factor acetylhydrolase 1b catalytic subunit 2 | 0.026911 | 1.122213 |
| 206382_s_at | BDNF | brain derived neurotrophic factor | 0.027065 | 1.069471 |
| 1564072_at | MYH16 | myosin heavy chain 16 pseudogene | 0.027259 | 1.13261 |
| 1568924_a_at | IQUB | IQ motif and ubiquitin domain containing | 0.027283 | 1.118537 |
| 243036_at | CCDC30 | coiled-coil domain containing 30 | 0.027408 | 1.031573 |
| 219589_s_at | TMEM143 | transmembrane protein 143 | 0.027483 | 1.051579 |
| 203559_s_at | AOC1 | amine oxidase, copper containing 1 | 0.027667 | 1.163625 |
| 219478_at | WFDC1 | WAP four-disulfide core domain 1 | 0.027959 | 1.160216 |
| 1553852_at | VPS13B | vacuolar protein sorting 13 homolog B | 0.028184 | 1.030615 |
| 211766_s_at | PNLIPRP2 | pancreatic lipase related protein 2 (gene/pseudogene) | 0.028478 | 1.010142 |
| 1555301_a_at | DIP2A | disco interacting protein 2 homolog A | 0.028541 | -1.03338 |
| 235455_at | FAM131C | family with sequence similarity 131 member C | 0.028545 | 1.050718 |
| 230747_s_at | TTC39C | tetratricopeptide repeat domain 39C | 0.028619 | 1.223383 |
| 1569191_at | ZNF826P | zinc finger protein 826, pseudogene | 0.028636 | -1.37219 |
| 234847_at | LINC00309 | long intergenic non-protein coding RNA 309 | 0.028642 | 1.09994 |
| 1554920_at | SCEL | sciellin | 0.028725 | 1.271768 |
| 203670_at | ARPC4-TTLL3///TTLL3///ARPC4 | ARPC4-TTLL3 readthrough///tubulin tyrosine ligase like 3///actin related protein 2/3 complex subunit 4 | 0.028744 | 1.038476 |
| 1556876_s_at | LOC101930578///TPTE2P2 | uncharacterized LOC101930578///transmembrane phosphoinositide 3-phosphatase and tensin homolog 2 pseudogene 2 | 0.028751 | 1.031291 |
| 237024_at | LSMEM2 | leucine rich single-pass membrane protein 2 | 0.028924 | 1.027549 |
| 231980_at | DOK6 | docking protein 6 | 0.029159 | 1.1886 |
| 1552661_at | PCDHGB7 | protocadherin gamma subfamily B, 7 | 0.02916 | 1.879776 |
| 240428_at | LOC285000 | uncharacterized LOC285000 | 0.029662 | -1.05766 |
| 206730_at | GRIA3 | glutamate ionotropic receptor AMPA type subunit 3 | 0.029713 | 1.033902 |
| 208233_at | PDPN | podoplanin | 0.029726 | 1.170907 |
| 220146_at | TLR7 | toll like receptor 7 | 0.029814 | 1.146587 |
| 209908_s_at | TGFB2 | transforming growth factor beta 2 | 0.029873 | 1.119163 |
| 205984_at | CRHBP | corticotropin releasing hormone binding protein | 0.029881 | -1.02048 |
| 207661_s_at | SH3PXD2A | SH3 and PX domains 2A | 0.030006 | -1.09427 |
| 1555071_at | TLL1 | tolloid like 1 | 0.030213 | -1.10988 |
| 1562107_at | LOC101928833 | uncharacterized LOC101928833 | 0.030228 | 1.199226 |
| 216876_s_at | IL17A | interleukin 17A | 0.030301 | 1.067261 |
| 213490_s_at | MAP2K2 | mitogen-activated protein kinase kinase 2 | 0.030356 | 1.088403 |
| 232843_s_at | DOCK8 | dedicator of cytokinesis 8 | 0.030415 | -1.24956 |
| 223925_s_at | MTPN | myotrophin | 0.030479 | 1.048109 |
| 1559975_at | BTG1 | BTG anti-proliferation factor 1 | 0.030602 | 1.296321 |
| 221182_at | MROH9 | maestro heat like repeat family member 9 | 0.030992 | 1.34222 |
| 209730_at | SEMA3F | semaphorin 3F | 0.030994 | 1.006287 |
| 229159_at | THSD7A | thrombospondin type 1 domain containing 7A | 0.031101 | -1.33591 |
| 224230_at | IL36B | interleukin 36, beta | 0.03132 | 1.039423 |
| 1558941_at | ZNF704 | zinc finger protein 704 | 0.031339 | 1.184503 |
| 237697_at | LOC101928269///LOC100506403///RUNX1 | uncharacterized LOC101928269///uncharacterized LOC100506403///runt related transcription factor 1 | 0.031483 | -1.00563 |
| 210127_at | RAB6B | RAB6B, member RAS oncogene family | 0.031891 | 1.052662 |
| 236693_at | MIR124-2HG | MIR124-2 host gene | 0.032078 | 1.076947 |
| 216944_s_at | ITPR1 | inositol 1,4,5-trisphosphate receptor type 1 | 0.032227 | 1.09283 |
| 1553467_at | FLJ32742///DCAF8L2 | uncharacterized locus FLJ32742///DDB1 and CUL4 associated factor 8 like 2 | 0.032268 | -1.1472 |
| 1554841_at | MTHFD2L | methylenetetrahydrofolate dehydrogenase (NADP+ dependent) 2-like | 0.032448 | 1.026101 |
| 233069_at | PPP4R1L | protein phosphatase 4 regulatory subunit 1 like (pseudogene) | 0.032467 | 1.036963 |
| 238290_at | LOC101929464 | uncharacterized LOC101929464 | 0.032548 | -1.02011 |
| 220249_at | HYAL4 | hyaluronoglucosaminidase 4 | 0.032598 | -1.25919 |
| 203148_s_at | TRIM14 | tripartite motif containing 14 | 0.032647 | 1.026851 |
| 1556793_a_at | FAM83C | family with sequence similarity 83 member C | 0.032673 | 1.036016 |
| 1565673_at | FCGR2A | Fc fragment of IgG receptor IIa | 0.032683 | 1.076026 |
| 240713_s_at | LINC00403 | long intergenic non-protein coding RNA 403 | 0.032843 | 1.216544 |
| 230633_at | TMEM102 | transmembrane protein 102 | 0.032966 | 1.036619 |
| 214032_at | ZAP70 | zeta chain of T cell receptor associated protein kinase 70 | 0.032984 | -1.0683 |
| 216839_at | LAMA2 | laminin subunit alpha 2 | 0.03308 | -1.32146 |
| 213965_s_at | CHD5 | chromodomain helicase DNA binding protein 5 | 0.03319 | 1.007263 |
| 1555666_at | PTPRS | protein tyrosine phosphatase, receptor type S | 0.033349 | 1.014151 |
| 207029_at | KITLG | KIT ligand | 0.033355 | 1.337944 |
| 205668_at | LY75 | lymphocyte antigen 75 | 0.033372 | 1.173454 |
| 1554261_at | KLHL29 | kelch like family member 29 | 0.03353 | 1.128049 |
| 1558588_at | LOC100996741///LINC00869 | uncharacterized LOC100996741///long intergenic non-protein coding RNA 869 | 0.033726 | 1.11032 |
| 216992_s_at | GRM8 | glutamate metabotropic receptor 8 | 0.033778 | 1.141947 |
| 203785_s_at | DDX28 | DEAD-box helicase 28 | 0.033804 | 1.145233 |
| 1556131_s_at | FBF1 | Fas binding factor 1 | 0.033807 | 1.145385 |
| 1562365_at | LOC286177 | uncharacterized LOC286177 | 0.034113 | -1.19005 |
| 213172_at | TTC9 | tetratricopeptide repeat domain 9 | 0.034205 | -1.4615 |
| 220410_s_at | CAMSAP1 | calmodulin regulated spectrin associated protein 1 | 0.034229 | 1.095322 |
| 233098_s_at | EPB41L4B | erythrocyte membrane protein band 4.1 like 4B | 0.034279 | 1.234791 |
| 235182_at | ISM1 | isthmin 1, angiogenesis inhibitor | 0.034356 | -1.03859 |
| 215733_x_at | CTAG2 | cancer/testis antigen 2 | 0.034357 | 1.012665 |
| 222546_s_at | EPS8L2 | EPS8 like 2 | 0.034434 | -1.03979 |
| 1556200_a_at | C10orf90 | chromosome 10 open reading frame 90 | 0.034439 | 1.195877 |
| 230698_at | CALN1 | calneuron 1 | 0.034456 | 1.21144 |
| 235873_at | LOC100506235 | uncharacterized LOC100506235 | 0.034611 | -1.01061 |
| 1553706_at | HTRA4 | HtrA serine peptidase 4 | 0.034632 | 1.473373 |
| 239447_at | TRA2B | transformer 2 beta homolog (Drosophila) | 0.034697 | -1.02981 |
| 244279_at | SOBP | sine oculis binding protein homolog | 0.034942 | 1.500198 |
| 1556696_s_at | NR2F1-AS1 | NR2F1 antisense RNA 1 | 0.035045 | 1.125153 |
| 225054_x_at | LINC00674 | long intergenic non-protein coding RNA 674 | 0.035058 | -1.22831 |
| 228547_at | NRXN1 | neurexin 1 | 0.035361 | -1.0456 |
| 1554657_a_at | CFAP61 | cilia and flagella associated protein 61 | 0.035382 | -1.10793 |
| 200930_s_at | VCL | vinculin | 0.035415 | 1.133429 |
| 225207_at | PDK4 | pyruvate dehydrogenase kinase 4 | 0.035503 | 1.148615 |
| 211544_s_at | GHRHR | growth hormone releasing hormone receptor | 0.035514 | 1.040052 |
| 207979_s_at | LOC100996919///CD8B | putative T-cell surface glycoprotein CD8 beta-2 chain-like///CD8b molecule | 0.035528 | 1.19562 |
| 1568736_s_at | DLGAP1 | DLG associated protein 1 | 0.035542 | 1.220101 |
| 236575_at | ARHGEF26-AS1 | ARHGEF26 antisense RNA 1 | 0.035608 | 1.082039 |
| 240336_at | HBM | hemoglobin subunit mu | 0.035637 | 1.175636 |
| 233366_at | FBXO4 | F-box protein 4 | 0.035729 | -1.14204 |
| 211046_at | KCNH6 | potassium voltage-gated channel subfamily H member 6 | 0.035769 | 1.384686 |
| 239671_at | SYT16 | synaptotagmin 16 | 0.035885 | 1.14806 |
| 210843_s_at | MFAP3L | microfibrillar associated protein 3 like | 0.036063 | -1.0083 |
| 206490_at | DLGAP1 | DLG associated protein 1 | 0.0361 | 1.031028 |
| 219996_at | ASB7 | ankyrin repeat and SOCS box containing 7 | 0.036624 | 1.144798 |
| 241805_at | GABRG1 | gamma-aminobutyric acid type A receptor gamma1 subunit | 0.036625 | 1.107178 |
| 205336_at | PVALB | parvalbumin | 0.036639 | 1.613577 |
| 1555689_at | CD80 | CD80 molecule | 0.036694 | 1.48072 |
| 1562095_at | SLMAP | sarcolemma associated protein | 0.036709 | 1.220918 |
| 207613_s_at | CAMK2A | calcium/calmodulin dependent protein kinase II alpha | 0.037153 | 1.046855 |
| 207109_at | POU2F3 | POU class 2 homeobox 3 | 0.037162 | -1.22337 |
| 215459_at | CTNS | cystinosin, lysosomal cystine transporter | 0.03724 | 1.18636 |
| 1568746_a_at | LOC646268 | hCG1654703 | 0.037462 | 1.117921 |
| 1561578_s_at | SLC25A53 | solute carrier family 25 member 53 | 0.037494 | -1.05466 |
| 1557027_at | LOC100287221 | uncharacterized LOC100287221 | 0.037521 | -1.17194 |
| 1554006_a_at | LLGL2 | LLGL2, scribble cell polarity complex component | 0.037598 | 1.285484 |
| 234727_at | DNAH7 | dynein axonemal heavy chain 7 | 0.037798 | -1.03189 |
| 1554558_at | DCAF5 | DDB1 and CUL4 associated factor 5 | 0.03804 | 1.068984 |
| 220172_at | DCAF17 | DDB1 and CUL4 associated factor 17 | 0.038203 | -1.10232 |
| 209830_s_at | SLC9A3R2 | SLC9A3 regulator 2 | 0.038255 | -1.2412 |
| 1566302_at | PPP1R11 | protein phosphatase 1 regulatory inhibitor subunit 11 | 0.038295 | -1.13763 |
| 1552667_a_at | SH2D3C | SH2 domain containing 3C | 0.038303 | 1.042607 |
| 226766_at | ROBO2 | roundabout guidance receptor 2 | 0.038377 | 1.02159 |
| 221846_s_at | CASKIN2 | CASK interacting protein 2 | 0.038541 | 1.063482 |
| 239014_at | CCAR1 | cell division cycle and apoptosis regulator 1 | 0.038713 | -1.24934 |
| 1569554_at | ESR2 | estrogen receptor 2 | 0.039002 | 1.008809 |
| 228935_at | SLC4A8 | solute carrier family 4 member 8 | 0.039552 | 1.316985 |
| 219765_at | ZNF329 | zinc finger protein 329 | 0.03963 | -1.11619 |
| 231021_at | SLC6A19 | solute carrier family 6 member 19 | 0.039718 | 1.024521 |
| 215516_at | LAMB4 | laminin subunit beta 4 | 0.039872 | 1.041797 |
| 221118_at | PKD2L2 | polycystin 2 like 2, transient receptor potential cation channel | 0.039909 | -1.17097 |
| 1561036_at | LOC105373113 | uncharacterized LOC105373113 | 0.039982 | 1.147368 |
| 1563498_s_at | SLC25A45 | solute carrier family 25 member 45 | 0.040042 | -1.09617 |
| 231867_at | TENM2 | teneurin transmembrane protein 2 | 0.040118 | 1.283017 |
| 228979_at | SFTA3 | surfactant associated 3 | 0.040165 | 1.237352 |
| 237215_s_at | TFRC | transferrin receptor | 0.040202 | -1.30426 |
| 1563327_a_at | LINC01545 | long intergenic non-protein coding RNA 1545 | 0.040553 | 1.04759 |
| 223216_x_at | FBXO16///ZNF395 | F-box protein 16///zinc finger protein 395 | 0.040579 | -1.01171 |
| 214636_at | CALCB | calcitonin related polypeptide beta | 0.041062 | 1.157358 |
| 239150_at | SNTN | sentan, cilia apical structure protein | 0.041343 | 1.272605 |
| 244288_s_at | SIX3 | SIX homeobox 3 | 0.04181 | 1.183752 |
| 1558803_at | LOC105371401 | uncharacterized LOC105371401 | 0.041867 | 1.025964 |
| 241482_at | EPHA1 | EPH receptor A1 | 0.041874 | 1.075385 |
| 225746_at | RAB11FIP4 | RAB11 family interacting protein 4 | 0.041904 | 1.103519 |
| 208049_s_at | TACR1 | tachykinin receptor 1 | 0.041973 | -1.09715 |
| 227648_at | SMDT1 | single-pass membrane protein with aspartate rich tail 1 | 0.042012 | 1.003231 |
| 207359_at | CAMKK2 | calcium/calmodulin dependent protein kinase kinase 2 | 0.042188 | 1.028325 |
| 211549_s_at | HPGD | hydroxyprostaglandin dehydrogenase 15-(NAD) | 0.042206 | 1.222548 |
| 234529_at | PCGEM1 | PCGEM1, prostate-specific transcript (non-protein coding) | 0.042313 | 1.200163 |
| 234320_at | CD244 | CD244 molecule | 0.042368 | 1.190567 |
| 228337_at | PWWP2A | PWWP domain containing 2A | 0.042378 | -1.11247 |
| 1553882_at | LINC00504 | long intergenic non-protein coding RNA 504 | 0.042556 | 1.323416 |
| 211430_s_at | MIR8071-2///MIR8071-1///IGHV4-31///IGHM///IGHG2///IGHG1 | microRNA 8071-2///microRNA 8071-1///immunoglobulin heavy variable 4-31///immunoglobulin heavy constant mu///immunoglobulin heavy constant gamma 2 (G2m marker)///immunoglobulin heavy constant gamma 1 (G1m marker) | 0.042608 | 1.285173 |
| 244662_at | MBD5 | methyl-CpG binding domain protein 5 | 0.042629 | -1.07249 |
| 212620_at | ZNF609 | zinc finger protein 609 | 0.042786 | 1.013036 |
| 1554406_a_at | CLEC7A | C-type lectin domain family 7 member A | 0.042866 | -1.22502 |
| 239485_at | CDH4 | cadherin 4 | 0.043158 | 1.035233 |
| 239776_at | TMEM232 | transmembrane protein 232 | 0.043286 | 1.379903 |
| 1570038_at | ZNF595 | zinc finger protein 595 | 0.043744 | -1.19542 |
| 1559685_at | LOC100506379 | uncharacterized LOC100506379 | 0.0438 | 1.095558 |
| 231034_s_at | NHSL1 | NHS like 1 | 0.043834 | 1.084106 |
| 217194_at | RASAL2 | RAS protein activator like 2 | 0.044196 | 1.273921 |
| 228421_s_at | EFEMP1 | EGF containing fibulin like extracellular matrix protein 1 | 0.044284 | 1.166223 |
| 1562227_at | PDE5A | phosphodiesterase 5A | 0.044286 | 1.025462 |
| 1563209_a_at | MACROD2 | MACRO domain containing 2 | 0.044687 | 1.094518 |
| 234433_at | LOC105373460 | uncharacterized LOC105373460 | 0.044879 | 1.174063 |
| 207517_at | LAMC2 | laminin subunit gamma 2 | 0.044891 | 1.194747 |
| 229660_at | SPATA33 | spermatogenesis associated 33 | 0.044998 | 1.135692 |
| 1552304_at | ALG10 | ALG10, alpha-1,2-glucosyltransferase | 0.04504 | 1.211838 |
| 201890_at | RRM2 | ribonucleotide reductase regulatory subunit M2 | 0.045239 | 1.207282 |
| 204712_at | WIF1 | WNT inhibitory factor 1 | 0.045327 | 1.264781 |
| 231099_at | FKBP15 | FK506 binding protein 15 | 0.045359 | 1.19515 |
| 1557443_s_at | HNCAT21 | head and neck cancer-associated transcript 21 | 0.045865 | 1.114916 |
| 233705_at | PACSIN2 | protein kinase C and casein kinase substrate in neurons 2 | 0.045943 | 1.045865 |
| 244863_at | SH3GLB2 | SH3 domain containing GRB2 like endophilin B2 | 0.045992 | 1.000544 |
| 235467_s_at | KCNC4 | potassium voltage-gated channel subfamily C member 4 | 0.046246 | 1.42441 |
| 1563014_at | RPS15 | ribosomal protein S15 | 0.04639 | 1.027829 |
| 210889_s_at | FCGR2B | Fc fragment of IgG receptor IIb | 0.046406 | 1.017969 |
| 1557079_at | ITGBL1 | integrin subunit beta like 1 | 0.046557 | -1.20623 |
| 214469_at | HIST1H2AE | histone cluster 1, H2ae | 0.047005 | 1.217626 |
| 228599_at | MS4A1 | membrane spanning 4-domains A1 | 0.047117 | -1.15611 |
| 241668_s_at | LOC101928728 | uncharacterized LOC101928728 | 0.047204 | -1.14882 |
| 206287_s_at | ITIH4 | inter-alpha-trypsin inhibitor heavy chain family member 4 | 0.047934 | -1.10291 |
| 236973_at | LOC100131662 | uncharacterized LOC100131662 | 0.048021 | 1.096786 |
| 205403_at | IL1R2 | interleukin 1 receptor type 2 | 0.048232 | 1.049668 |
| 221765_at | UGCG | UDP-glucose ceramide glucosyltransferase | 0.04824 | -1.07816 |
| 220614_s_at | ADGB | androglobin | 0.048492 | 1.165943 |
| 209483_s_at | NSL1 | NSL1, MIS12 kinetochore complex component | 0.048512 | 1.303298 |
| 224219_s_at | TRPC4 | transient receptor potential cation channel subfamily C member 4 | 0.048578 | -1.02586 |
| 207518_at | DGKE | diacylglycerol kinase epsilon | 0.048718 | 1.022554 |
| 1562054_at | PPP4R3CP | protein phosphatase 4 regulatory subunit 3C, pseudogene | 0.048762 | 1.347335 |
| 206752_s_at | DFFB | DNA fragmentation factor subunit beta | 0.048861 | -1.03677 |
| 1552792_at | SOCS4 | suppressor of cytokine signaling 4 | 0.049074 | 1.108084 |
| 241534_at | LOC100505549 | uncharacterized LOC100505549 | 0.049098 | 1.069622 |
| 1563668_at | MORN1 | MORN repeat containing 1 | 0.049206 | 1.056676 |
| 243779_at | GALNT13 | polypeptide N-acetylgalactosaminyltransferase 13 | 0.04925 | 1.029142 |
| 1557256_a_at | GABRB1 | gamma-aminobutyric acid type A receptor beta1 subunit | 0.049262 | -1.11745 |
| 1558658_at | ZNF391 | zinc finger protein 391 | 0.049291 | 1.030819 |
| 220361_at | IQCH | IQ motif containing H | 0.049496 | 1.328327 |
| 236645_at | HBP1 | HMG-box transcription factor 1 | 0.049614 | -1.17145 |
| 241937_s_at | WDR4 | WD repeat domain 4 | 0.049679 | 1.125762 |
| 227909_at | SMIM10L2B///SMIM10L2A | small integral membrane protein 10 like 2B///small integral membrane protein 10 like 2A | 0.049719 | -1.08025 |
