# Supplementary material for: Identification of the hub susceptibility genes and related common transcription factors in the skeletal muscle of Type 2 Diabetes Mellitus
Source: BMC Endocr Disord. 2022 Nov 11;22:276. doi: 10.1186/s12902-022-01195-0 (PMC9652898; doi:10.1186/s12902-022-01195-0)
Supplement: Supplementary file 3 — Additional file 3: Figure S1. [file 12902_2022_1195_MOESM3_ESM.pdf]

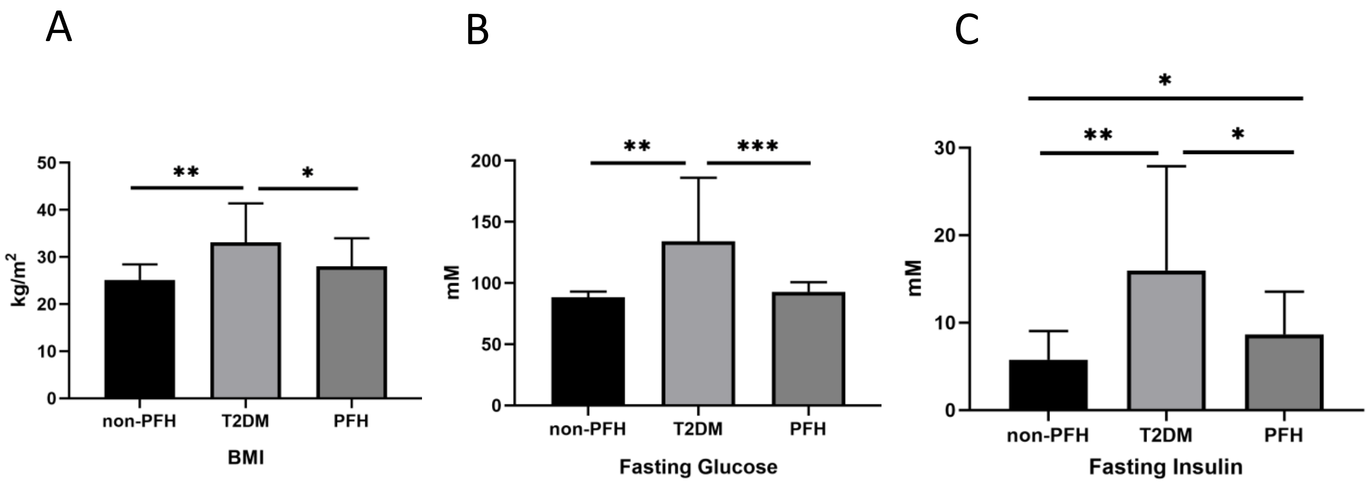

Figure S: BMI and fasting glucose, fasting insulin, in T2DM, PFH and nonPFH subjects. A: The lever of BMI in T2DM, PFH and nonPFH subjects. B: The lever of fasting glucose in T2DM, PFH and nonPFH subjects. C: The lever of fasting insluin in T2DM, PFH and nonPFH subjects. (* $p<0.05$, ** $p<0.01$, *** $p<0.001$).
